# Characterization and Fate of a Septanosyl Ferrier Cation in the Gas and Solution Phases

**DOI:** 10.1021/acs.joc.3c00079

**Published:** 2023-04-24

**Authors:** Kim Greis, Caleb E. Griesbach, Carla Kirschbaum, Gerard Meijer, Gert von Helden, Kevin Pagel, Mark W. Peczuh

**Affiliations:** † Fritz Haber Institute of the Max Planck Society, 14195 Berlin, Germany; ‡ Institute of Chemistry and Biochemistry, Freie Universität Berlin, 14195 Berlin, Germany; § Department of Chemistry, University of Connecticut, 55 North Eagleville Road, U3060, Storrs, Connecticut 06269, United States

## Abstract

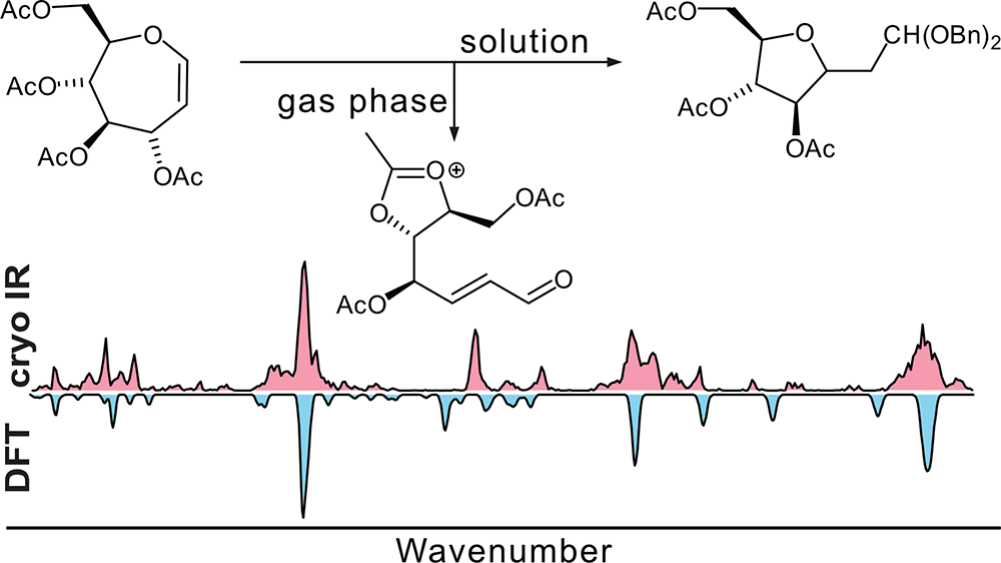

Ferrier reactions follow a mechanistic pathway whereby Lewis acid activation of a cyclic enol ether facilitates departure of an allylic leaving group to form a glycosyl Ferrier cation. Attack on the Ferrier cation provides a new acetal linkage concurrent with the transposition of the alkene moiety. The idiosyncratic outcomes of Ferrier reactions of seven-membered ring carbohydrate-based oxepines prompted an investigation of its corresponding septanosyl Ferrier cation. Experiments that characterized the ion, including gas-phase cryogenic IR spectroscopy matched with density functional theory-calculated spectra of candidate cation structures, as well as product analysis from solution-phase Ferrier reactions, are reported here. Results from both approaches revealed an inclination of the seven-membered ring cation to contract to five-membered ring structures. Gas-phase IR spectra matched best to calculated spectra of structures in which five-membered dioxolenium formation opened the oxepine ring. In the solution phase, an attack on the ion by water led to an acyclic enal that cyclized to a *C*-methylene-aldehydo arabinofuranoside species. Attack by allyl trimethylsilane, on the other hand, was diastereoselective and yielded a *C*-allyl septanoside.

## Introduction

**Figure 1:**
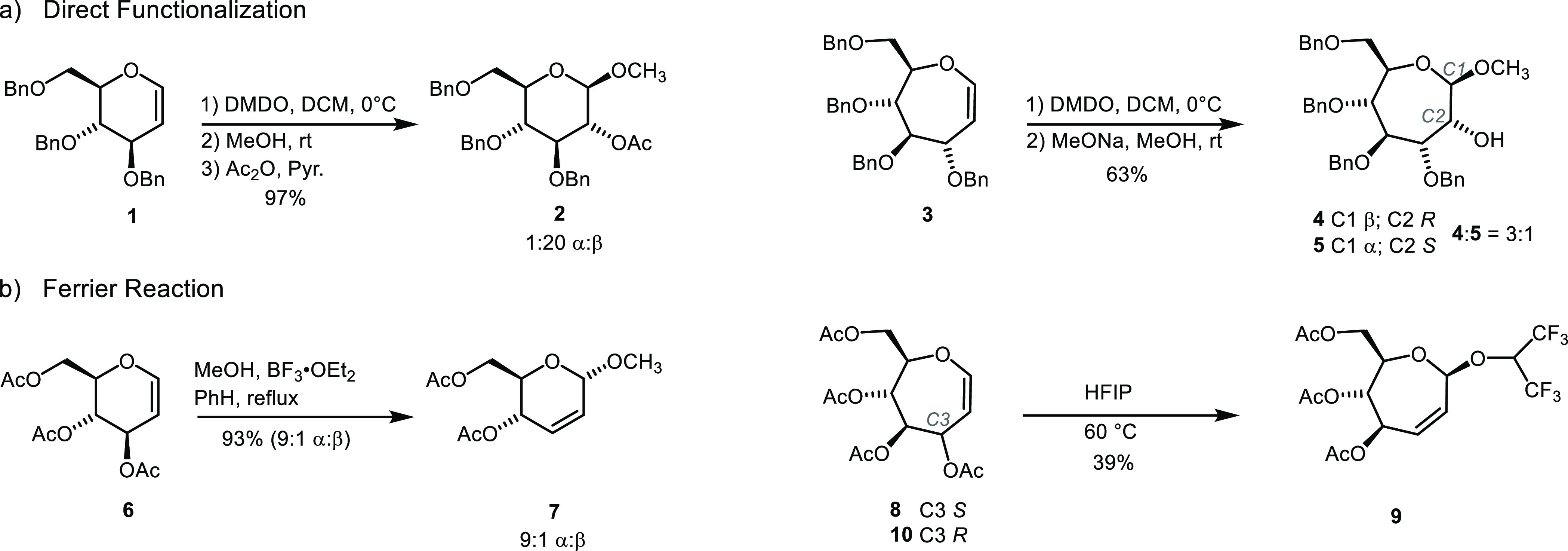
(a) Direct functionalization of d-glucal **1** and d-glucose-based oxepine **3***via* epoxidation and methanolysis; (b) Ferrier reactions of d-glucal **6** and d-glucose-based oxepine **8**; d-mannose-based oxepine **10** does not react under the HFIP Ferrier conditions.

Glycals have proven to be valuable starting materials for the synthesis of numerous oligosaccharides, glycosylated natural products, and even small-molecule targets.^1−3^ The advantages of these compounds come from their inherently rich stereochemistry, the unique reactivity of their enol ether units, and the ability to modulate their reactivity by varying the protecting groups attached to the oxygens.^2,4^ The archetypal reaction of glycals is the functionalization of the double bond with an electrophilic oxygen species (e.g., DMDO) followed by nucleophilic attack on the newly formed 1,2-anhydro sugar (not shown) to form a glycosidic bond, as depicted for the conversion of d-glucal **1** to methyl β-glucoside **2** (Figure 1a).^5,6^ Ring-expanded glycals, informally referred to as carbohydrate-based oxepines (i.e., **3** in Figure 1a), react in a similar fashion. Glucose-based oxepine **3**, for example, was converted to methyl β-d-*glycero*-d-guloseptanoside **4** and α-d-*glycero*-d-idoseptanoside **5** under conditions that were nearly identical to those used for glycals. The diastereomeric mixture of glycosides in the case of the seven-membered ring system mostly reflected the low selectivity of epoxidation of oxepine **3**.^7^ Nonetheless, the common reactivity pattern of glycals and oxepines in terms of the direct addition across the enol ether double bond is apparent.

The Ferrier rearrangement is another reaction typical of glycals (i.e., conversion of **6** to **7** in Figure 1b).^8^ Here, the nucleophilic attack is concomitant with the migration of the double bond as a leaving group is ejected from C3. For the Ferrier reaction, formation of a glycosyl cation under the action of a Lewis acid is essential and nucleophilic attack occurs therefore under S_N_1 conditions. Hence, the stereoselectivity depends on the nature of the glycosyl cation and preferred pathways for additions to it.^9−11^ We recently reported that, under conditions established for glycals, oxepine **8**(12) could be converted to hexafluoroisopropyl 2,3-dideoxy-β-d-*arabino*-hex-2-enoseptanose **9** by a Ferrier rearrangement.^13^ Even though the yield was modest, the reaction reinforced the similarity in reactivity of oxepines to glycals. To our surprise, the C3-epimeric oxepine **10**,^12^ derived from d-mannose, was unreactive under the conditions that afforded the Ferrier product from **8**. The low-energy conformations of **8** and **10** are largely the same—both favor ^4^*H*_6_ conformations with minor populations of twist-half and chair structures. The consequence is that the C3-acetyl group of **8** is pseudoaxial but pseudoequatorial for **10**. We invoked the vinylogous anomeric effect as part of the explanation of this differential reactivity of the oxepines. Furthermore, the preference for the β-anomeric configuration of septanoside **9** was initially unexpected, considering that Ferrier rearrangements with d-glycals have often favored the α-anomer. We speculated that the β-selectivity likely arose either *via* a preference for selective β-attack into a cationic intermediate or *via* anomerization to the thermodynamic product. The latter rationale was reinforced by the susceptibility of the hexafluoroisopropyl group to anomeric stabilization compared to less electron-withdrawing aglycons.^14^

The results from our initial investigation into the Ferrier reactivity of carbohydrate-based oxepines **8** and **10** challenged us to consider in greater detail the cationic intermediate—henceforth referred to as the septanosyl Ferrier cation. This intermediate is generated after the cleavage of the C3 protecting group, with the positive charge formally localized at the C3 atom. The mechanism of glycosylation depends on several factors—especially, the structure of the reactant and the reaction conditions. Depending on the conditions, the mechanism will fall somewhere along an S_N_1–S_N_2 continuum.^15,16^ Traditionally, Ferrier rearrangements were considered to proceed through an allyl oxocarbenium ion intermediate.^2,17^ However, a recent report using cryogenic vibrational spectroscopy in the gas phase revealed that, in isolation, the Ferrier cations generated from acetylated d-glucal and d-galactal exist as dioxolenium ions stabilized by neighboring-group participation (NGP) from an acetyl group at the C4 position on the ring.^18^ In another study, fully protonated Ferrier cations stabilized by superacids were measured by NMR spectroscopy.^19^ Due to their reduced nucleophilicity in this medium, the acetyl groups do not engage in NGP. We reasoned that septanosyl Ferrier cations prepared from oxepines **8** and **10** might similarly be subjected to NGP or long-range participation (LRP, sometimes termed remote participation) which could influence the outcomes of reactions involving them.^20^ Herein, we report a two-pronged approach to investigate the septanosyl Ferrier cation generated from oxepine **8** or **10** in the gas phase using cryogenic vibrational spectroscopy and in the solution phase by Ferrier reactions followed by product characterization. Our investigation reveals a preference for α-attack and subsequent anomerization of the product. In addition, we observe the preference of the septanosyl Ferrier cation to ring-contract to a thermodynamic product in the gas and condensed phases.

**Figure 2:**
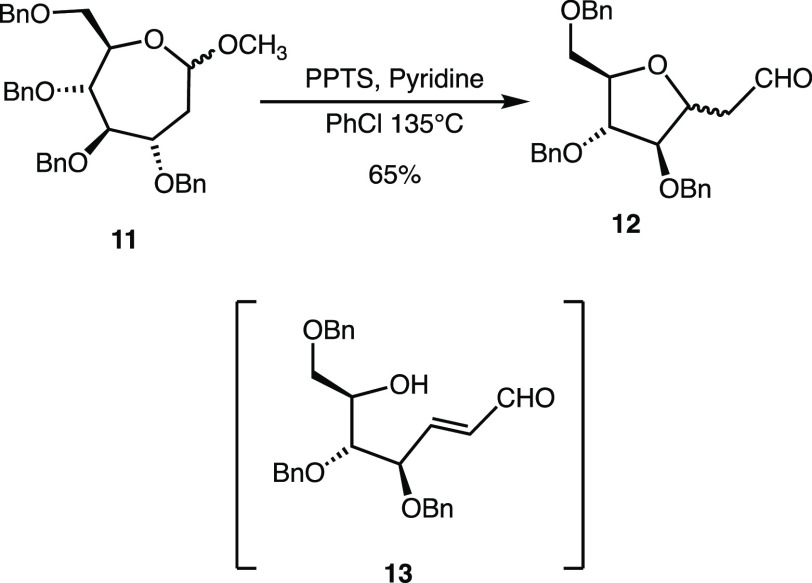
Previously reported ring contraction of methyl 2-deoxyseptanoside **11** to *C*-methylene-aldehydo arabinofuranoside **12** under acidic conditions *via* a putative enal intermediate **13**.

Previous reports from our group chronicle a proclivity toward complex reactivity by cationic septanosyl intermediates. Notable among these instances were intramolecular reactions that formed bicyclic products.^11,21^ We also reported a ring contraction in which methyl septanoside **11** was converted to substituted *C*-methylene-aldehydo arabinofuranoside **12** (Figure 2).^22^ This unexpected product arose under conditions aimed at performing a regioselective, acid-mediated elimination of methanol across the C1–C2 bond of **11** to deliver oxepine **3**. An α,β-unsaturated aldehyde, **13**, was invoked as a likely intermediate in the transformation. Enal **13** underwent *oxa*-Michael addition to form a unique *C*-methylene-aldehydo arabinofuranoside **12**. During the investigation of the septanosyl Ferrier cation reported here, we observed a similar ring contraction, as detailed in the Results and Discussion. Taken together, the two examples of ring contractions highlight a hierarchy of thermodynamic stabilities where seven-membered rings are less stable compared to five- and six-membered rings. This hierarchy is not unique to systems where ring contractions are a thermodynamic sink.^23^ In fact, dynamic equilibria between minor seven- and major five-membered ring products can be observed in aqueous media.^24,25^

## Results and Discussion

### Characterization of a Septanosyl Ferrier Cation in the Gas Phase

**Figure 3:**
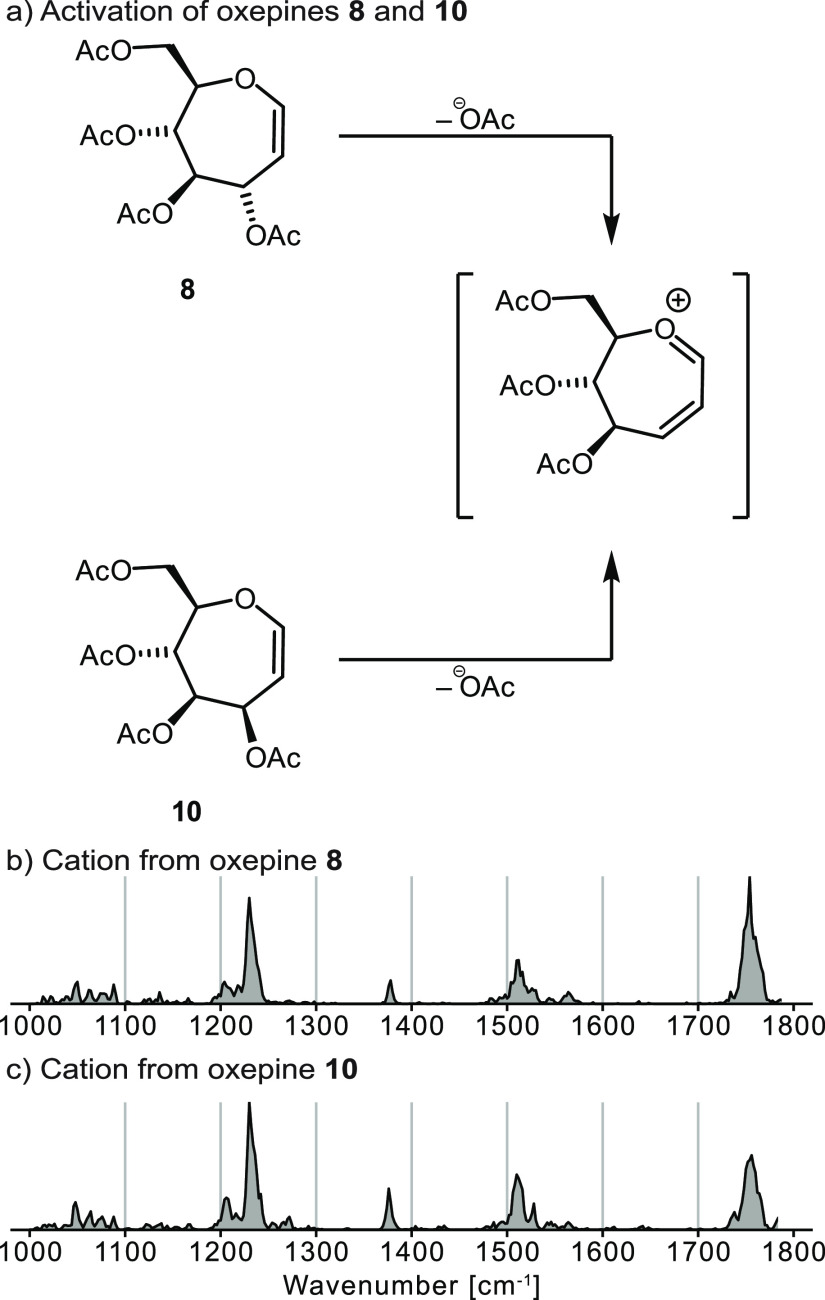
(a) Cleavage of the C3-acetoxy group from oxepines **8** and **10** lead to the same septanosyl Ferrier cation. Cryogenic infrared spectroscopy in helium nanodroplets of septanosyl Ferrier cations [M – OAc]^+^ generated from (b) glucose- and (c) mannose-derived per-*O*-acetyl oxepines **8** and **10**, respectively, reveals the identity of both cations.

Nano-electrospray ionization (nESI) of per-*O*-acetyl oxepines **8** and **10**, derived from glucose and mannose, respectively, yielded three main signals at *m*/*z* 285, 367, and 711 (Figure S1). These signals correspond to [M – OAc]^+^, [M + Na]^+^, and [2M + Na]^+^ ions. The [M – OAc]^+^ ion most likely arises from cleavage of the C3-acetoxy group, leading to a Ferrier-like carbocation (Figure 3a). Traditionally, such ions can be stabilized by resonance and/or by the participation of one of the remaining acetyl groups. Based on the mass spectrum alone, oxepines **8** and **10** cannot be differentiated. Furthermore, the septanosyl Ferrier cations generated from both precursors should be identical as they only differ in the absolute configuration of the group at C3, which is cleaved upon activation.

To find out if the septanosyl Ferrier cations generated from oxepines **8** and **10** are identical and what their structure is, they were investigated using cryogenic infrared (IR) spectroscopy (Figure 3b,c). Recently, this technique was used to investigate the structure of Ferrier cations generated from glycal precursors^18^ and to probe intramolecular interactions in various glycosyl cations.^26−29^ The IR spectra displayed in Figure 3b reveal that both ions are identical as their IR signatures are essentially superimposable. Hence, as anticipated, cleavage of the C3-acetoxy group gives rise to the same cation. Generally, the vibrations observed in the fingerprint region around 1000–1300 cm^–1^ can be assigned to C–C and C–O stretches, whereas the absorption bands observed in 1300–1450 cm^–1^ originate from C–H bends. As has been observed in related systems,^18^ the functional group region contains symmetric and antisymmetric dioxolenium (COO^+^), oxocarbenium (C=O^+^), and C=C-stretches in the 1450–1700 cm^–1^ range, while carbonyl (C=O) stretches are commonly found around 1700–1800 cm^–1^.

**Figure 4:**
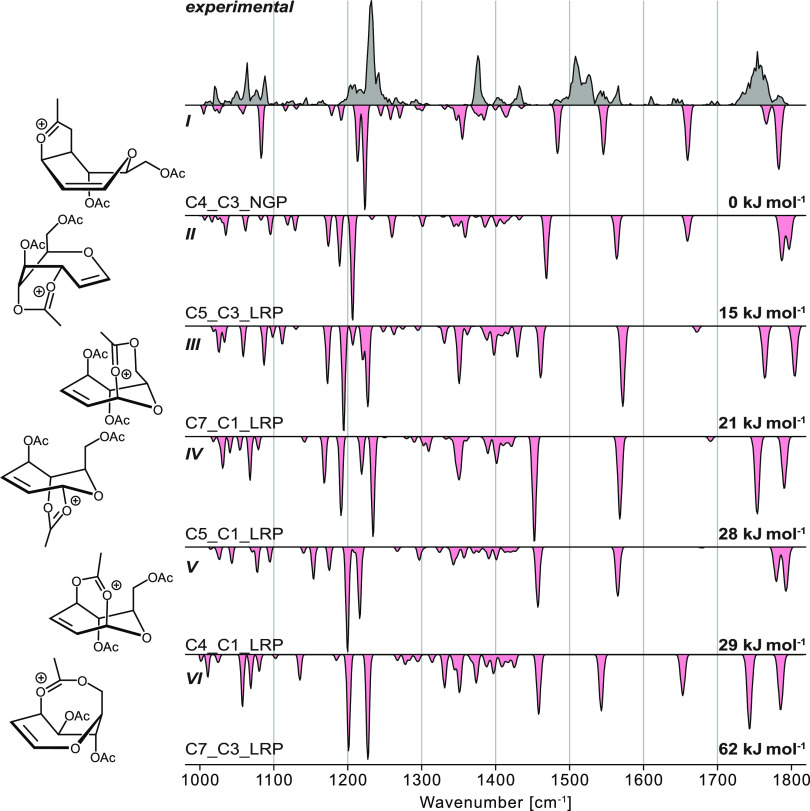
Experimental infrared spectrum (gray) of the septanosyl Ferrier cation [M – OAc]^+^ compared to computed spectra (red, inverted traces) of structures exhibiting (**I**) NGP of the C4-acetyl group at the C3 position, (**II**) LRP of the C5-acetyl group at the C3 position, (**III**) LRP of the C7-acetyl group at the C1 position, (**IV**) LRP of the C5-acetyl group at the C1 position, (**V**) LRP of the C4-acetyl group at the C1 position, and (**VI**) LRP of the C7-acetyl group at the C3 position. The relative free energy at 90 K as well as schematic depictions of each structure are indicated.

To get insight into the structure of the Ferrier-like ion, the experimental spectrum was compared to computed spectra derived from harmonic frequency calculations for several possible structural motifs. Structural motifs that were considered made use of the C4-, C5-, or C6-acetyl groups to stabilize the positive charge of the oxocarbenium ion *via* NGP or LRP. Because the charge of the Ferrier-like cation is formally delocalized along the four-atom O–C3 unit of the septanose ring, the acetyl groups could participate at both C1 and C3 positions. It has been determined that such structures—where the positive charge is stabilized by NGP of the C4-acetyl group—are adopted by Ferrier glycosyl cations based on pyranose sugars.^18^ Geometries for each structural motif were built and their conformational spaces were sampled. For each motif, a subset of low-energy structures was selected for reoptimization and computation of harmonic frequencies at a higher level of theory PBE0+D3/6-311+G(d,p).^30−33^ For the lowest-energy structure of each motif, more accurate single-point energies were obtained at the DLPNO-CCSD(T)/Def2-TZVPP^34−36^ level of theory. Overall, similar to pyranose-based Ferrier cations, the overall lowest-energy structure is a cation in which the charge at C3 is stabilized by the NGP of the C4-acetyl group (**I**). Hence, this lowest-energy structure serves as a reference. Computed IR spectra of the lowest-energy structure for each of the six structural motifs are depicted in Figure 4.

Based on the lowest-energy structures for each structural motif, the stability decreases in the following order C4_C3_NGP (**I**) > C5_C3_LRP (**II**) > C7_C1_LRP (**III**) > C5_C1_LRP (**IV**) > C4_C1_LRP (**V**) > C7_C3_LRP (**VI**) (Figure 4). This ranking indicates that the relative stability of the respective structural motif is dependent on the ring size of the newly formed ring after participation, which are five- (**I**), six- (**II**), seven- (**III**, **IV**, **V**), and eight-membered rings (**VI**). Relative to **I**, the other interactions are destabilized by 15–62 kJ mol^–1^. Generally, and similar to previous studies,^18,28^ NGP is always favored over LRP.

Previously, the identity of the two septanosyl Ferrier cations was confirmed based on their experimental IR spectra. To assign a computed structure to the experimental spectrum, it has been rerecorded with a higher power of the free-electron laser, leading to a better-resolved spectrum (Figure 4). The experimental spectrum is significantly crowded in comparison to the computed spectra, suggesting that the ensemble of previously mass-to-charge selected ions was composed of more than one conformer. It is possible that there was more than one structural motif present in the ion trap. While some harmonic frequencies of the intermediate exhibiting NGP (**I**) have matching absorption bands to the experimental spectrum, they are low in intensity. Hence, such a structure may be present in the ion trap but only to a lesser extent. The computed IR spectra of the other species match even less well with the experimental spectrum than the spectrum of **I**. Thus, based on their poor match and their unfavorable energetics, these structures can be discarded. Furthermore, the lowest-energy structure—if present among these—is only partially populating the ion trap.

**Figure 5:**
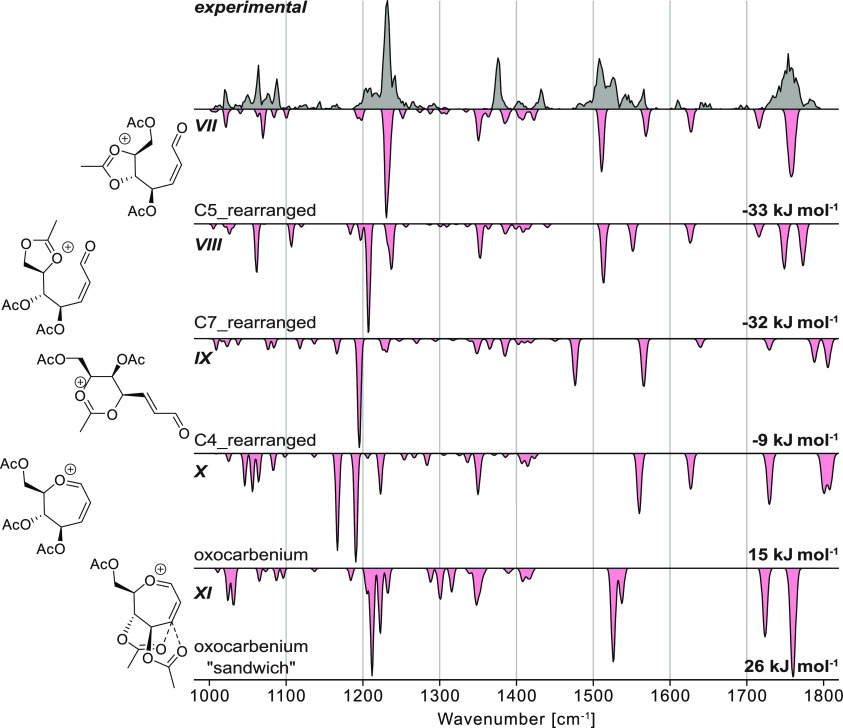
Experimental infrared spectrum (gray) of the septanosyl Ferrier cation [M – OAc]^+^ compared to computed spectra (red, inverted traces) of structures exhibiting rearrangement by the attack of the (**VII**) C5-, (**VIII**) C7-, and (**IX**) C4-acetyl groups at the C6 position leading to ring opening, (**X**) oxocarbenium structures that are stabilized by one acetyl group interacting *via* long-range, or (**XI**) oxocarbenium-type structures stabilized by long-range interaction of two acetyl groups. The relative free energy at 90 K as well as schematic depictions of each structure are indicated.

Due to the unsatisfactory structural match of **I** with the experimental spectrum, other structural motifs were considered as well. Recent publications reported that rearrangement occurs for certain pyranose-based glycosyl cations in the gas phase.^29,37^ There, an acetyl group attacks the C5 carbon atom of a pyranose, leading to the opening of the pyranose ring and the formation of a five-membered dioxolenium moiety and an aldehyde group. Such dioxolenium ions have previously been stabilized in super acids, where they rearranged to oxonium ions.^38^ In our system, rearrangement could potentially arise from the attack of each of the acetyl groups at C4, C5, and C7 onto the C6 atom of the seven-membered ring. Mechanistically, such an attack would proceed *via* an S_N_2 mechanism, hence leading to inversion of the stereoconfiguration at C6. Therefore, the configuration at C4/C5/C6 of the rearranged septanosyl Ferrier cations would be (*R*,*S*,*S*). Although mechanistically less likely, the C6-epimers of the rearranged ions were considered as well. Additionally, species that did not employ the participation of an acetyl group—oxocarbenium structures—were investigated. Similar to the previously considered structures, the conformational space of the new structural motifs was sampled, a subset of low-energy structures was reoptimized, and harmonic frequencies were computed at a higher level of theory. Computed IR spectra of the lowest-energy structures in comparison to the experimental spectrum are depicted in Figures 5 and S2. It is apparent that the rearranged species are significantly lower in energy by 9–33 kJ mol^–1^ than the one stabilized by NGP (**I**). The oxocarbenium structures are higher in energy relative to **I**. The computed energetics of the rearranged structure formed by the C5-(**VII**) or the C7-acetyl group (**VIII**) are very similar; however, the computed spectrum of the C5_rearranged structure matches the experimental spectrum slightly better. Here, mainly the carbonyl stretches of the free acetyl groups at 1761 and 1756 cm^–1^, the symmetric and antisymmetric dioxolenium stretches at 1511 and 1569 cm^–1^, and the C–O stretch at 1230 cm^–1^ match exceptionally well. C4_rearranged structures (**IX**) or the lowest-energy oxocarbenium-type structures (**X** and **XI**) generally match less well. However, in one oxocarbenium structure, the charge center is “sandwiched” by two acetyl groups (**XI**) (Figure S5), leading to a strong change in IR absorption. The gaps in the experimental spectrum that cannot be filled by **VII** are, based on the energetics, most likely filled by structure **VIII**, while matching absorption bands can also be observed for the higher-energy structures **I** and **XI**.

Transition states leading to the rearranged structures (Figure S3a) indicate a comparably low barrier for rearrangement of ca. 68–84 kJ mol^–1^. This value is in line with previously computed transition states for rearrangement of pyranose-based glycosyl cations (35–138 kJ mol^–1^).^29^ The required energy for the rearrangement is transferred to the ion during the ion-source fragmentation process. The C5- and C7-rearranged species **VII** and **VIII** cannot directly interconvert into each other. However, the (*R*,*S*,*S*) (C4/C5/C6) diastereomer of the C5-rearranged ion can convert to the (*R*,*S*,*R*) diastereomer of the C7-rearranged ion *via* S_N_2 attack of the C7-acetyl group at C6. The same reaction can proceed for the (*R*,*S*,*S*) diastereomer of the C7-rearranged ion. The barrier of this reaction is computed to be only 61–62 kJ mol^–1^ (Figure S3b). However, the formation of the diastereomers is thermodynamically not favored.

Overall, our assessment of the experimental and computational data in the gas phase is that the most abundant species observed is the ring-opened ion **VII** formed by the attack of the C5-acetyl group at the C6 position. However, there are peaks in the experimental region that are broader than predicted by theory and some peaks are not reproduced at all by ion **VII**. Therefore, it is likely that a fraction of the probed ions adopt other structures, such as C7_rearranged (**VIII**). Importantly, the fact that the seven-membered ring system followed trajectories on the potential energy surface to form the five-membered rings was significant. The propensity for seven-membered ring cations to decompose into the more stable five-membered rings cannot only be observed in the gas phase but also in solution-phase experiments *via* a different mechanism for ring opening (*vide infra*).

### Characterization of the Septanosyl Cation in Solution via Product Analysis of Ferrier Reactions

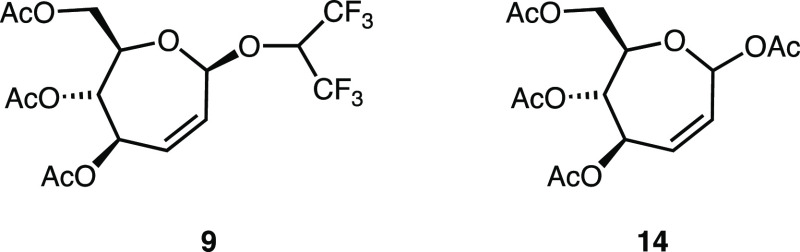



**Figure 6:**
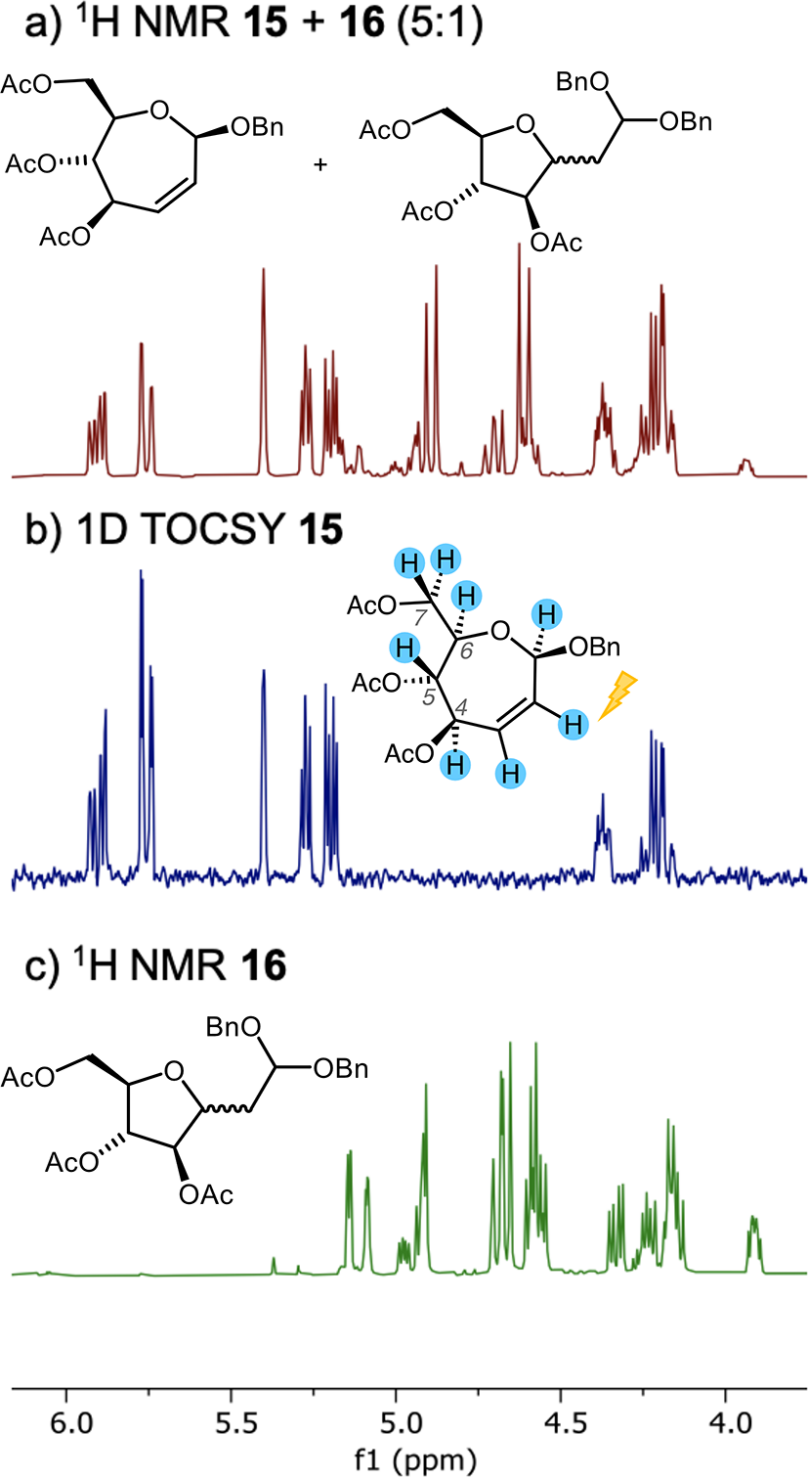
(a) ^1^H NMR spectrum of an isolated fraction of the anhydrous Ferrier rearrangement containing **15** and **16** (5:1); (b) selective 1D TOCSY spectrum of **15** arising from irradiation of the H2 chemical shift region (δ 5.76 ppm) on a sample of **15** + **16** (5:1); (c) ^1^H NMR spectrum of major product **16** arising from the “wet” Ferrier rearrangement.

**Scheme 1:**
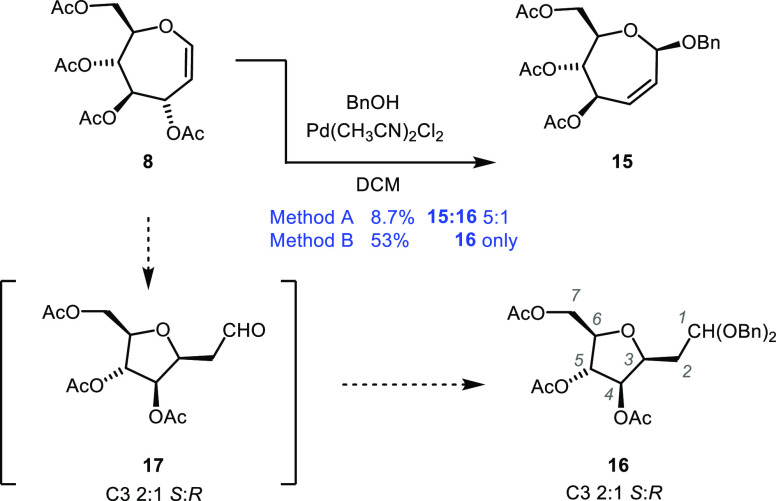
Pd(II)-Mediated Ferrier Reaction and Ring Contraction

Previous Ferrier reactions of oxepine **8** using alcoholic nucleophiles were of mixed success. Initial reactions of **8** with benzyl alcohol in the presence of Lewis acids (i.e., FeCl_3_ and BF_3_·OEt_2_) gave intractable product mixtures. On the other hand, HFIP septanoside **9** and septanose acetate **14** were prepared in modest yields (39 and 26%, respectively)^13^ under less common conditions.^39,40^ We then turned to a palladium-mediated rearrangement introduced by Galan and Sau.^2^ Care was taken to dry donor **8** and benzyl alcohol by azeotropic distillation before dissolving in anhydrous dichloromethane. Addition of vacuum-desiccated Pd(MeCN)_2_Cl_2_ to the solution while under a nitrogen atmosphere (Method A) resulted in the disappearance of the starting material by TLC and the appearance of two new spots. One fraction isolated by chromatography in low yield (8.7%) proved to be a 5:1 mixture of Ferrier product **15** and *C*-methylene-acetal arabinofuranoside **16** (Scheme 1 and Figure 6a). The similarity of the olefinic ^1^H signals for H2 and H3 in the mixture to those of earlier Ferrier product **9** helped to assign the structure of **15**. Irradiation of H2 in a selective TOCSY experiment was used to set the signals of the seven-membered ring. The β-configuration of the anomeric center was assigned based on its similarity to the C1 chemical shift of **9** and was reinforced by an NOE cross peak between H1 and H6 (see the Supporting Information). Structural assignment of **16** was done retroactively based on additional experiments (*vide infra*). The other product fraction from chromatography was *C*-methylene-aldehydo arabinofuranoside **17**. Compound **17** proved to be unstable in our hands; we were, however, able to conduct an explicit experiment to isolate it (12% BRSM)^41^ and collect NMR spectra used in its structural assignment. Analysis of the data revealed that **17** was isolated as a 2:1 mixture of stereoisomers at the C3 position (Scheme 1). Observation of this species suggested both the likely structure of **16** and an experiment to prepare it.

The appearance of the *C*-methylene-aldehydo compound **17** was reminiscent of compound **12** (Figure 2) that had arisen under reaction conditions where an oxocarbenium ion was a plausible intermediate. In that previous case, adventitious water was implicated in formation of the ring-contracted compound. Running the palladium-mediated Ferrier reaction under conditions where measures to remove water were not taken (Method B) consequently resulted in the isolation of compound **16** as the sole product in a 53% yield (BRSM). In fact, it was the analysis of NMR spectra of the sample of **16** (Figure 6c) obtained under these conditions that enabled its assignment as a product in the earlier anhydrous reaction. Presumably, attack on the septanosyl Ferrier cation by water forms a lactol that tautomerizes to the unsaturated aldehyde; *oxa*-Michael addition by the C6 hydroxyl then leads to *C*-methylene aldehyde species **17**, followed by acetalization to provide **16** as a 2:1 mixture of C3 diastereomers (2:1 *S*/*R*. The major isomer is shown in Scheme 1). See the Supporting Information for additional spectroscopic details on the structure of **16**.

**Figure 7:**
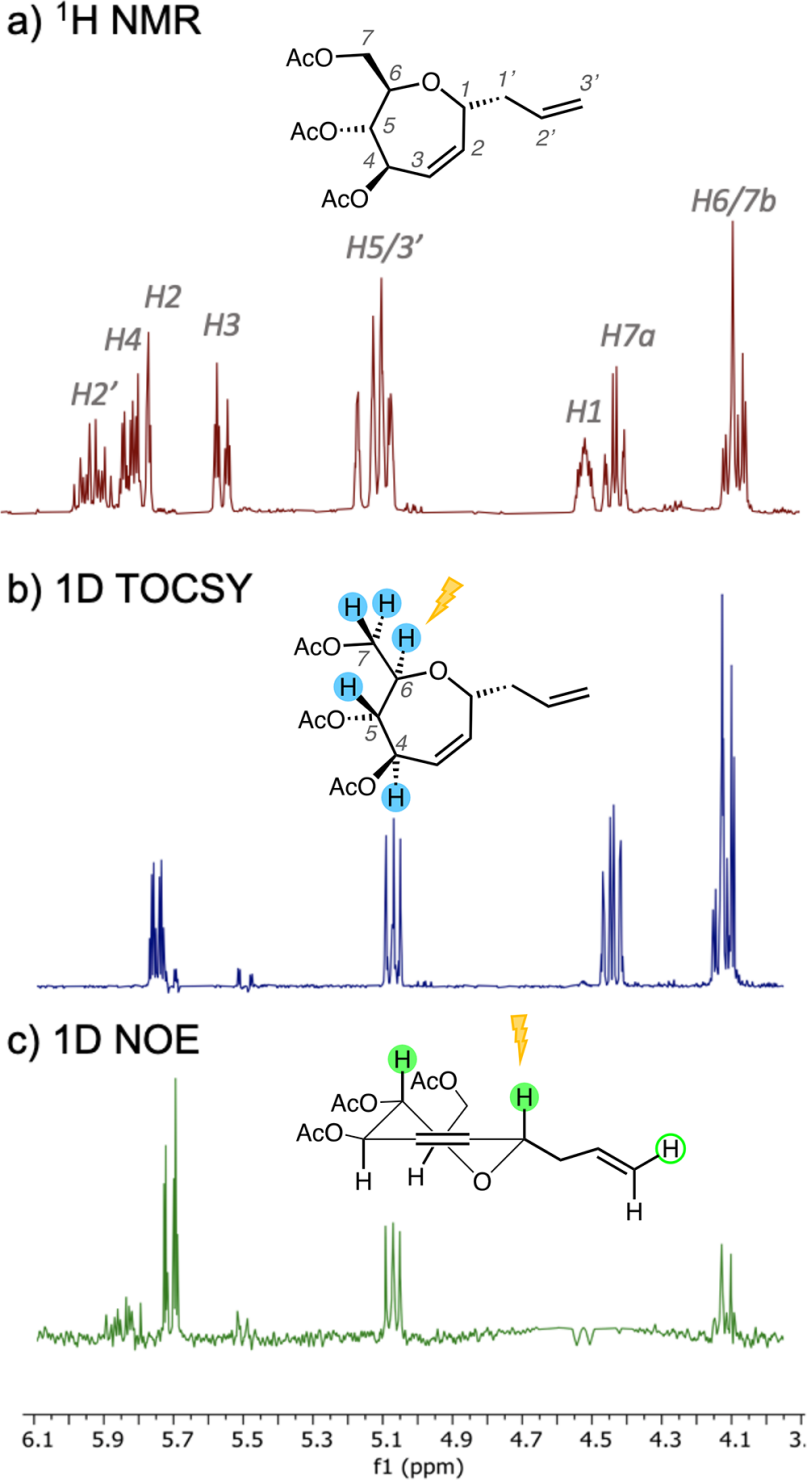
(a) Detail of the ^1^H NMR spectrum of α-*C*-allyl septanoside **18** with signals assigned according to the structure shown. (b) 1D TOCSY spectrum of **18** arising from irradiation of the H6/H7b chemical shift region (δ 4.02 ppm); the structure highlights protons assigned *via* spin diffusion. (c) 1D NOE spectrum of **18** arising from irradiation on H1.^42^ NOE assignments based on the ^5^*H*_O_ conformer of α-*C*-allyl septanoside **18**.

**Scheme 2:**
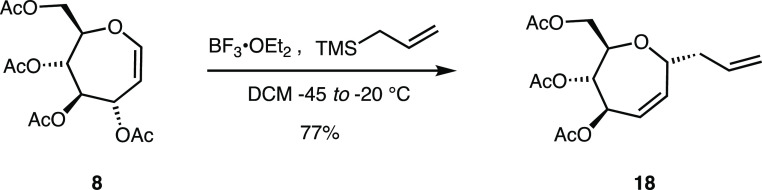
Allylation Reaction on Oxepine **8** to Form α-C-Allyl Septanoside **18**

Additional evidence of the septanosyl Ferrier cation in the solution phase was inferred by characterizing the product of a kinetic trap experiment. Allylation was used because the stereocenter formed in the reaction reflects the facial selectivity of attack and is unable to equilibrate to a thermodynamic product.^11,19^ The reaction was performed on oxepine **8** (Scheme 2), where reagents were added at −45 °C. The reaction was allowed to warm to −20 °C and held at that temperature for 1 h. A single product was isolated from the reaction mixture, in a 77% yield, whose NMR spectra proved to be consistent with allyl *C*-septanoside **18**. In deuterochloroform, the ^13^C{^1^H} NMR spectrum of the product showed one set of signals, indicating that a single diastereomer was the product of the reaction. The ^1^H NMR spectrum, however, suffered from overlapping signals that prevented the analysis of ^3^*J*_H,H_ coupling constants and H,H NOEs to characterize which stereoisomer had formed. When the solvent was changed to acetone-*d*_6_ (Figure 7a), several of the signals became sufficiently resolved to enable analysis of NOEs. Both 1D and 2D ^1^H–^1^H NOESY NMR spectra showed NOEs between H1 (δ 4.44 ppm) and a signal (δ 5.04 ppm) that corresponded to H5 and/or a vinylic signal from the allyl group (H3′). To tease out if H1 was in close proximity to both or just one of these protons, a selective 1D TOCSY experiment was implemented. Irradiation of the chemical shift region shared by signals for H6 and H7 (δ 4.02 ppm) identified the spin system corresponding to H4 through H7 and enabled the assignment of H5 (Figure 7b). In light of that assignment, and with regard to the likely preference for the ^5^*H*_O_ conformation^13^ of the compound, it was clear that it was H1 that overlapped with H5 in the NOE experiments (Figure 7c). Based on this NOE and the known configuration of C5, we assigned the product as the α-anomer, compound **18**.

The α-configured *C*-glycoside **18** formed in the kinetic trap experiment was of the opposite configuration of HFIP glycoside **9** that we had observed previously, as well as benzyl septanoside **15**. This result suggested that, in *O*-glycosylation reactions, the product equilibrates to the thermodynamically favored anomer. Furthermore, the selective formation of the α-anomer was consistent with a similar kinetic trap experiment where d-glucal was used as the starting material and the α-configured *C*-allyl glucoside was isolated as a product. Concomitant with the results of the kinetic trap experiment, ^13^C{^1^H} NMR spectra were collected in superacid media in conjunction with density functional theory calculations and used to characterize a protonated oxocarbenium ion exhibiting a β-face that was significantly hindered by the C6 acetoxymethyl group.^19^ Looking at the calculated structures of oxocarbenium ion conformers, the C4_C3_NGP **I** and the oxocarbenium “sandwich” species **XI** should be quite similar; an attack on either of them should favor the α-face because it minimizes transannular interactions between the ring and the nucleophile (Figure S6).^9,10^ Particularly, C4_C3_NGP **I**—the lowest-energy cation—has a β-face hindered by the participating acetate and acetoxymethyl group. The α-face of cation **I** is unhindered, and nucleophilic attack is expected to be highly stereoselective; however, other ions have similar profiles. Upon generation of an oxocarbenium ion, stabilization of the electrophilic C1 and C3 positions by the C4 and C7 acetates would effectively block its β-face. Also, the α-product probably adopts a half-chair conformation that projects substituents in a quasi-equatorial arrangement.

## Conclusions

The propensity for ring opening of the septanosyl Ferrier cation is the common theme that emerged from the gas- and solution-phase experiments reported here. IR spectra of the gas-phase ion match with computed spectra where an acetyl group at either C5 or C7 attacks at C6, rupturing the septanose ring to form a five-membered dioxolenium ion (i.e., cations **VII** and **VIII** in Figure 5) with a pendant enal moiety. These dioxolenium enals were also calculated to be among the most stable structures of the studied cations. Previously, a similar rearrangement was reported in the gas phase for pyranose-based glycosyl cations.^29,37^ In the solution phase, Ferrier product benzyl septanoside **15** was only obtained in low yield and it was accompanied by the ring-opened *C*-methylene dibenzyl acetal **16**. Ring opening was facilitated by the attack of adventitious water onto the septanosyl Ferrier cation. The subsequent septanose lactol then relaxed to the acyclic enal followed by an *oxa*-Michael attack, delivering *C*-methylene-aldehydo species **17**. Under conditions where measures to remove water were abandoned, ring-contracted product **16** predominated. Critically, the septanosyl Ferrier cation could be efficiently trapped under kinetic conditions. Using allyl trimethylsilane as a nucleophile *C*-allyl septanoside **18** was obtained with good yield and diastereoselectivity. In total, the results suggest that Ferrier reactions of oxepines **8** and **10** with alcoholic nucleophiles will be vexed by low yields, but they should be amenable to formation of other *C*-glycosides.^43^ Finally, the results from the gas and solution phases both point to the formation of a stable five-membered ring from a less stable seven-membered ring. While the relative stabilities of these rings (in combination with six-membered rings) are considered to be well established,^24,25^ specific examples demonstrating them are rare.

## Experimental Section

### General Methods

Commercially available reagents were used without further purification with the exception of benzyl alcohol which was checked for benzaldehyde before use in experiments and distilled over potassium hydroxide when necessary. Solvents for anhydrous reactions were dried over calcium hydride and distilled. Solid reagents were dried in a vacuum desiccator in the presence of phosphorous pentoxide as a desiccant prior to use. Compounds not purchased were synthesized in accordance with the literature precedent and matched reported spectra. Structural assignments were made with additional information from gCOSY, gHSQC, and gHMBC experiments.

### Mass Spectrometry and Cryogenic Infrared Spectroscopy

Acetylated oxepines derived from glucose- and mannose-based oxepines were dissolved in a mixture of acetonitrile and water (9:1, V/V) to yield 100 μm solutions. The oxepines were ionized by nano-electrospray ionization (nESI) on a custom-built mass spectrometer that allows for infrared ion spectroscopy in helium nanodroplets, previously described in detail.^44−46^ For nESI, Pd/Pt-coated glass capillaries prepared in-house were used. Septanosyl Ferrier cations are generated by in-source fragmentation of protonated or sodiated oxepines. The ion beam is focused by two ring-electrode ion guides, and the ions of interest are mass-to-charge-selected in a quadrupole mass filter. Subsequently, the ions are guided to a quadrupole bender, where the ions either pass through to a time-of-flight detector to monitor the ion signal and record mass spectra or are bent into a hexapole ion trap. Here, the ions are thermalized by buffer gas cooling to the temperature of the ion trap (90 K) achieved by cooling with liquid nitrogen.

A beam of superfluid helium nanodroplets (0.37 K) is generated by a pulsed Even-Lavie valve (nozzle temperature of 21 K). The helium nanodroplets pass through the hexapole ion trap, picking up the ions and guiding them to a detection region where the beam of doped helium nanodroplets overlaps with an infrared (IR) beam of the Fritz Haber Institute free-electron laser (FHI FEL).^47^ Infrared radiation leads to the excitation of resonant vibrational modes of the analyte ions. By relaxation, the energy is dissipated to the helium matrix that subsequently evaporates. The helium matrix acts as a cryostat that keeps the ions at 0.4 K. After the absorption of multiple IR photons, the ion is released from the droplet and detected by a time-of-flight detector. Monitoring the ion yield as a function of the IR wavenumber leads to an IR spectrum. The ions were probed in the 1000–1800 cm^–1^ range.

### Computational Methods

To assign a structure to the intermediate ion characterized by infrared ion spectroscopy, the conformational space of potential candidates was sampled using the software CREST^48^ (version 2.9) with the semiempirical method GFN2-xTB,^49^ the empirical method GFN-FF^50^ (using xtb version 6.3.0) and Schrödinger Maestro^51,52^ (version 2021-3). As the C3-acetyl group in the oxepines is cleaved, several structural motifs are conceivable (displayed in Figures 4 and 5). The conformational spaces of non-rearranged dioxolenium-type structures exhibiting long-range or NGP were sampled using CREST with GFN2-xTB, while the other structures were sampled using Maestro and CREST with GFN2-xTB/GFN-FF. Sampling these other structures in CREST with GFN2-xTB is nontrivial as these structures often tend to rearrange or form erroneous bonds during sampling.

Oxocarbenium and rearranged dioxolenium ions were loaded into Schrödinger Maestro.^51,52^ A Monte Carlo search using the OPLSe forcefield in vacuum was performed to sample the conformational space for each ion. Newly found conformers within 63 kJ mol^–1^ were tested by an rmsd statistic. Conformers with an rmsd > 0.5 Å from all previously generated conformers were considered unique. These were then optimized in Maestro at a PBE0+D3/6-31G(d) level of theory and again tested for uniqueness by an rmsd statistic.

All geometries generated by the CREST sampling were optimized at the PBE0+D3/6-31G(d)^30−33^ level of theory implemented in Gaussian 16.^53^ All unique structures optimized at the PBE0+D3/6-31G(d) level of theory below 21 kJ mol^–1^, relative to the lowest-energy structure of one structural type, were reoptimized, and harmonic frequencies were computed at the PBE0+D3/6-311+G(d,p) level theory in Gaussian 16. The relative free energy at 90 K (Δ*F*_90K_, according to the temperature of the ion trap) from the harmonic frequency calculation was used to rank all final structures (Table S1 and Figures S4 and S5). All harmonic infrared spectra were scaled by an empirical scaling factor of 0.965. For the lowest-energy structure of each motif, single-point energy calculations at the DLPNO-CCSD(T)/Def2-TZVPP^34−36^ level of theory were performed in ORCA 5.0.3^54^ (Table S2). The xyz coordinates of the reoptimized geometries can be found in the Supporting Information.

Transition states were located using relaxed scans of the reaction coordinate in Gaussian 16. The saddle points were optimized as transition states, and the harmonic frequencies were computed at the PBE0+D3/6-311+G(d,p) level of theory. The existence of one imaginary frequency corresponding to the reaction coordinate confirms the existence of the transition state. The transition states were linked to minima using intrinsic reaction coordinate calculations (Figure S3). Single-point energies of all optimized structures along the reaction trajectory were computed at the DLPNO-CCSD(T)/Def2-TZVPP level of theory using ORCA.

### Reactions of Oxepine 8

#### Method A

(Ferrier reaction under anhydrous conditions) To a 10 mL round-bottom flask were added oxepine **8** (120.9 mg, 0.351 mmol) and benzyl alcohol (40.0 μL, 0.385 mmol, 1.1 equiv). The contents were dried by azeotropic distillation with toluene (3 × 2 mL) and then dissolved in dry DCM (2 mL) under N_2_ at rt. Then, bis(acetonitrile)dichloropalladium(II) (9.1 mg, 0.035 mmol, 0.1 equiv), dried in a vacuum desiccator prior to use, was added as a solid in one portion. After 5 h, the reaction was quenched with aq. NaHCO_3_ (1 mL). The mixture was diluted with DCM (10 mL) and sequentially washed with saturated NaHCO_3_ (1 × 10 mL), water (1 × 10 mL), and brine (1 × 10 mL). The organic layer was dried with Na_2_SO_4_ and filtered, and the solvent was removed under reduced pressure. The residue was purified by column chromatography (40% EtOAc/hexane) to afford 12.6 mg (8.7%) of a yellow syrup which was a mixture of **15** and **16** (5:1).

#### Method B

(Ferrier reaction without rigorous exclusion of water) To a 10 mL round-bottom flask, **1** (24.0 mg, 0.0726 mmol) and benzyl alcohol (8.0 μL, 0.077 mmol, 1.1 equiv) were added. The catalyst bis(acetonitrile)dichloropalladium(II) was added as a solution in dichloromethane (0.40 mL, 4.42 mg mL^–1^, 0.1 equiv). The solution was stirred open to the atmosphere for 5 h. Then, saturated aq. NaHCO_3_ (1 mL) was added to the reaction (quench), and this mixture was diluted with dichloromethane (10 mL). The solution was next washed with saturated NaHCO_3_ (1 × 10 mL), water (1 × 10 mL), and brine (1 × 10 mL). The organic layer was dried with Na_2_SO_4_ and filtered, and the solvent was removed under reduced pressure. The condensed crude was purified by column chromato-graphy (40% EtOAc/hexane) to afford **16** (13.6 mg, *R*/*S* 1:2, 53% BRSM) as a yellow syrup in a mixture of C3 diastereomers.

#### Benzyl 4,5,7-tri-*O*-acetyl-2,3-dideoxy-β-d-*arabino*-2-enoseptanoside (**15**)

Synthesized using Method A. *R*_*f*_ 0.61 (40% EtOAc/Hex). Spectroscopic data for compound **15**: ^1^H NMR (400 MHz, CDCl_3_): δ ppm 7.41–7.24 (m, 5H), 5.88 (ddd, *J* = 12.1, 5.9, 1.5 Hz, 1H), 5.73 (dd, *J* = 12.1, 2.2 Hz, 1H), 5.38 (dd, *J* = 1.9, 1,9 Hz, 1H), 5.25 (dd, *J* = 5.9, 4.2 Hz, 1H), 5.21–5.04 (m, 1H), 4.87 (d, *J* = 12.0 Hz, 1H), 4.59 (d, *J* = 11.9 Hz, 1H), 4.35 (ddd, *J* = 9.4, 5.8, 2.9 Hz, 1H), 4.21 (dd, *J* = 11.9, 5.7 Hz, 1H), 4.15 (dd, *J* = 12.1, 2.9 Hz, 1H), 2.06 (s, 3H), 2.05 (s, 3H), 2.01 (s, 3H); ^13^C{^1^H} NMR (100 MHz, CDCl_3_): δ ppm 170.9, 169.9, 169.4, 137.5, 132.3, 128.7, 128.7, 127.8, 127.1, 126.8, 98.2, 73.2, 70.9, 69.8, 69.0, 65.5, 64.4, 21.0, 20.94, 20.88.; TOF HRMS (ESI) *m*/*z* calcd for C_20_H_25_O_8_ [M + H]^+^ 393.1549; found, 393.1519.

#### 2-(2,3,5-tri-*O*-acetyl-d-*arabino*-pentofuranosyl)-1,1-dibenzyloxyethane (**16**)

Synthesized using Method B. *R*_*f*_ 0.61 (40% EtOAc/Hex); major C3 isomer: ^1^H NMR (400 MHz, CDCl_3_): δ ppm 7.41–7.25 (m, 10H), 5.15 (dd, *J* = 3.5, 1.0 Hz, 1H), 4.95–4.90 (m, 2H), 4.68 (dd, *J* = 11.6, 9.1 Hz, 2H), 4.57 (dd, *J* = 11.7, 4.5 Hz, 2H), 4.33 (dd, *J* = 11.6, 4.9 Hz, 1H), 4.20–4.11 (m, 2H), 3.91 (ddd, *J* = 6.4, 4.8, 3.4 Hz, 1H), 2.08 (s, 3H), 2.08 (s, 3H), 2.07–2.05 (m, 2H), 2.10–2.00 (m, 3H); ^13^C{^1^H} NMR (100 MHz, CDCl3): δ 170.9, 169.8, 169.8, 138.1, 128.6, 128.0, 127.97, 127.94, 100.0, 81.4, 79.1, 77.6, 77.3, 68.3, 67.8, 63.9, 33.0, 20.97, 20.96, 20.8; minor C3 isomer: ^1^H NMR (400 MHz, CDCl_3_): δ 7.41–7.25 (m, 10H), 5.09 (m, 2H), 4.98 (dd, *J* = 7.4, 4.1 Hz, 1H), 4.68 (dd, *J* = 11.6, 9.1 Hz, 2H), 4.57 (dd, *J* = 11.7, 4.5 Hz, 2H), 4.29–4.25 (m, 2H), 4.20–4.11 (m, 2H), 2.08 (s, 3H), 2.08 (s, 3H), 2.07–2.05 (m, 2H), 2.06 (s, 3H). ^13^C{^1^H} NMR (100 MHz, CDCl_3_): δ ppm 170.9, 169.8, 169.8, 138.1, 128.6, 127.97, 127.94, 127.9, 99.9, 81.0, 80.7, 80.1, 78.9, 68.2, 67.7, 63.6, 36.9, 20.97, 20.95, 20.8; TOF HRMS (ESI) *m*/*z* calcd for C_27_H_32_NaO_9_ [M + Na]^+^ 523.1944; found, 523.1927.

#### 2-(2,3,5-tri-*O*-acetyl-d-*arabino*-pentofuranosyl)-acetaldehyde (**17**)

Synthesized using Method A using 69.3 mg (0.201 mmol) of **8**. Isolated as a colorless syrup (17.2 mg, 12% BRSM). *R*_*f*_ 0.24 (40% EtOAc/Hex); major isomer (*S*): 1H NMR (400 MHz, CDCl_3_): δ 9.79 (dd, *J* = 1.8, 1.0 Hz, 1H), 5.29 (dd, *J* = 3.8, 1.2 Hz, 1H), 4.99 (dd, *J* = 3.5, 1.2 Hz, 1H), 4.60–4.53 (m, 1H), 4.36 (dd, *J* = 11.6, 4.7 Hz, 1H), 4.17 (dd, *J* = 11.6, 6.4 Hz, 1H), 4.02 (ddd, *J* = 6.5, 4.7, 3.6 Hz, 1H), 2.89–2.81 (m, 1H), 2.78 (dd, *J* = 6.8, 1.9 Hz, 1H), 2.11 (s, 3H), 2.10 (s, 3H), 2.09 (s, 3H).; ^13^C{^1^H} NMR (100 MHz, CDCl3): δ 198.8, 170.8, 169.8, 169.7, 81.6, 78.8, 77.5, 75.5, 63.7, 42.9, 20.94, 20.90, 20.8. Minor isomer (*R*): ^1^H NMR (400 MHz, CDCl_3_): δ 9.77 (dd, *J* = 2.0, 1.5 Hz, 0H), 5.15 (dd, *J* = 2.7, 2.7 Hz, 1H), 5.05 (dd, *J* = 3.9, 2.4 Hz, 1H), 4.60–4.53 (m, 1H), 4.29–4.21 (m, 3H), 2.74 (dd, *J* = 5.8, 1.0 Hz, 1H), 2.70 (dd, *J* = 5.7, 1.0 Hz, 1H), 2.12 (s, 3H), 2.11 (s, 3H), 2.10 (s, 3H).; ^13^C{^1^H} NMR (100 MHz, CDCl_3_): δ 199.8, 170.8, 169.8, 169.6, 81.2, 80.8, 78.5, 78.3, 63.2, 46.6, 20.94, 20.90, 20.8; TOF HRMS (ESI) *m*/*z* calcd for C_13_H_19_O_8_ [M + H]^+^ 303.1080; found, 303.1083.

#### 3-(4,5,7-tri-*O*-acetyl-2,3-dideoxy-α-d-*arabino*-2-enoseptanosyl)-1-propene (**18**)

To a 10 mL round-bottom flask was added oxepine **8** (23.9 mg, 0.0694 mmol), and the sample was dried by azeotropic distillation from toluene (3 × 2 mL). The contents were then dissolved in dry DCM (2 mL) and cooled to −45 °C. Allyltrimethylsilane (12.0 μL, 0.0755 mmol) was added to the solution, followed by the slow addition of BF_3_·OEt_2_ (8.6 μL, 0.067 mmol). The mixture was immediately switched to a bath at −20 °C and stirred at that temperature for 1 h. Then, the reaction was quenched with aq. NaHCO_3_ (1 mL). The mixture was then diluted with DCM (10 mL) which was sequentially washed with saturated NaHCO_3_ (1 × 10 mL), water (1 × 10 mL), and brine (1 × 10 mL). The organic layer was dried with Na_2_SO_4_ and filtered, and the solvent was removed under reduced pressure to give compound **18** (17.5 mg, 77%) as a colorless oil. R_f_ 0.63 (40% EtOAc/hexanes); ^1^H NMR (400 MHz, acetone-*d*_6_): δ ppm 5.87 (dddd, *J* = 17.2, 13.9, 10.2, 6.9 Hz, 1H), 5.78 (ddd, *J* = 11.7, 9.2, 2.6 Hz, 1H), 5.73 (ddd, *J* = 11.8, 2.2, 2.2 Hz, 1H), 5.51 (ddd, *J* = 11.8, 2.5, 2.5 Hz, 1H), 5.12 (dddd, *J* = 17.2, 2.0, 1.5, 1.5 Hz, 1H), 5.10–5.02 (m, 2H), 4.49 (dddd, *J* = 8.0, 4.9, 2.4, 2.4 Hz, 1H), 4.42 (ddd *J* = 8.8, 8.8, 1.9 Hz, 1H), 4.11–4.03 (m, 2H), 2.32 (dddd, *J* = 12.0, 5.6, 2.6, 1.2 Hz, 2H), 2.03 (s, 3H), 2.02 (s, 6H); ^13^C{^1^H} NMR (100 MHz): δ ppm 170.7, 170.1, 170.0, 135.6, 134.8, 127.9, 117.5, 75.3, 73.2, 72.4, 71.3, 62.8, 40.2, 20.8, 20.7, 20.7; TOF HRMS (ESI) *m*/*z* calcd for C_16_H_26_NO_7_ [M + NH_4_]^+^ 344.1709; found, 344.1695.

## Data Availability

The data underlying this study are available in the published article and its Supporting Information.

## Supporting Information Available

Mass spectra, computed energetics, 3D structures, and NMR spectra (PDF)
*xyz* coordinates (XYZ)

## Supplementary Material

jo3c00079_si_001.pdf

jo3c00079_si_002.xyz
