# Supplementary material for: Characterization and Fate of a Septanosyl Ferrier Cation in the Gas and Solution Phases
Source: J Org Chem. 2023 Apr 24;88(9):5543–53. doi: 10.1021/acs.joc.3c00079 (PMC10167658; doi:10.1021/acs.joc.3c00079)

Supporting Information

# Characterization and fate of a septanosyl Ferrier cation in the gas and solution phases

Kim Greis[1,2†], Caleb Griesbach[3†], Carla Kirschbaum[1,2], Gerard Meijer[1], Gert von Helden[1], Kevin Pagel[1,2*], Mark W. Peczuh[3*]

[1] Fritz Haber Institute of the Max Planck Society, 14195 Berlin, Germany
[2] Freie Universität Berlin, Institute of Chemistry and Biochemistry, 14195 Berlin, Germany
[3] Department of Chemistry, University of Connecticut, 55 N. Eagleville Road, U3060, Storrs, CT 06269 USA
† Equal contribution

Correspondence to: Prof. Dr. Mark W. Peczuh
mark.peczuh@uconn.edu

Prof. Dr. Kevin Pagel
kevin.pagel@fu-berlin.de

## Contents

Figure S1. Electrospray ionization mass spectra of glucose- (top) and mannose-like (bottom) acetylated oxepines in positive ion mode. The main signal (*m/z* = 367) corresponds to the sodiated oxepines [M + $Na^+$]. Cleavage of an acetoxy group leads to $[M – OAc]^+$ ions (*m/z* = 285). Furthermore, sodium bound dimers of the precursor $[2M + Na]^+$ are formed (*m/z* = 711)........................................................ S3

Table S1. List of structures of the septanosyl Ferrier cation generated after fragmentation of 8 or 10. Each structure was optimized at the PBE0+D3/6-311+G(d,p) level of theory. Only structures within 20 kJ $mol^{-1}$ of the lowest-energy structure of the respective structural motif are represented. Relative energies with and without zero-point vibrational energy (ΔE and ΔE+ZPVE) as well as relative free energies (ΔF) at 90 K (the temperature of the ion trap) are indicated in kJ $mol^{-1}$. The vibrational spectra of the structures labelled with a roman number are shown in the manuscript. .................................... S4

Table S2. DLPNO-CCSD(T)/Def2-TZVPP single-point energies of selected structures of septanosyl Ferrier cation optimized at the PBE0+D3/6-311+G(d,p) level of theory. The structures whose spectra are shown in the manuscript were selected for calculation of high-level single-point energies. Zero-point vibrational energy and free energy corrections are derived from the PBE0 calculations. All energies are indicated in kJ $mol^{-1}$. ........................................................................................................................ S8

Figure S2. The experimental infrared spectrum (gray) of the septanosyl Ferrier cation $[M – OAc]^+$ compared to computed spectra (red, inverted traces) of structures exhibiting rearrangement by attack of the (VII') C5-, (VIII') C7-, (IX') C4-acetyl group at the C6 position leading to ring opening. The structures are diastereomeric to the ones shown in Figure 5 and generally less stable (except for the C4_rearranged diastereomer) and their harmonic frequencies generally match less well to the experiment. R/S/R is referring to the stereoconfiguration at C4/C5/C6. The relative free energy at 90 K as well as schematic depictions of each structure are indicated. ......................................................... S9

Figure S3. Energy diagrams for (a) rearrangement of oxocarbenium- or C4_C3_NGP-structures to the C5- and C7-rearranged structures (RSS diastereomer), respectively, and (b) reaction of C5/C7-rearranged structure (RSS diastereomer) to C7/C5-rearranged structure (RSR diastereomer). All energies are computed at the DLPNO-CCSD(T)/Def2-TZVPP level of theory with ZPVE and free-energy correction at the PBE0+D3/6-311+G(d,p) level of theory. All energies are relative to those of C4_C3_NGP/conf_7 (I). ........................................................................................................ S10

Figure S4. Selected reoptimized 3D-geometries of low-energy structures of (I) C4_C3_NGP, (II) C5_C3_LRP, (III) C7_C1_LRP, (IV) C5_C1_LRP, (V) C4_C1_LRP, and (VI) C7_C3_LRP septanosyl Ferrier cations. Hydrogen atoms are omitted for clarity. ........ S11

Figure S5. Selected reoptimized 3D-geometries of low-energy structures of (VII) C5_rearranged, (VIII) C7_rearranged, (IX) C4_rearranged, (X) oxocarbenium, and (XI) oxocarbenium "sandwich" septanosyl Ferrier cations. All rearranged structures are shown as R/S/S (C4/C5/C6) diastereomers. Hydrogen atoms are omitted for clarity. ........ S12

Figure S6. 3D structures of C4_C3_NGP (I) and the oxocarbenium "sandwich" (XI) with nucleophilic path of attack. ........ S13

Table S3. Tabulated NMR data for compounds 15, 16, and 18 ........ S14

Figure S7. NOESY NMR of the 15/16 mixture showing H1-H6 NOE interaction. ........ S15

Figure S8. Zoomed version of the HSQC with assignments. ........ S16

Figure S9. COSY view of nonaromatic protons with correlations of the major isomer. ........ S17

Figure S10. TOCSY experiment showing H4 and H5 cross peak. ........ S18

Table S4. Tabulated chemical shifts of the 16. ........ S19

Figure S11. HMBC spectrum showing acetate cross peaks (red circles), benzyl-acetal cross peak (green circle), and C6-H3 cross peak. ........ S19

Figure S12. Zoomed version of the NOESY spectrum. ........ S20

Table S5. Common abbreviations used in the main text. ........ S21

$^{1}$H NMR of compound 8 ........ S22

$^{1}$H NMR of compound 10 ........ S23

NMR spectra used to characterize compound 15 ........ S24

NMR characterization data of 16 – wet Ferrier product ........ S30

NMR characterization data of 17 ........ S36

NMR spectra used to characterize compound 18 ........ S40

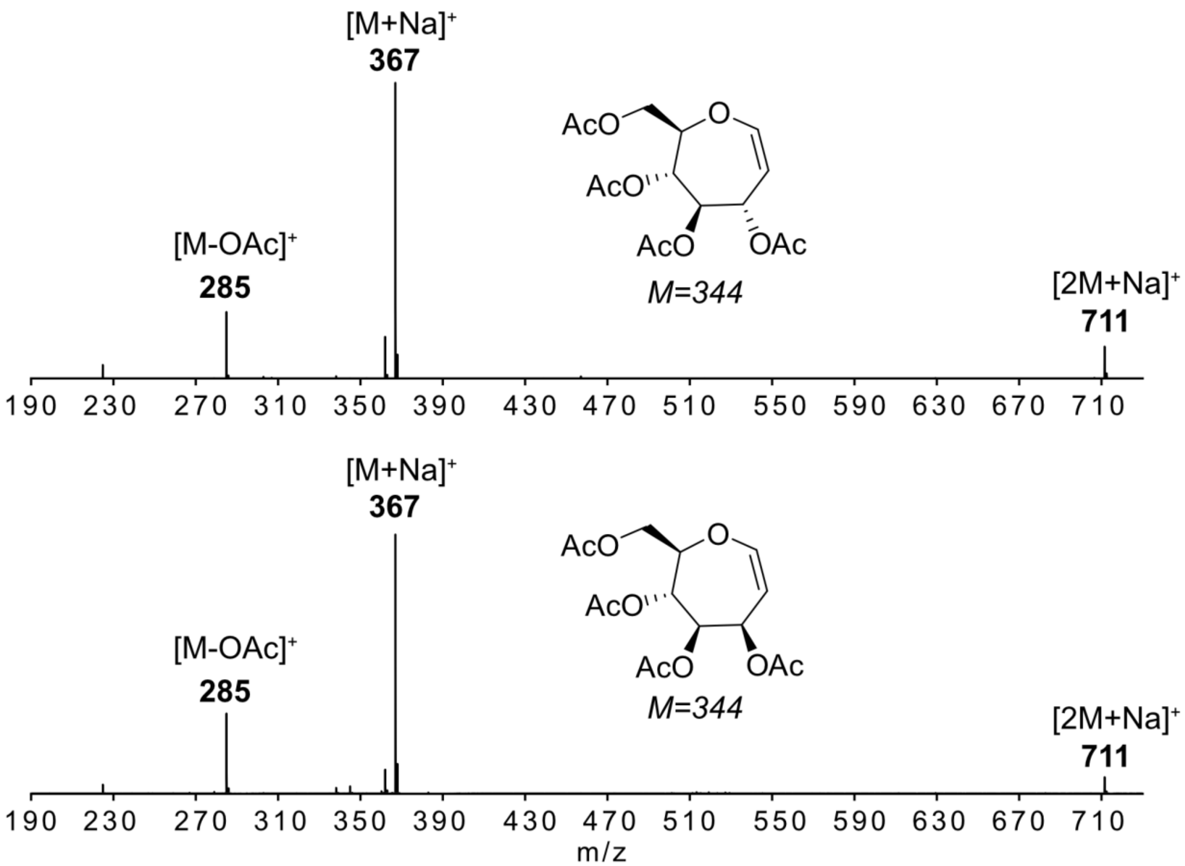


**Figure S1.** Electrospray ionization mass spectra of glucose- (top) and mannose-like (bottom) acetylated oxepines in positive ion mode. The main signal (*m/z* = 367) corresponds to the sodiated oxepines [M + Na$^+$]. Cleavage of an acetoxy group leads to [M – OAc]$^+$ ions (*m/z* = 285). Furthermore, sodium bound dimers of the precursor [2M + Na]$^+$ are formed (*m/z* = 711).

**Table S1.** List of structures of the septanosyl Ferrier cation generated after fragmentation of **8** or **10**. Each structure was optimized at the PBE0+D3/6-311+G(d,p) level of theory. Only structures within 20 kJ mol$^{-1}$ of the lowest-energy structure of the respective structural motif are represented. Relative energies with and without zero-point vibrational energy (ΔE and ΔE+ZPVE) as well as relative free energies (ΔF) at 90 K (the temperature of the ion trap) are indicated in kJ mol$^{-1}$. The vibrational spectra of the structures labelled with a roman number are shown in the manuscript.

| ID | ΔE(PBE0) [kJ mol$^{-1}$] | ΔE+ZPVE(PBE0) [kJ mol$^{-1}$] | ΔF(PBE0, 90K) [kJ mol$^{-1}$] |
|---|---|---|---|
| C4_C3_NGP/conf_0[(a)] | 8.71 | 9.91 | 10.19 |
| C4_C3_NGP/conf_1[(a)] | 8.35 | 9.91 | 10.77 |
| C4_C3_NGP/conf_2[(a)] | 5.49 | 7.76 | 8.25 |
| C4_C3_NGP/conf_3[(a)] | 2.16 | 2.17 | 3.47 |
| C4_C3_NGP/conf_4[(a)] | 9.41 | 10.95 | 11.35 |
| C4_C3_NGP/conf_5[(a)] | 5.08 | 5.10 | 5.15 |
| C4_C3_NGP/conf_6[(a)] | 13.63 | 15.82 | 15.73 |
| C4_C3_NGP/conf_7 (**I**)[(a)] | 0.00 | 0.00 | 0.00 |
| C4_C3_NGP/conf_8[(a)] | 4.78 | 5.65 | 6.34 |
| C4_C3_NGP/conf_9[(a)] | 4.74 | 6.76 | 7.09 |
| C4_C3_NGP/conf_10[(a)] | 4.42 | 5.15 | 4.48 |
| C4_C3_NGP/conf_11[(a)] | 14.62 | 16.70 | 16.12 |
| C4_C3_NGP/conf_12[(a)] | 3.93 | 4.05 | 3.87 |
| C4_C3_NGP/conf_13[(a)] | 15.76 | 15.79 | 17.02 |
| C4_C3_NGP/conf_14[(a)] | 10.49 | 11.23 | 10.16 |
| C4_C3_NGP/conf_15[(a)] | 6.24 | 6.14 | 5.70 |
| C4_C3_NGP/conf_16[(a)] | 19.45 | 19.20 | 19.62 |
| C4_C3_NGP/conf_17[(a)] | 10.94 | 10.14 | 10.12 |
| C4_C3_NGP/conf_18[(a)] | 12.94 | 12.65 | 11.72 |
| C4_C1_LRP/conf_0[(a)] | 42.39 | 43.29 | 43.75 |
| C4_C1_LRP/conf_1[(a)] | 44.74 | 45.04 | 44.77 |
| C4_C1_LRP/conf_2[(a)] | 42.25 | 42.75 | 42.82 |
| C4_C1_LRP/conf_3[(a)] | 34.45 | 34.71 | 35.60 |
| C4_C1_LRP/conf_4[(a)] | 43.03 | 42.92 | 42.90 |
| C4_C1_LRP/conf_5[(a)] | 43.17 | 42.88 | 42.11 |
| C4_C1_LRP/conf_6[(a)] | 47.31 | 47.55 | 48.05 |
| C4_C1_LRP/conf_7 (**V**)[(a)] | 36.44 | 35.41 | 34.70 |
| C4_C1_LRP/conf_8[(a)] | 42.42 | 42.48 | 43.18 |
| C4_C1_LRP/conf_9[(a)] | 39.40 | 38.89 | 39.28 |
| C4_C1_LRP/conf_10[(a)] | 51.24 | 51.32 | 52.15 |
| C4_C1_LRP/conf_11[(a)] | 51.02 | 50.30 | 49.70 |
| C4_C1_LRP/conf_12[(a)] | 46.64 | 46.18 | 46.14 |
| C4_C1_LRP/conf_13[(a)] | 49.22 | 47.88 | 46.71 |
| C4_C1_LRP/conf_14[(a)] | 45.09 | 44.28 | 44.04 |
| C4_C1_LRP/conf_15[(a)] | 45.28 | 44.36 | 43.95 |
| C5_C3_LRP/conf_0[(a)] | 23.92 | 24.92 | 25.75 |
| C5_C3_LRP/conf_1[(a)] | 21.76 | 23.24 | 23.95 |
| C5_C3_LRP/conf_2[(a)] | 27.07 | 28.05 | 28.88 |
| C5_C3_LRP/conf_3 (**II**)[(a)] | 19.04 | 20.65 | 21.72 |
| C5_C3_LRP/conf_4[(a)] | 24.97 | 26.47 | 27.25 |
| C5_C3_LRP/conf_5[(a)] | 21.53 | 22.05 | 22.05 |
| C5_C3_LRP/conf_6[(a)] | 22.45 | 22.47 | 23.87 |

**Table S1 continued.** List of structures of the septanosyl Ferrier cation generated after fragmentation of **8** or **10**. Each structure was optimized at the PBE0+D3/6-311+G(d,p) level of theory. Only structures within 20 kJ mol$^{-1}$ of the lowest-energy structure of the respective structural motif are represented. Relative energies with and without zero-point vibrational energy (ΔE and ΔE+ZPVE) as well as relative free energies (ΔF) at 90 K (the temperature of the ion trap) are indicated in kJ mol$^{-1}$. The vibrational spectra of the structures labelled with a roman number are shown in the manuscript.

| ID | ΔE(PBE0) [kJ mol$^{-1}$] | ΔE+ZPVE(PBE0) [kJ mol$^{-1}$] | ΔF(PBE0, 90K) [kJ mol$^{-1}$] |
|---|---|---|---|
| C5_C3_LRP/conf_7[(a)] | 22.66 | 23.48 | 23.95 |
| C5_C1_LRP/conf_0[(a)] | 31.45 | 31.64 | 32.25 |
| C5_C1_LRP/conf_1 (**IV**)[(a)] | 31.57 | 31.67 | 31.97 |
| C5_C1_LRP/conf_2[(a)] | 37.01 | 36.36 | 35.70 |
| C5_C1_LRP/conf_3[(a)] | 37.21 | 37.62 | 37.55 |
| C5_C1_LRP/conf_4[(a)] | 42.52 | 41.69 | 41.81 |
| C5_C1_LRP/conf_5[(a)] | 42.43 | 41.66 | 41.86 |
| C5_C1_LRP/conf_6[(a)] | 40.61 | 40.14 | 39.65 |
| C5_C1_LRP/conf_7[(a)] | 40.55 | 40.16 | 39.67 |
| C7_C3_LRP/conf_0 (**VI**)[(a)] | 52.95 | 55.68 | 58.44 |
| C7_C1_LRP/conf_0[(a)] | 42.19 | 40.81 | 40.35 |
| C7_C1_LRP/conf_1[(a)] | 46.80 | 44.56 | 44.32 |
| C7_C1_LRP/conf_2[(a)] | 27.62 | 26.83 | 26.38 |
| C7_C1_LRP/conf_3[(a)] | 42.29 | 39.72 | 38.18 |
| C7_C1_LRP/conf_4[(a)] | 30.42 | 29.28 | 28.98 |
| C7_C1_LRP/conf_5[(a)] | 30.03 | 29.08 | 28.40 |
| C7_C1_LRP/conf_6[(a)] | 28.83 | 26.86 | 26.04 |
| C7_C1_LRP/conf_7 (**III**)[(a)] | 29.27 | 27.03 | 25.60 |
| C7_C1_LRP/conf_8[(a)] | 40.40 | 37.56 | 37.42 |
| oxocarbenium/conf_0[(c)] | 26.77 | 22.27 | 20.72 |
| oxocarbenium/conf_1 (**X**)[(c)] | 26.41 | 19.91 | 16.28 |
| oxocarbenium/conf_2[(c)] | 23.59 | 18.83 | 16.97 |
| oxocarbenium/conf_3 (**XI**)[(c)] | 25.07 | 20.95 | 20.19 |
| oxocarbenium/conf_4[(c)] | 30.13 | 24.66 | 22.74 |
| oxocarbenium/conf_5[(b)] | 27.28 | 21.07 | 17.65 |
| oxocarbenium/conf_6[(b)] | 41.77 | 35.16 | 32.46 |
| C4_rearranged_RSS/conf_0 (**IX**)[(b)] | 15.20 | 6.15 | 3.76 |
| C4_rearranged_RSS/conf_1[(b)] | 19.32 | 9.58 | 5.25 |
| C4_rearranged_RSS/conf_2[(b)] | 18.27 | 9.23 | 6.25 |
| C4_rearranged_RSS/conf_3[(b)] | 22.05 | 11.70 | 6.63 |
| C4_rearranged_RSS/conf_4[(b)] | 18.45 | 9.33 | 6.17 |
| C4_rearranged_RSS/conf_5[(b)] | 26.06 | 16.63 | 13.47 |
| C4_rearranged_RSS/conf_6[(b)] | 27.84 | 18.71 | 15.61 |
| C5_rearranged_RSS/conf_0[(c)] | -0.97 | -8.99 | -11.76 |
| C5_rearranged_RSS/conf_1[(c)] | 1.89 | -5.48 | -8.10 |
| C5_rearranged_RSS/conf_2[(c)] | -2.77 | -11.11 | -14.16 |
| C5_rearranged_RSS/conf_3[(c)] | 7.34 | -0.49 | -3.35 |
| C5_rearranged_RSS/conf_4[(c)] | 7.18 | 0.66 | -0.84 |
| C5_rearranged_RSS/conf_5[(c)] | 10.01 | 1.60 | -1.94 |
| C5_rearranged_RSS/conf_6[(c)] | 9.85 | 2.32 | -1.05 |
| C5_rearranged_RSS/conf_7[(c)] | 11.13 | 3.82 | 1.48 |
| C5_rearranged_RSS/conf_8[(c)] | 8.82 | 2.38 | 0.48 |
| C5_rearranged_RSS/conf_9[(c)] | 12.71 | 3.68 | -0.75 |

**Table S1 continued.** List of structures of the septanosyl Ferrier cation generated after fragmentation of **8** or **10**. Each structure was optimized at the PBE0+D3/6-311+G(d,p) level of theory. Only structures within 20 kJ mol$^{-1}$ of the lowest-energy structure of the respective structural motif are represented. Relative energies with and without zero-point vibrational energy (ΔE and ΔE+ZPVE) as well as relative free energies (ΔF) at 90 K (the temperature of the ion trap) are indicated in kJ mol$^{-1}$. The vibrational spectra of the structures labelled with a roman number are shown in the manuscript.

| ID | ΔE(PBE0) [kJ mol$^{-1}$] | ΔE+ZPVE(PBE0) [kJ mol$^{-1}$] | ΔF(PBE0, 90K) [kJ mol$^{-1}$] |
|---|---|---|---|
| C5_rearranged_RSS/conf_10[(c)] | 16.18 | 7.36 | 4.50 |
| C5_rearranged_RSS/conf_11[(c)] | 12.04 | 4.20 | 1.03 |
| C5_rearranged_RSS/conf_12[(c)] | -4.07 | -11.10 | -13.22 |
| C5_rearranged_RSS/conf_13[(c)] | 9.15 | 0.28 | -3.94 |
| C5_rearranged_RSS/conf_14[(c)] | -0.24 | -6.67 | -8.38 |
| C5_rearranged_RSS/conf_15[(c)] | 9.94 | 1.19 | -2.24 |
| C5_rearranged_RSS/conf_16[(c)] | 0.75 | -7.24 | -10.12 |
| C5_rearranged_RSS/conf_17[(c)] | 14.25 | 6.78 | 3.52 |
| C5_rearranged_RSS/conf_18[(c)] | 10.51 | 2.02 | -2.21 |
| C5_rearranged_RSS/conf_19[(c)] | 4.62 | -1.72 | -3.08 |
| C5_rearranged_RSS/conf_20[(c)] | 14.33 | 5.81 | 1.50 |
| C5_rearranged_RSS/conf_21[(c)] | -4.65 | -11.33 | -13.28 |
| C5_rearranged_RSS/conf_22[(c)] | 6.97 | -0.96 | -4.19 |
| C5_rearranged_RSS/conf_23[(c)] | 11.76 | 1.83 | -3.13 |
| C5_rearranged_RSS/conf_24[(c)] | 17.65 | 7.61 | 1.24 |
| C5_rearranged_RSS/conf_25[(c)] | 13.46 | 5.42 | 1.24 |
| C5_rearranged_RSS/conf_26[(c)] | 9.28 | 0.61 | -3.40 |
| C5_rearranged_RSS/conf_27[(a)] | -7.00 | -13.57 | -15.26 |
| C5_rearranged_RSS/conf_28[(a)] | 2.18 | -5.26 | -8.03 |
| C5_rearranged_RSS/conf_29[(a)] | -0.97 | -8.99 | -11.75 |
| C5_rearranged_RSS/conf_30 (**VII**)[(a)] | -8.06 | -14.07 | -15.41 |
| C5_rearranged_RSS/conf_31[(b)] | 9.85 | 1.11 | -2.22 |
| C5_rearranged_RSS/conf_32[(b)] | 7.18 | 0.65 | -0.86 |
| C5_rearranged_RSS/conf_33[(b)] | 8.82 | 2.38 | 0.50 |
| C7_rearranged_RSS/conf_0[(c)] | 8.47 | 1.38 | -1.19 |
| C7_rearranged_RSS/conf_1 (**VIII**)[(c)] | -5.22 | -12.42 | -15.11 |
| C7_rearranged_RSS/conf_2[(c)] | 11.73 | 4.27 | 1.36 |
| C7_rearranged_RSS/conf_3[(c)] | 10.60 | 4.93 | 3.26 |
| C7_rearranged_RSS/conf_4[(c)] | 3.14 | -2.81 | -3.92 |
| C7_rearranged_RSS/conf_5[(c)] | 4.76 | -0.95 | -2.69 |
| C7_rearranged_RSS/conf_6[(c)] | 10.95 | 3.55 | 1.01 |
| C4_rearranged/conf_0[(c)] | 6.13 | -0.55 | -2.40 |
| C4_rearranged_RSR/conf_1 (**IX’**)[(c)] | 4.69 | -2.94 | -5.06 |
| C4_rearranged_RSR/conf_2[(c)] | 21.28 | 12.66 | 8.74 |
| C4_rearranged_RSR/conf_3[(c)] | 16.83 | 6.73 | 1.97 |
| C4_rearranged_RSR/conf_4[(c)] | 5.64 | -1.50 | -4.49 |
| C4_rearranged_RSR/conf_5[(c)] | 9.08 | 1.93 | -0.83 |
| C4_rearranged_RSR/conf_6[(c)] | 21.20 | 13.05 | 9.59 |
| C4_rearranged_RSR/conf_7[(c)] | 20.85 | 13.74 | 11.95 |
| C4_rearranged_RSR/conf_8[(c)] | 15.74 | 8.78 | 7.16 |
| C4_rearranged_RSR/conf_9[(c)] | 24.27 | 14.33 | 9.85 |
| C4_rearranged_RSR/conf_10[(c)] | 11.95 | 3.88 | 1.02 |
| C4_rearranged_RSR/conf_11[(c)] | 21.27 | 13.07 | 9.72 |

**Table S1 continued.** List of structures of the septanosyl Ferrier cation generated after fragmentation of **8** or **10**. Each structure was optimized at the PBE0+D3/6-311+G(d,p) level of theory. Only structures within 20 kJ mol$^{-1}$ of the lowest-energy structure of the respective structural motif are represented. Relative energies with and without zero-point vibrational energy (ΔE and ΔE+ZPVE) as well as relative free energies (ΔF) at 90 K (the temperature of the ion trap) are indicated in kJ mol$^{-1}$. The vibrational spectra of the structures labelled with a roman number are shown in the manuscript.

| ID | ΔE(PBE0) [kJ mol$^{-1}$] | ΔE+ZPVE(PBE0) [kJ mol$^{-1}$] | ΔF(PBE0, 90K) [kJ mol$^{-1}$] |
|---|---|---|---|
| C4_rearranged_RSR/conf_12[(c)] | 23.45 | 14.16 | 9.96 |
| C4_rearranged_RSR/conf_13[(c)] | 24.63 | 15.72 | 10.98 |
| C4_rearranged_RSR/conf_14[(c)] | 18.51 | 11.36 | 8.95 |
| C4_rearranged_RSR conf_15[(b)] | 20.98 | 12.15 | 8.81 |
| C4_rearranged_RSR/conf_16[(b)] | 14.77 | 5.32 | 1.24 |
| C4_rearranged_RSR/conf_17[(b)] | 14.96 | 5.38 | 1.07 |
| C5_rearranged_RSR/conf_0[(b)] | 26.20 | 17.65 | 14.21 |
| C5_rearranged_RSR/conf_1[(b)] | 26.10 | 17.64 | 14.35 |
| C5_rearranged_RSR/conf_2[(b)] | 20.94 | 12.55 | 9.10 |
| C5_rearranged_RSR/conf_3[(b)] | 25.56 | 18.50 | 16.32 |
| C5_rearranged_RSR/conf_4[(b)] | 25.38 | 15.85 | 11.15 |
| C5_rearranged_RSR/conf_5 (**VII’**) [(b)] | 17.01 | 8.98 | 6.22 |
| C5_rearranged_RSR/conf_6[(b)] | 22.23 | 14.98 | 13.05 |
| C5_rearranged_RSR/conf_7[(b)] | 21.71 | 14.64 | 12.49 |
| C5_rearranged_RSR/conf_8[(b)] | 26.21 | 19.31 | 17.49 |
| C5_rearranged_RSR/conf_9[(b)] | 23.10 | 16.05 | 14.03 |
| C5_rearranged_RSR/conf_10[(b)] | 23.41 | 15.20 | 11.27 |
| C7_rearranged_RSR/conf_0[(b)] | 7.46 | -0.13 | -4.01 |
| C7_rearranged_RSR/conf_1[(b)] | 12.45 | 4.37 | 0.54 |
| C7_rearranged_RSR/conf_2[(b)] | 2.99 | -4.21 | -7.60 |
| C7_rearranged_RSR/conf_3[(b)] | 5.21 | -2.26 | -5.16 |
| C7_rearranged_RSR/conf_4 (**VIII’**)[(b)] | 0.52 | -6.93 | -10.38 |
| C7_rearranged_RSR/conf_5[(b)] | 20.36 | 12.77 | 10.20 |
| C7_rearranged_RSR/conf_6[(b)] | 11.82 | 4.71 | 2.39 |
| C5_rearrangement/IRCb[(d)] | 27.81 | 26.83 | 25.98 |
| C5_rearrangement/TS[(d)] | 106.87 | 93.47 | 92.11 |
| C5_rearrangement/IRCf[(d)] | 20.31 | 11.67 | 7.46 |
| C7_rearrangement/IRCb[(d)] | 23.39 | 22.58 | 21.86 |
| C7_rearrangement/TS[(d)] | 109.92 | 98.07 | 96.54 |
| C7_rearrangement/IRCf[(d)] | 20.56 | 12.77 | 10.52 |
| C5_RSS_C7_RSR_swap/IRCb[(d)] | 12.71 | 3.66 | -0.80 |
| C5_RSS_C7_RSR _swap/TS[(d)] | 69.49 | 56.90 | 54.56 |
| C5_RSS_C7_RSR_swap/IRCf[(d)] | 11.10 | 3.51 | 1.36 |
| C7_RSS_C5_RSR_swap/IRCb[(d)] | 3.14 | -2.82 | -3.93 |
| C7_RSS_C5_RSR_swap/TS[(d)] | 70.55 | 58.28 | 55.37 |
| C7_RSS_C5_RSR_swap/IRCf[(d)] | 20.94 | 12.55 | 9.10 |

The structures were obtained after sampling with (a) CREST-GFN2, (b) CREST-GFNFF, or (c) Maestro. (d) The labelled structures were used to construct the energy diagrams in Figure S3. IRC: intrinsic reaction coordinate, TS: transition state.

**Table S2.** DLPNO-CCSD(T)/Def2-TZVPP single-point energies of selected structures of septanosyl Ferrier cation optimized at the PBE0+D3/6-311+G(d,p) level of theory. The structures whose spectra are shown in the manuscript were selected for calculation of high-level single-point energies. Zero-point vibrational energy and free energy corrections are derived from the PBE0 calculations. All energies are indicated in kJ $mol^{-1}$.

| ID | ΔE(CCSD(T)) [kJ $mol^{-1}$] | ΔE+ZPVE(CCSD(T)) [kJ $mol^{-1}$] | ΔF(CCSD(T), 90K) [kJ $mol^{-1}$] |
|---|---|---|---|
| C4_C3_NGP/conf_7 (**I**) | 0.00 | 0.00 | 0.00 |
| C5_C3_LRP/conf_3 (**II**) | 12.69 | 14.30 | 15.36 |
| C7_C1_LRP/conf_7 (**III**) | 24.61 | 22.37 | 20.93 |
| C5_C1_LRP/conf_1 (**IV**) | 27.42 | 27.52 | 27.83 |
| C4_C1_LRP/conf_7 (**V**) | 30.99 | 29.96 | 29.25 |
| C7_C3_LRP/conf_0 (**VI**) | 56.03 | 58.76 | 61.52 |
| oxocarbenium/conf_1 (**X**) | 25.38 | 18.88 | 15.24 |
| oxocarbenium/conf_3 (**XI**) | 31.00 | 26.88 | 26.12 |
| C4_rearranged_RSS/conf_2 (**IX**) | 2.07 | -6.99 | -9.38 |
| C5_rearranged_RSS/conf_30 (**VII**) | -25.34 | -31.36 | -32.69 |
| C7_rearranged_RSS/conf_1 (**VIII**) | -22.33 | -29.52 | -32.22 |
| C4_rearranged_RSR/conf_1 (**IX’**) | -9.01 | -16.64 | -18.77 |
| C5_rearranged_RSR/conf_5 (**VII’**) | 1.10 | -6.93 | -9.69 |
| C7_rearranged_RSR/conf_4 (**VIII’**) | -15.05 | -22.50 | -25.95 |
| C5_rearrangement/IRCb[(a)] | 27.34 | 26.36 | 25.51 |
| C5_rearrangement/TS[(a)] | 108.38 | 94.98 | 93.62 |
| C5_rearrangement/IRCf[(a)] | 5.02 | -3.62 | -7.82 |
| C7_rearrangement/IRCb[(a)] | 11.86 | 11.05 | 10.33 |
| C7_rearrangement/TS[(a)] | 107.19 | 95.34 | 93.81 |
| C7_rearrangement/IRCf[(a)] | 3.25 | -4.54 | -6.78 |
| C5_RSS_C7_RSR_swap/IRCb[(a)] | -3.94 | -12.99 | -17.45 |
| C5_RSS_C7_RSR _swap/TS[(a)] | 58.43 | 45.84 | 43.50 |
| C5_RSS_C7_RSR_swap/IRCf[(a)] | -4.97 | -12.55 | -14.71 |
| C7_RSS_C5_RSR_swap/IRCb[(a)] | -10.18 | -16.14 | -17.25 |
| C7_RSS_C5_RSR_swap/TS[(a)] | 60.37 | 48.11 | 45.19 |
| C7_RSS_C5_RSR_swap/IRCf[(a)] | 3.82 | -4.57 | -8.02 |

(a) The labelled structures were used to construct the energy diagrams in Figure S3. IRC: intrinsic reaction coordinate, TS: transition state.

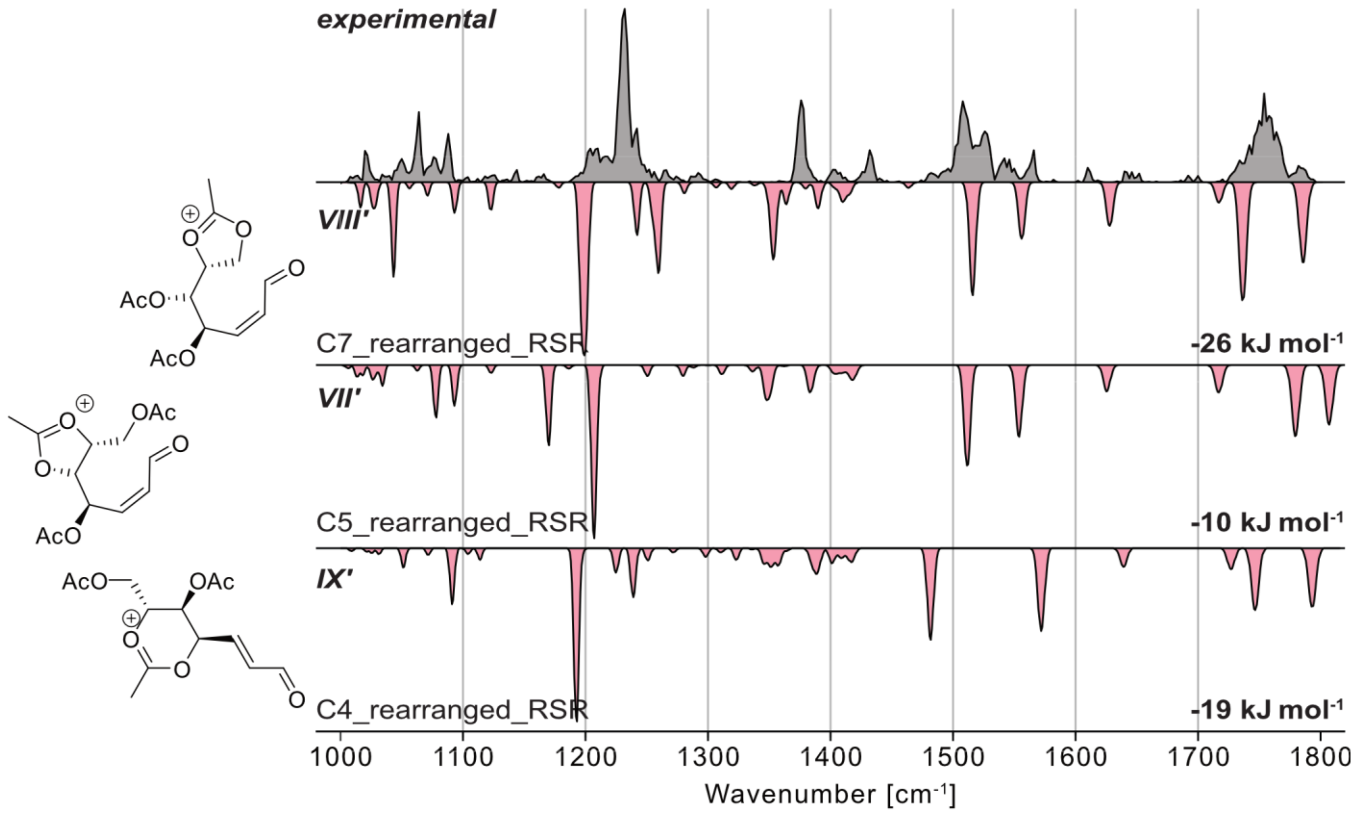


**Figure S2.** The experimental infrared spectrum (gray) of the septanosyl Ferrier cation [M – OAc]$^+$ compared to computed spectra (red, inverted traces) of structures exhibiting rearrangement by attack of the (**VII'**) C5-, (**VIII'**) C7-, (**IX'**) C4-acetyl group at the C6 position leading to ring opening. The structures are diastereomeric to the ones shown in Figure 5 and generally less stable (except for the C4_rearranged diastereomer) and their harmonic frequencies generally match less well to the experiment. R/S/R is referring to the stereoconfiguration at C4/C5/C6. The relative free energy at 90 K as well as schematic depictions of each structure are indicated.

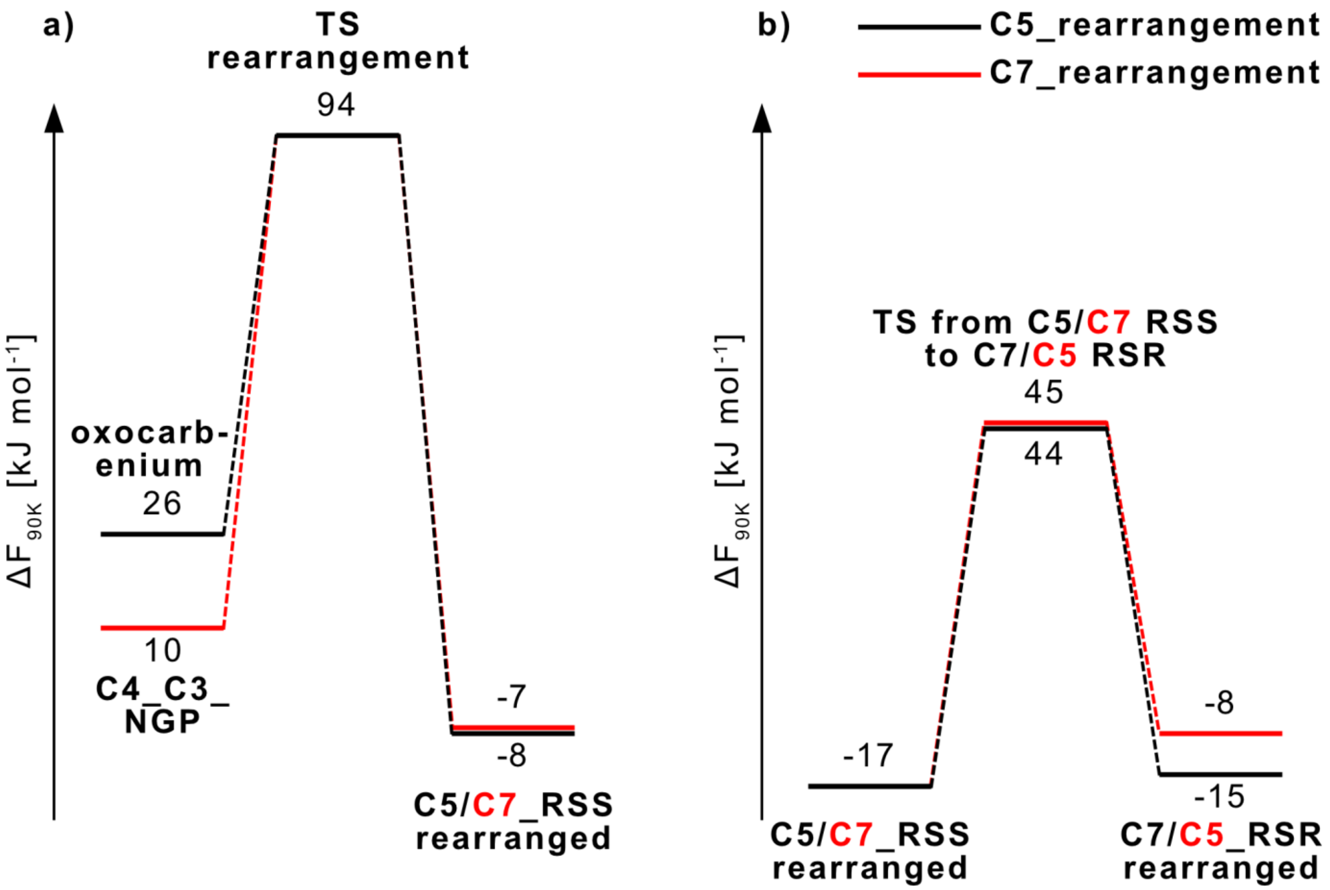


**Figure S3.** Energy diagrams for (a) rearrangement of oxocarbenium- or C4_C3_NGP-structures to the C5- and C7-rearranged structures (RSS diastereomer), respectively, and (b) reaction of C5/C7-rearranged structure (RSS diastereomer) to C7/C5-rearranged structure (RSR diastereomer). All energies are computed at the DLPNO-CCSD(T)/Def2-TZVPP level of theory with ZPVE and free-energy correction at the PBE0+D3/6-311+G(d,p) level of theory. All energies are relative to those of C4_C3_NGP/conf_7 (**I**).

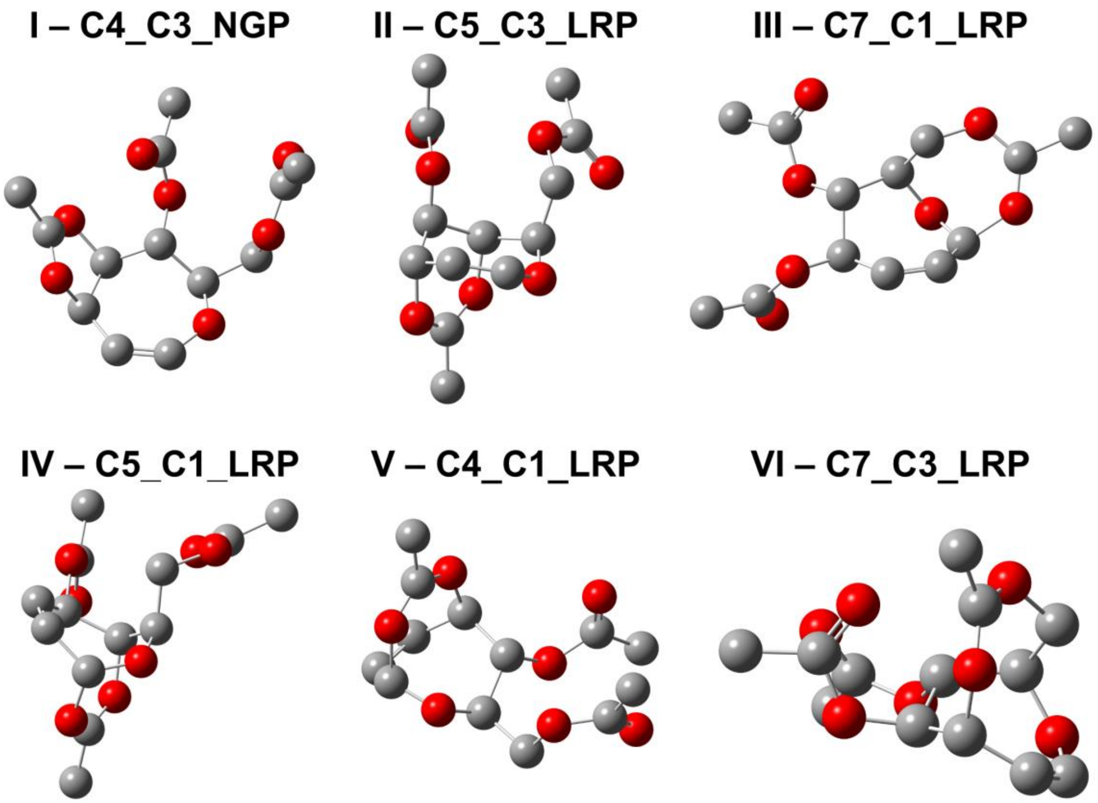


**Figure S4.** Selected reoptimized 3D-geometries of low-energy structures of (**I**) C4_C3_NGP, (**II**) C5_C3_LRP, (**III**) C7_C1_LRP, (**IV**) C5_C1_LRP, (**V**) C4_C1_LRP, and (**VI**) C7_C3_LRP septanosyl Ferrier cations. Hydrogen atoms are omitted for clarity.

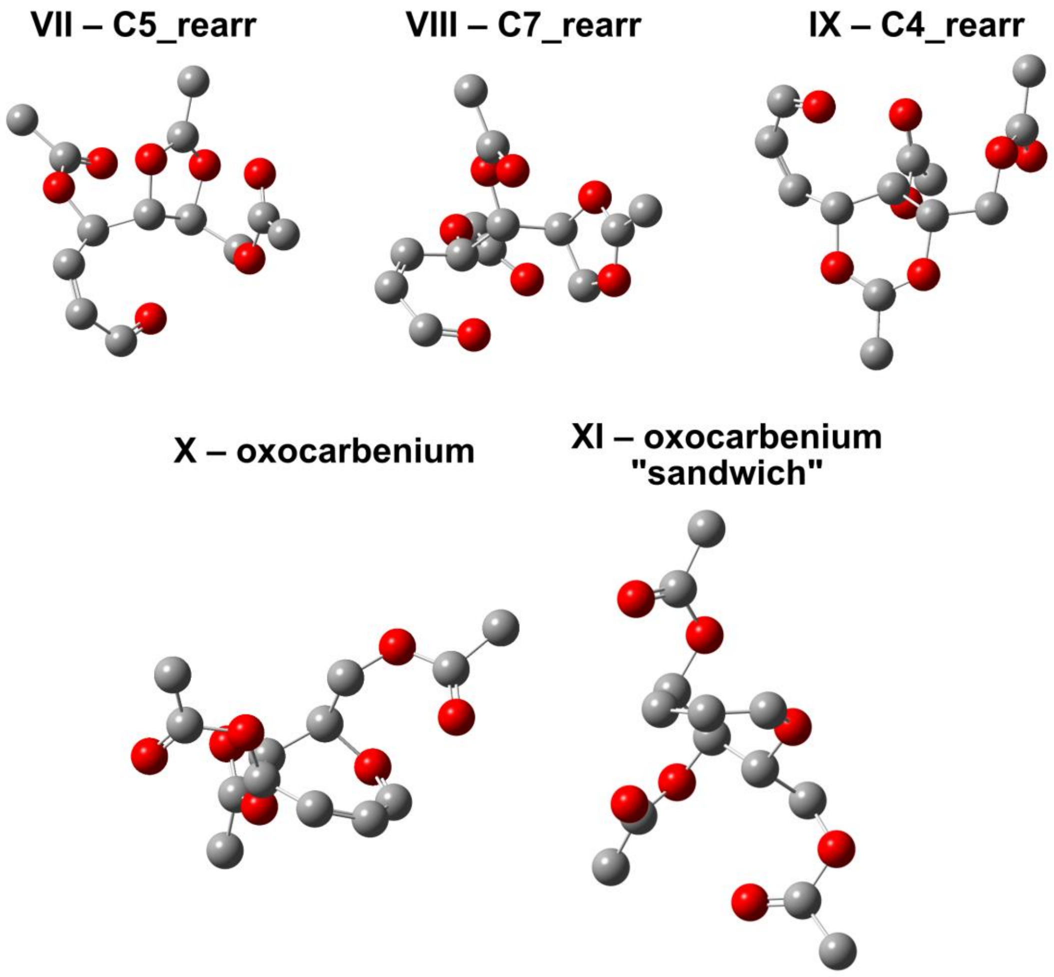


**Figure S5.** Selected reoptimized 3D-geometries of low-energy structures of (**VII**) C5_rearranged, (**VIII**) C7_rearranged, (**IX**) C4_rearranged, (**X**) oxocarbenium, and (**XI**) oxocarbenium “sandwich” septanosyl Ferrier cations. All rearranged structures are shown as R/S/S (C4/C5/C6) diastereomers. Hydrogen atoms are omitted for clarity.

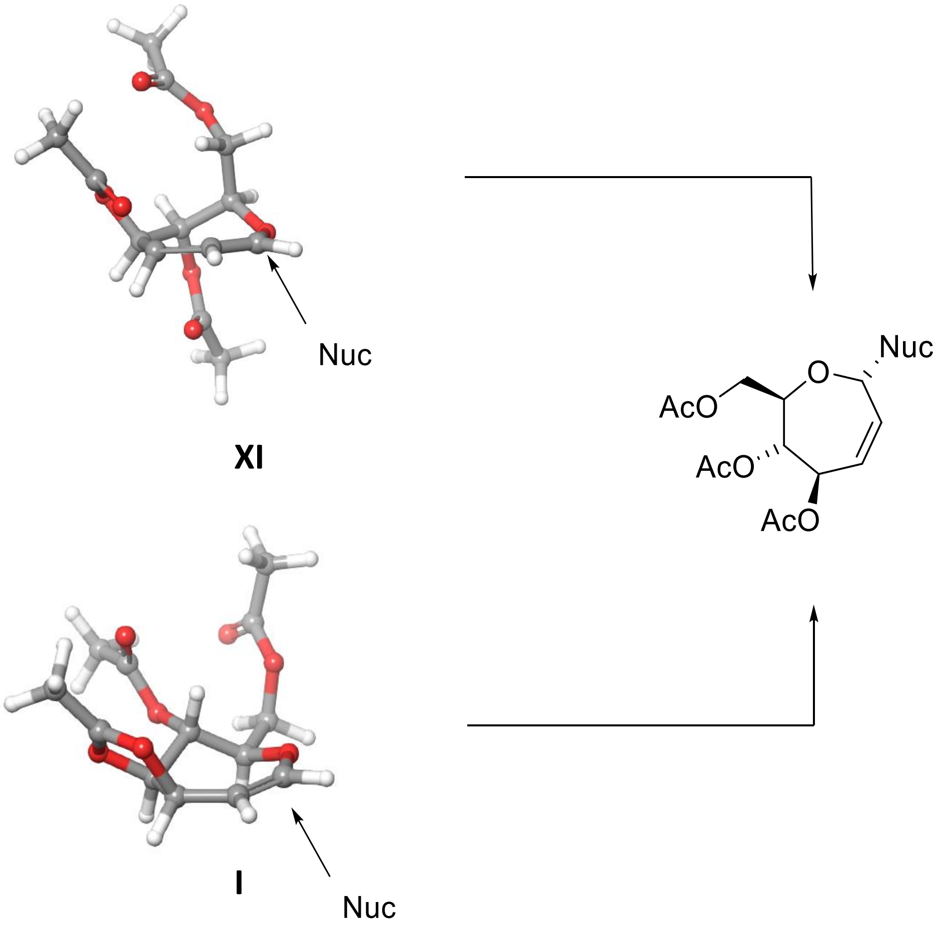


**Figure S6.** 3D structures of C4_C3_NGP (**I**) and the oxocarbenium "sandwich" (**XI**) with nucleophilic path of attack.

**Table S3.** Tabulated NMR data for compounds 15, 16, and 18

| Compound | Key chemical shifts (δ ppm) | | | | Key Observations |
|---|---|---|---|---|---|
| | $^{1}$H | | $^{13}$C{$^{1}$H} | | |
| 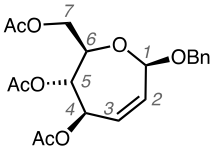<br>**15**<br>$CDCl_3$ | H1 5.40<br>H2 5.76<br>H3 5.91<br>H4 5.27<br>H5 5.20<br>H6 4.37<br>H7/7’ 4.14-4.26 | | C1 98.3<br>C2 132.3<br>C3 126.6<br>C4 70.8<br>C5 73.1<br>C6 68.9<br>C7 63.7 | | H1-H6 NOE |
| | **major** | **minor** | **major** | **minor** | |
| 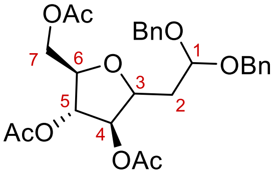<br>**16**<br>$CDCl_3$ | H1 4.93<br>H2 2.04<br>H3 4.17<br>H4 5.15<br>H5 4.91<br>H6 3.92<br>H7/7’<br>4.33/4.16 | H1 4.97<br>H2 2.11<br>H3 4.24<br>H4 5.10<br>H5 5.08<br>H6 4.16<br>H7/7’ 4.24 | C1 99.92<br>C2 32.8<br>C3 77.14<br>C4 77.41<br>C5 78.97<br>C6 81.23<br>C7 63.84 | C1 99.74<br>C2 36.70<br>C3 80.00<br>C4 80.53<br>C5 78.76<br>C6 80.61<br>C7 63.52 | H3-C6 HMBC<br>Major: H3-H6 NOE |
| 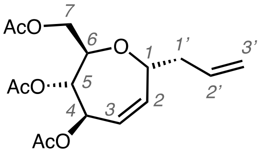<br>**18**<br>acetone- $d_6$ | H1 4.51<br>H2 5.74<br>H3 5.53<br>H4 5.79<br>H4 5.79<br>H5 5.02<br>H6 4.09<br>H7 4.08<br>H7 4.42<br>H1’ 2.33<br>H2’ 5.9<br>H3’ 5.04-5.17 | | | | H1-H5 NOE |

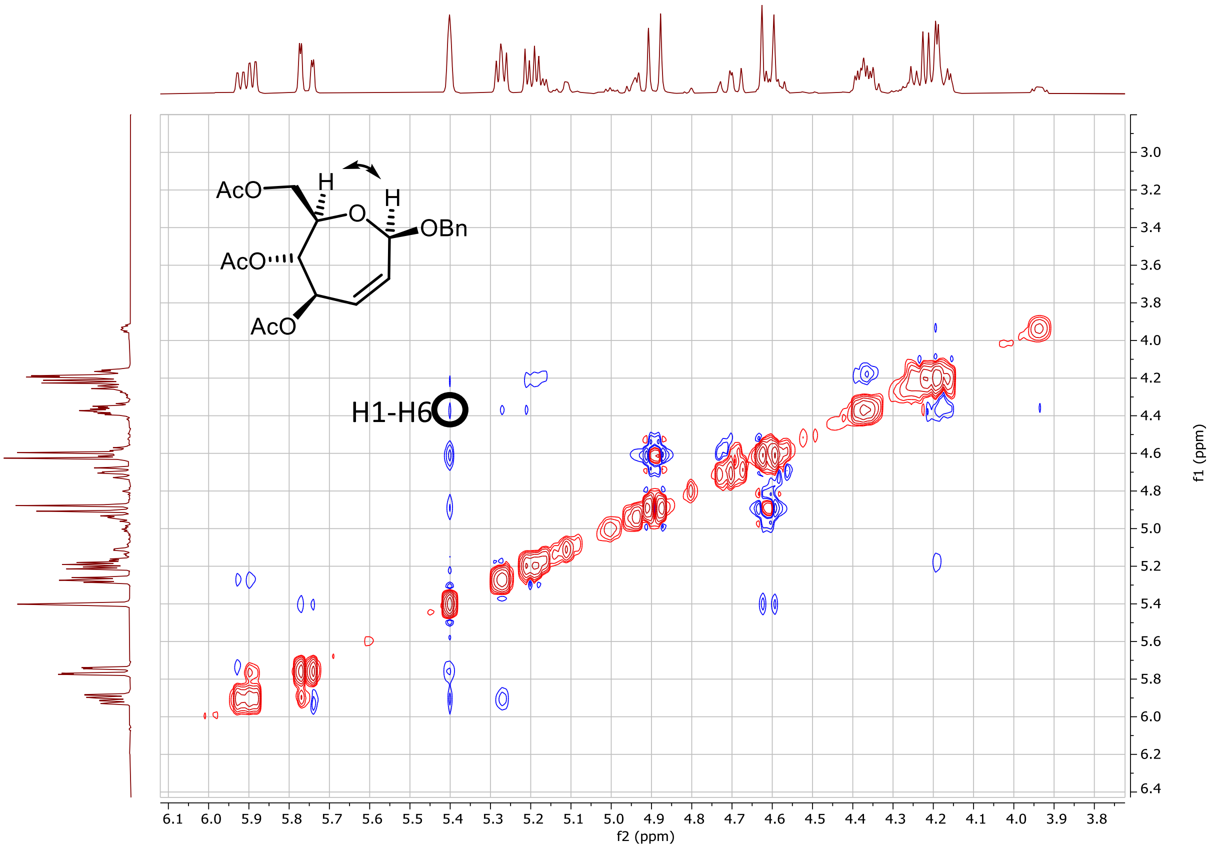


**Figure S7.** NOESY NMR of the 15/16 mixture showing H1-H6 NOE interaction.

**Analysis of NMR spectra to support structure of *C*-methylene-acetal arabinofuranoside 16**

<u>HSQC data</u>

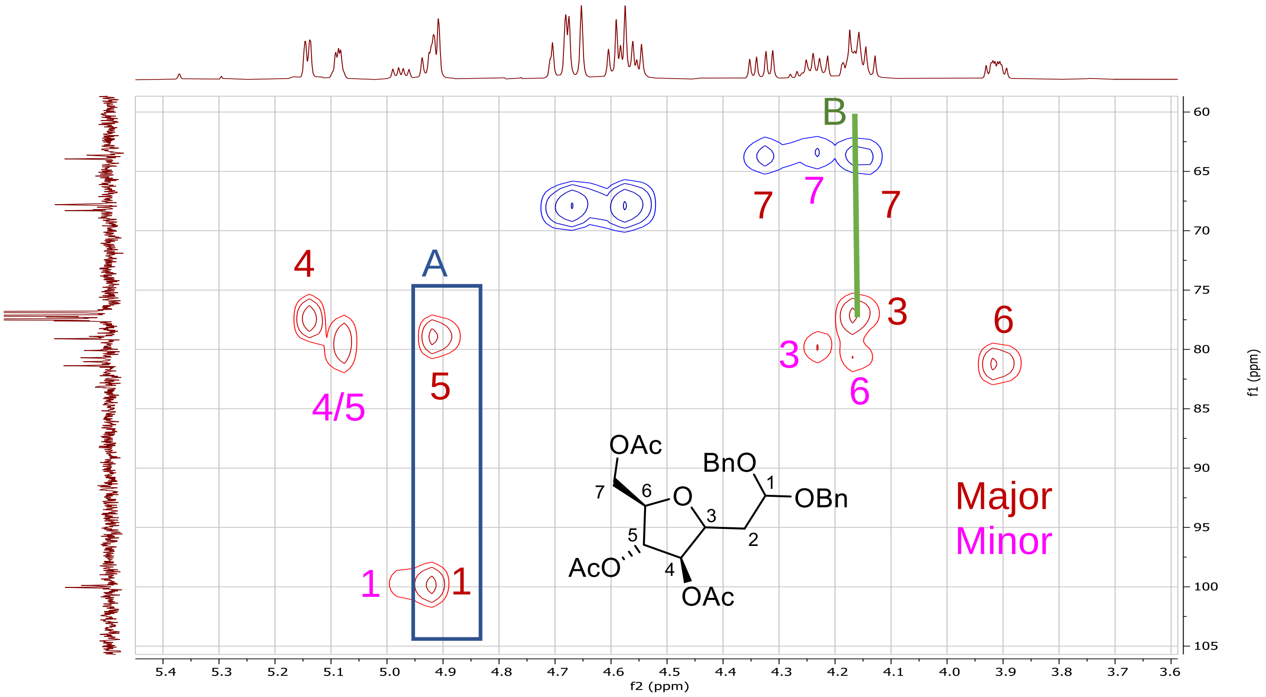


**Figure S8.** Zoomed version of the HSQC with assignments.

A) Acetal proton (H1; 4.9 ppm) overlaps with another CH group, but it is slightly more downfield. This multiplet integrated to 2H suggesting it is from the same molecule.

B) H3 of the major product is slightly more downfield than one of the H7 diastereotopic protons.

COSY data

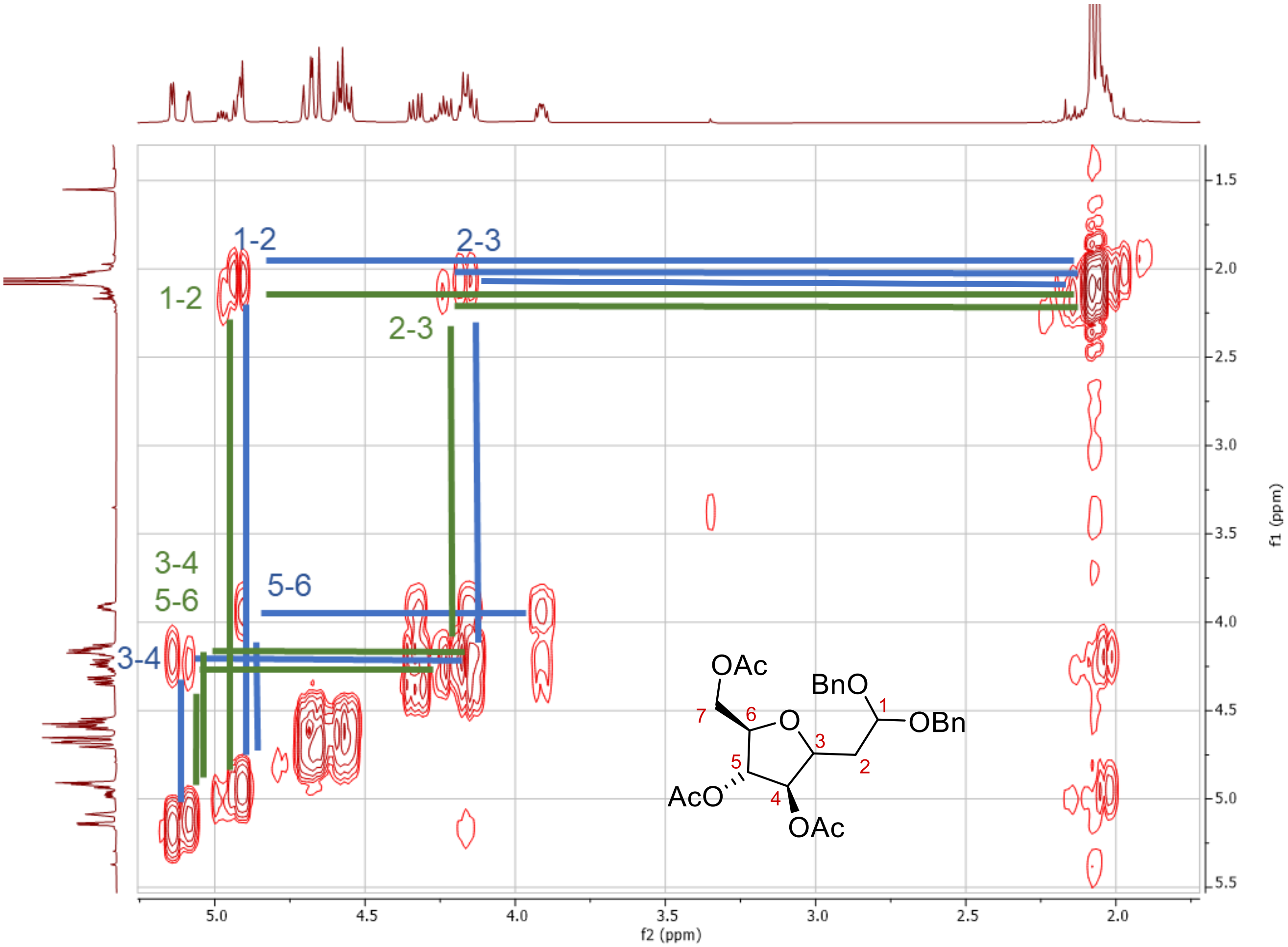


**Figure S9.** COSY view of nonaromatic protons with correlations of the major isomer.

Blue line: major isomer correlations

Starting at the major acetal proton (H1) at 4.95, there is a correlation to the $CH_2$ (H2) hidden under the acetate methyl groups. The hidden methylene also correlates to a CH group hidden in a multiplet at 4.1 ppm (H3). This, in turn correlates to another CH (H4). COSY correlation ends here after 4 groups. However, H4 and H5 are shown to correlate in TOCSY experiments (Figure S6). Based on that experiment, H5 is the proton that overlaps with the acetal proton and correlates to H6. H6 is connected to the acetoxymethyl rotamer since it correlates to H7 and H7'.

Green line: minor isomer correlations

In the minor product, the acetal proton is more downfield than the major. The dd at ~5 ppm is H1. This correlates to the hidden $CH_2$ group (H2). H2 correlates to the multiplet at 4.26 ppm (H3) which, in turn, correlates to the H4 at 5.1 ppm. Again, H4 and H5 do not correlate by COSY. In this case H4 and H5 overlap at 5.1 ppm. This peak correlates to the multiplet at 4.17 ppm (H6). In this case, the H6 proton is overlapped by the major H7 and H3 protons but can be observed to be unique in the HSQC.

TOCSY data

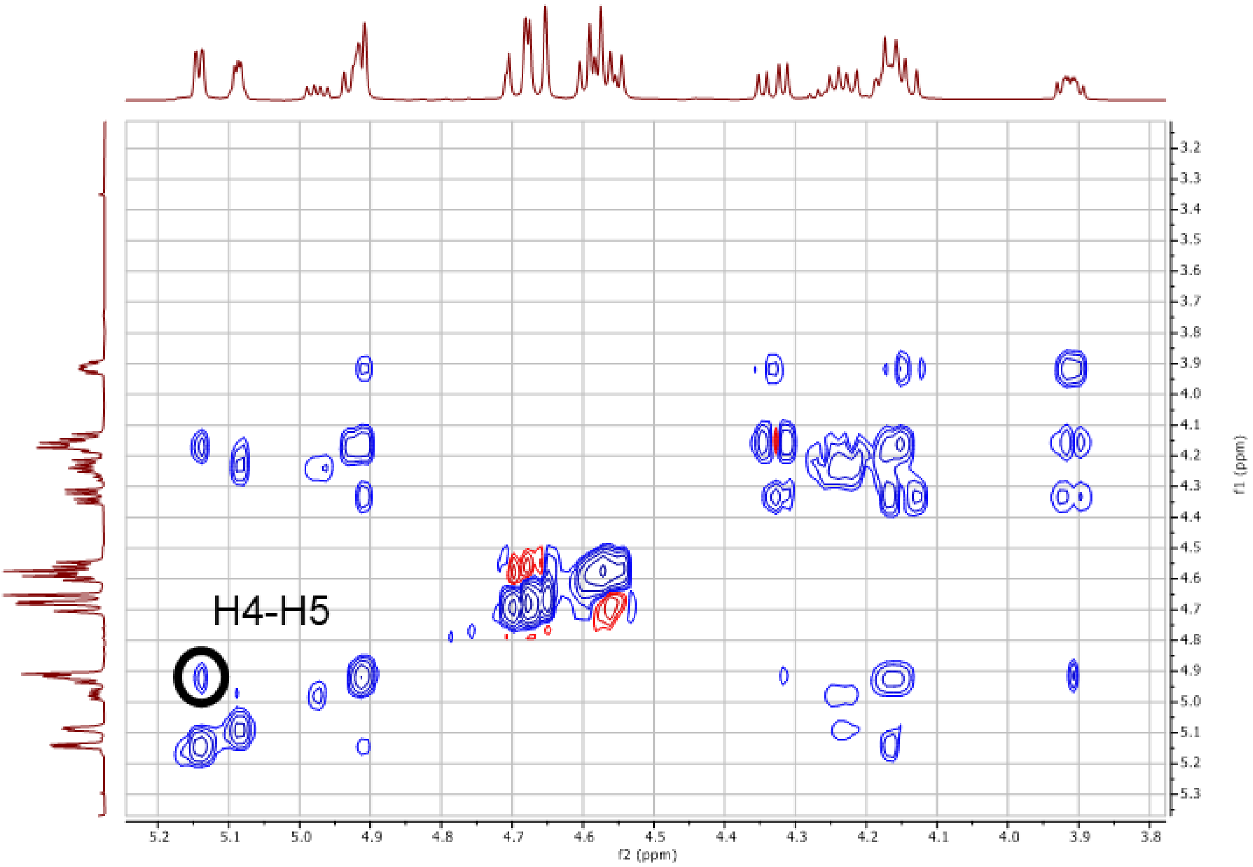


**Figure S10.** TOCSY experiment showing H4 and H5 cross peak.

**Table S4.** Tabulated chemical shifts of the 16.

| Position | Degree of substitution | Major | | Minor | |
|---|---|---|---|---|---|
| | | $^{1}$H | $^{13}$C{$^{1}$H} | $^{1}$H | $^{13}$C{$^{1}$H} |
| 1 | CH (acetal) | 4.93 | 99.92 | 4.97 | 99.74 |
| 2 | $CH_2$ (diastereotopic) | 2.04 | 32.8 | 2.11 | 36.70 |
| 3 | CH | 4.17 | 77.14 | 4.24 | 80.00 |
| 4 | CH | 5.15 | 77.41 | 5.10 | 80.53 |
| 5 | CH | 4.91 | 78.97 | 5.08 | 78.76 |
| 6 | CH | 3.92 | 81.23 | 4.16 | 80.61 |
| 7 | $CH_2$ (diastereotopic) | 4.33 | 63.84 | 4.24 | 63.52 |
| 7’ | $CH_2$ (diastereotopic) | 4.16 | 63.84 | 4.24 | 63.52 |
| Benzyl | $CH_2$ (diastereotopic) | 4.54-4.72 | 67.82 | 4.54-4.72 | 67.82 |

<u>HMBC data</u>

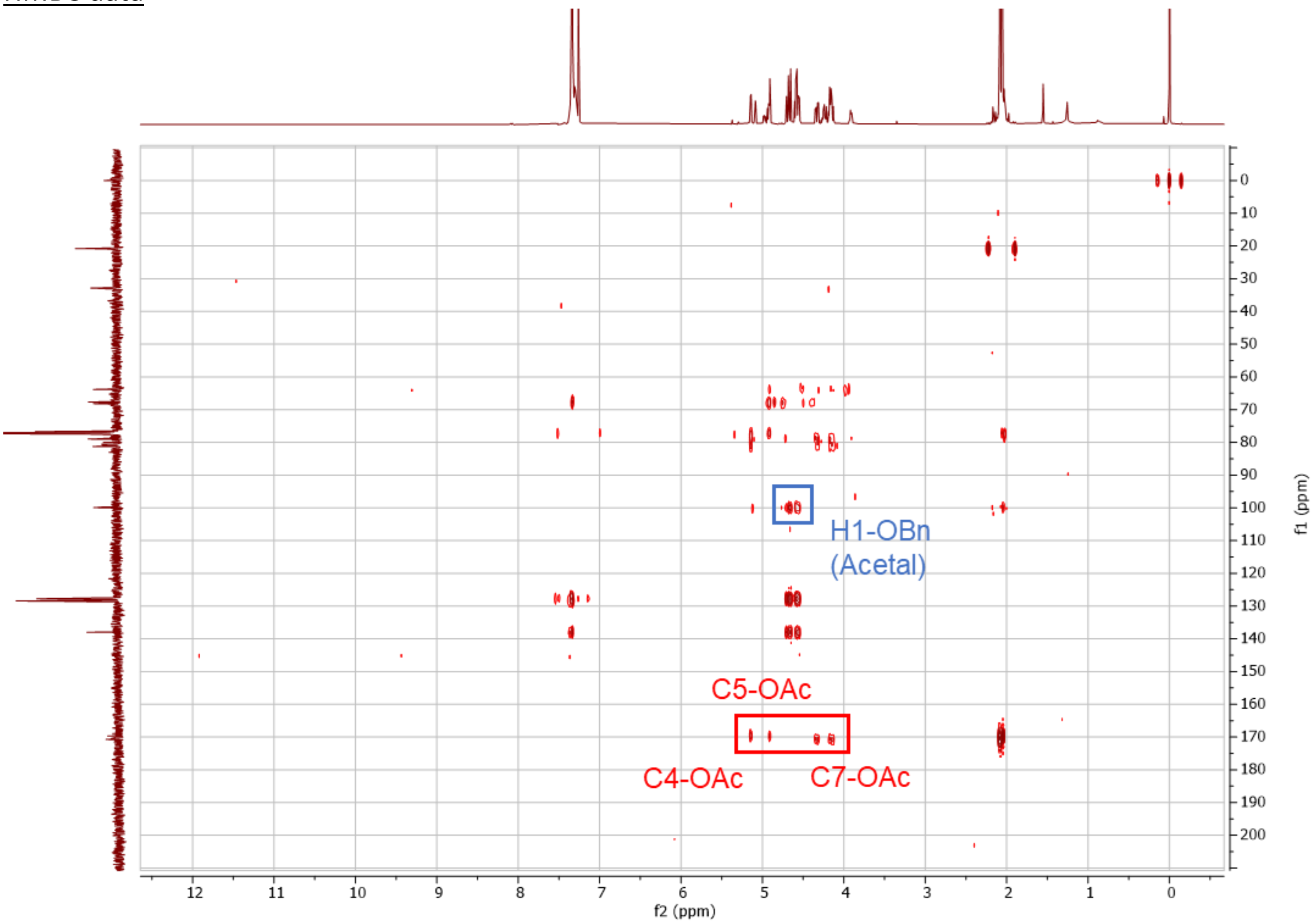


**Figure S11.** HMBC spectrum showing acetate cross peaks (red circles), benzyl-acetal cross peak (green circle), and C6-H3 cross peak.

Carbonyls (acetates) correlate with the H4, H5, and H7 (red square). Also, the HMBC shows that the acetal is a dibenzyl acetal (blue square).

Using the NMR information, the molecule is constructed as below. C6 and C3 still have one site to fill.

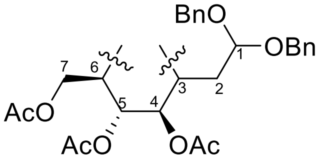

Its molecular weight is 484.54 and m/z 523.1927 [M+Na] was found. A mass of 16.00 is missing. Linking the C6 and C3 with an oxygen will account for the mass discrepancy to create the below molecule.

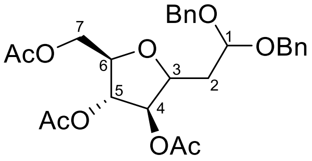

Stereochemistry at C3

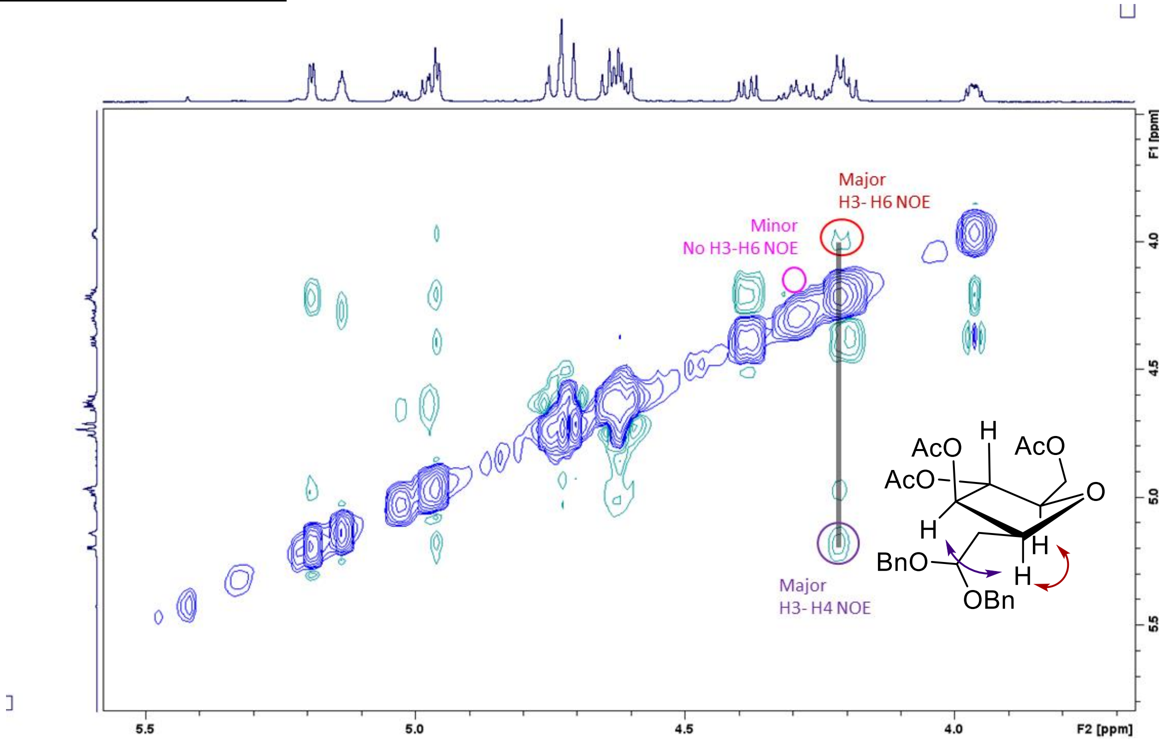


**Figure S12.** Zoomed version of the NOESY spectrum.

In the NOESY above, two different cross peaks suggest the major product is the *S* stereoisomer. The gray line corresponds to the major H3 chemical shift which is demonstrated in Figure S7. Along it there is a H3-H4 NOE (purple), a H3-H6 NOE (red). Together, this suggests that the H3 is on the same face as H4 and H6.

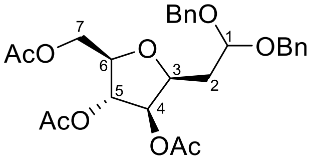

For the minor product, there is no NOE cross peak between H3 and H6 (pink circle), suggesting that they are on different faces of the molecule.

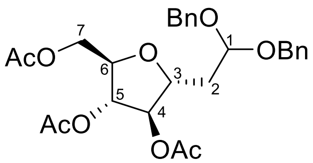


There is a 2:1 ratio between isomers. This means that 15 benzyl protons and 16.5 acetate/$CH_2$ protons are expected. Which matches the $^{1}$H NMR.

**Table S5.** Common abbreviations used in the main text.

| Abbreviations | Definitions |
|---|---|
| DMDO | Dimethyldioxirane |
| HFIP | Hexafluoroisopropanol |
| NGP | Neighboring-group participation |
| LRP | Long-range participation |
| TOCSY | Total correlation spectroscopy |
| NOE | Nuclear Overhauser effect |
| NOESY | Nuclear Overhauser effect spectroscopy |
| HSQC | Heteronuclear single quantum coherence |
| HMBC | Heteronuclear multiple bond correlation |
| BRSM | Based on recovered starting material |

**$^{1}$H NMR of compound 8**

$^{1}$H NMR (500 MHz, d1-chloroform)

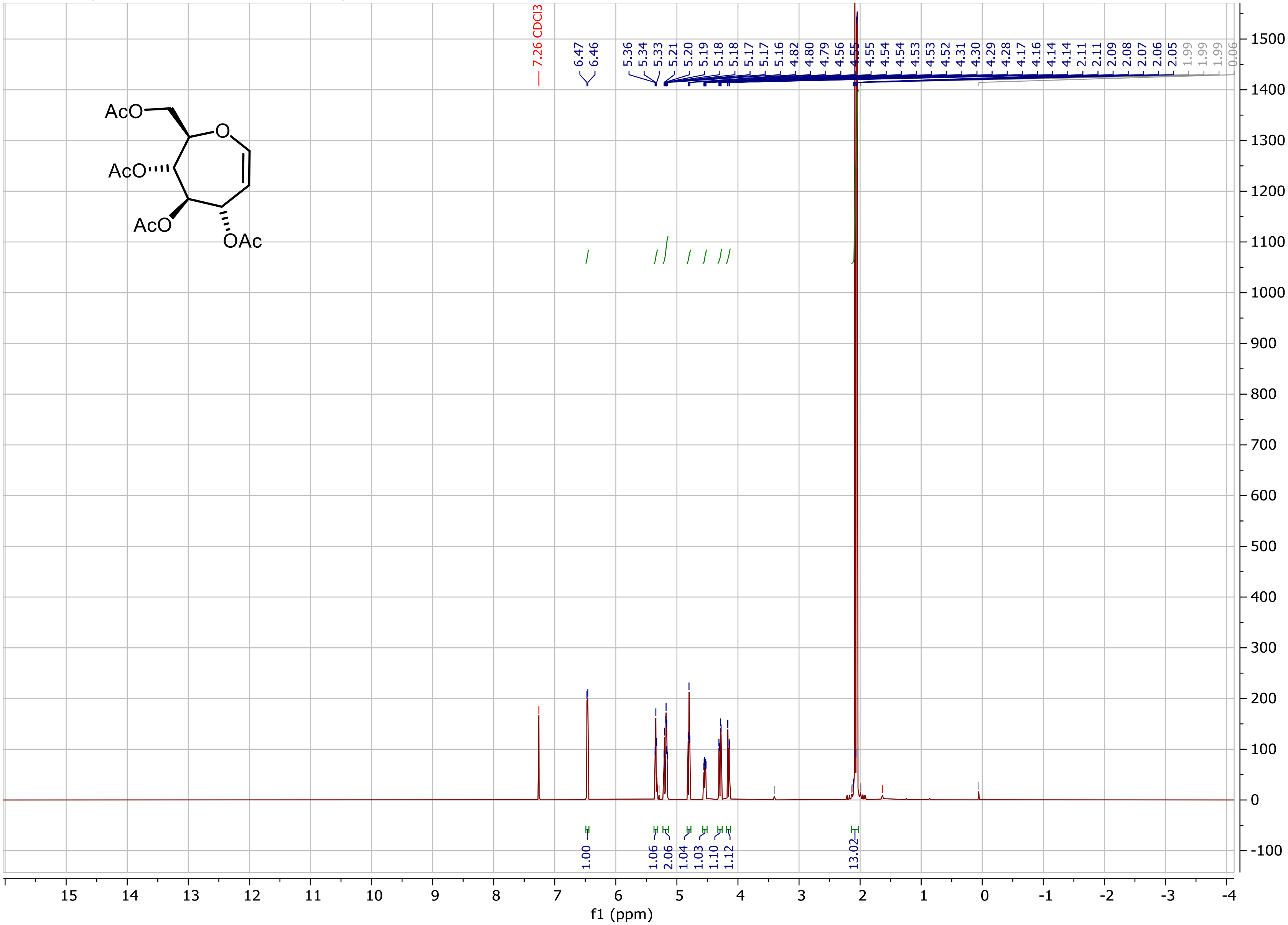

**$^1$H NMR of compound 10**

$^1$H NMR (500 MHz, d1-chloroform)

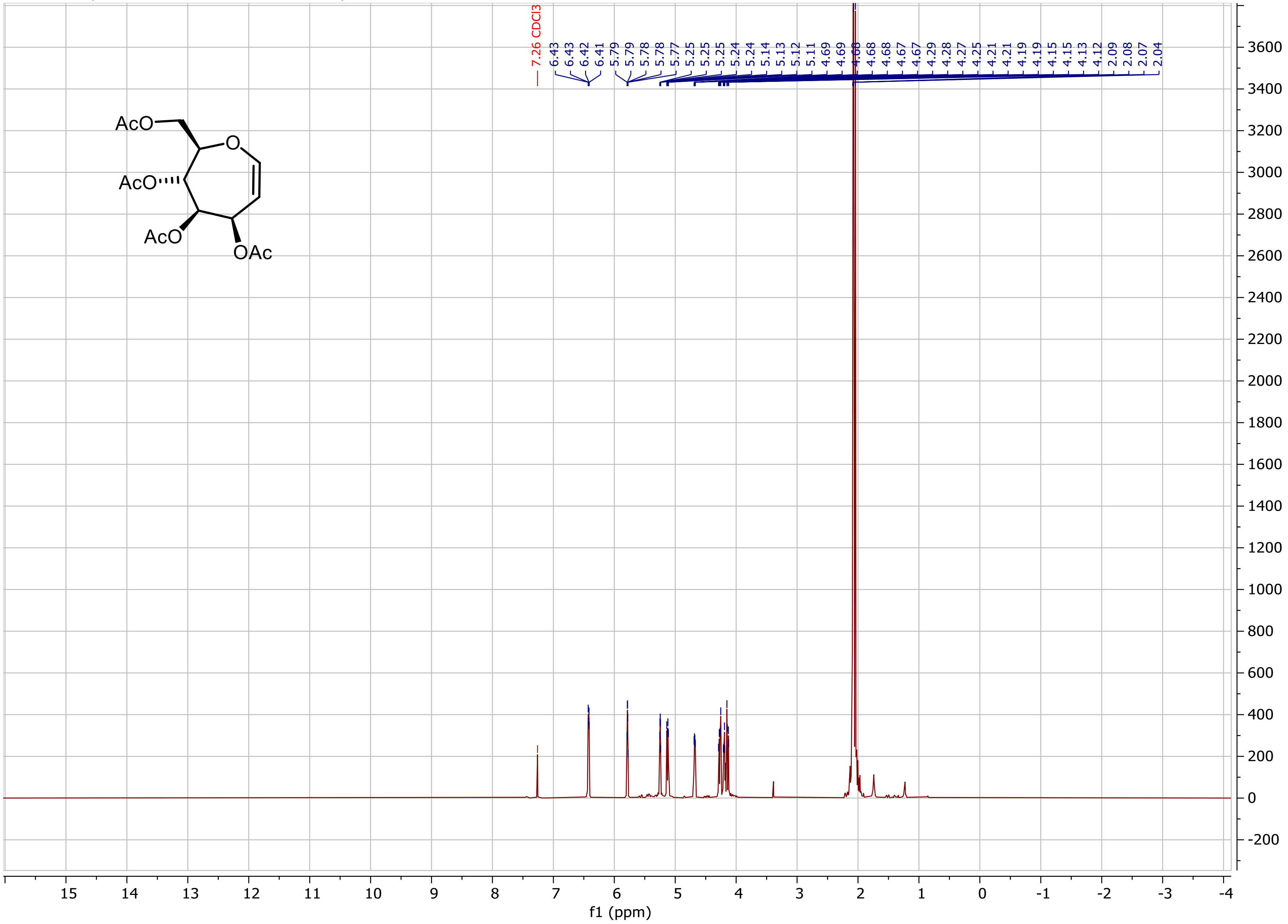

**NMR spectra used to characterize compound 15**

$^1$H NMR (400 MHz, d1-chloroform)

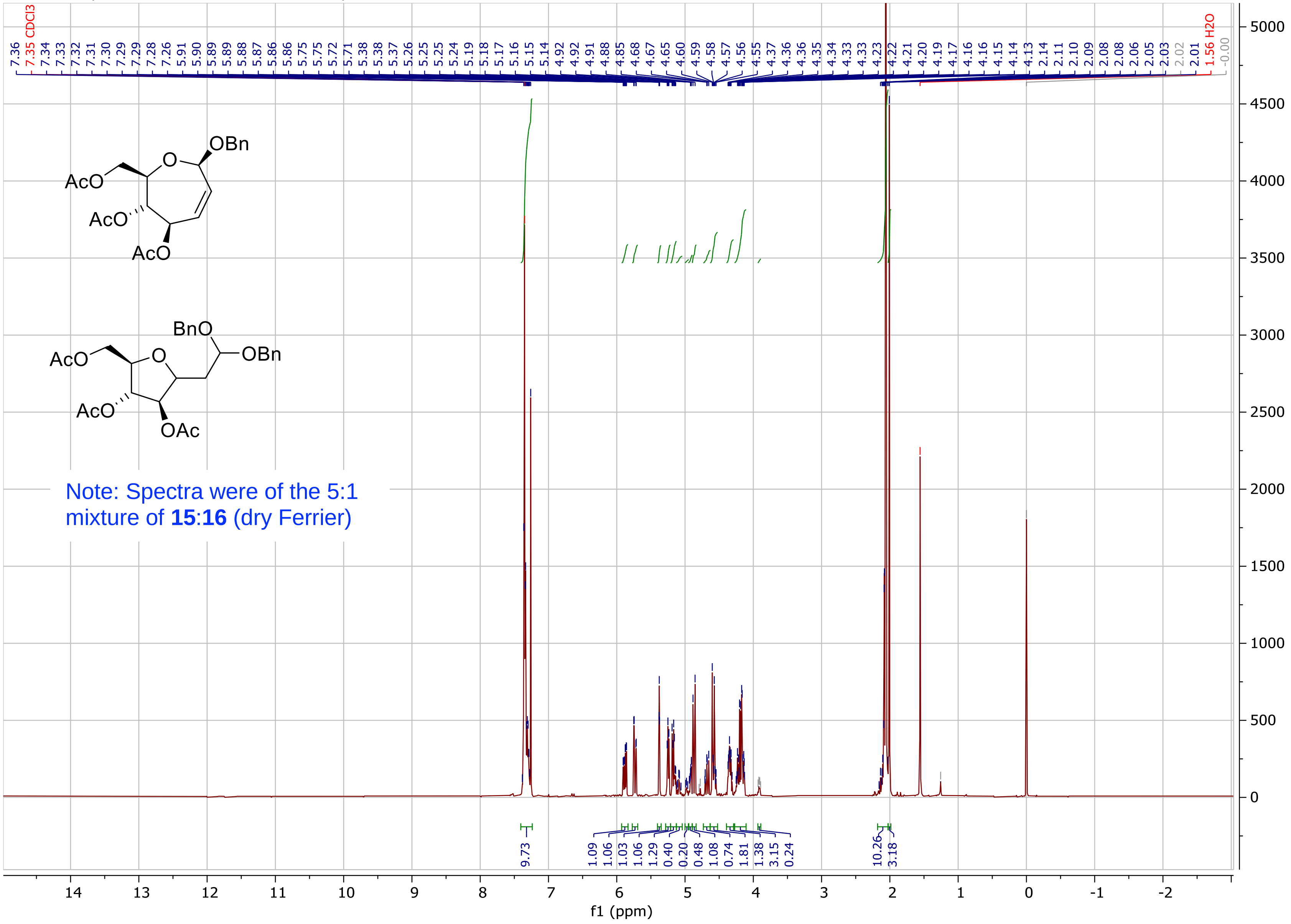

$^{13}C\{^1H\}$ NMR (100 MHz, $CDCl_3$)

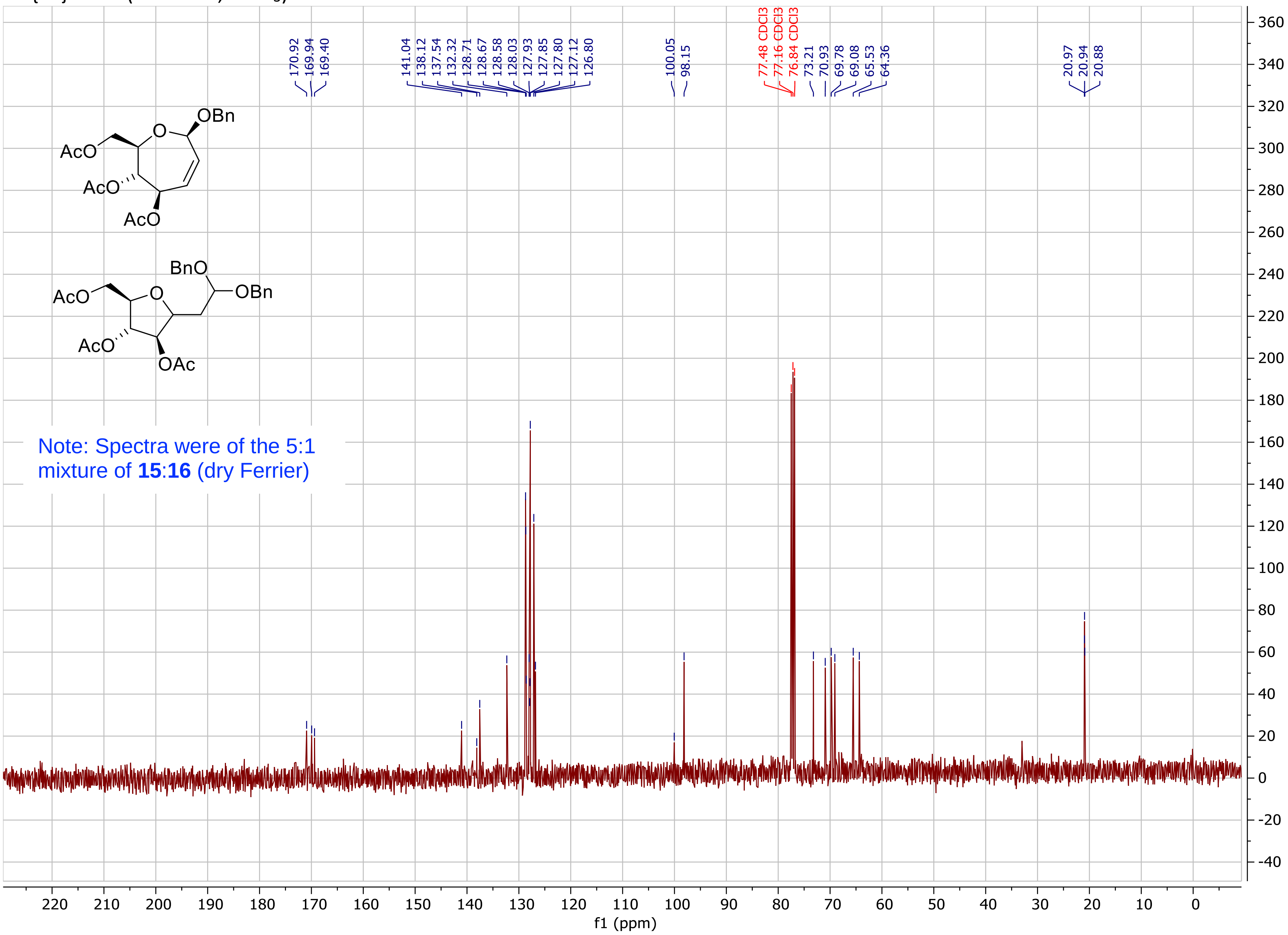

COSY NMR (400 MHz, $CDCl_3$)

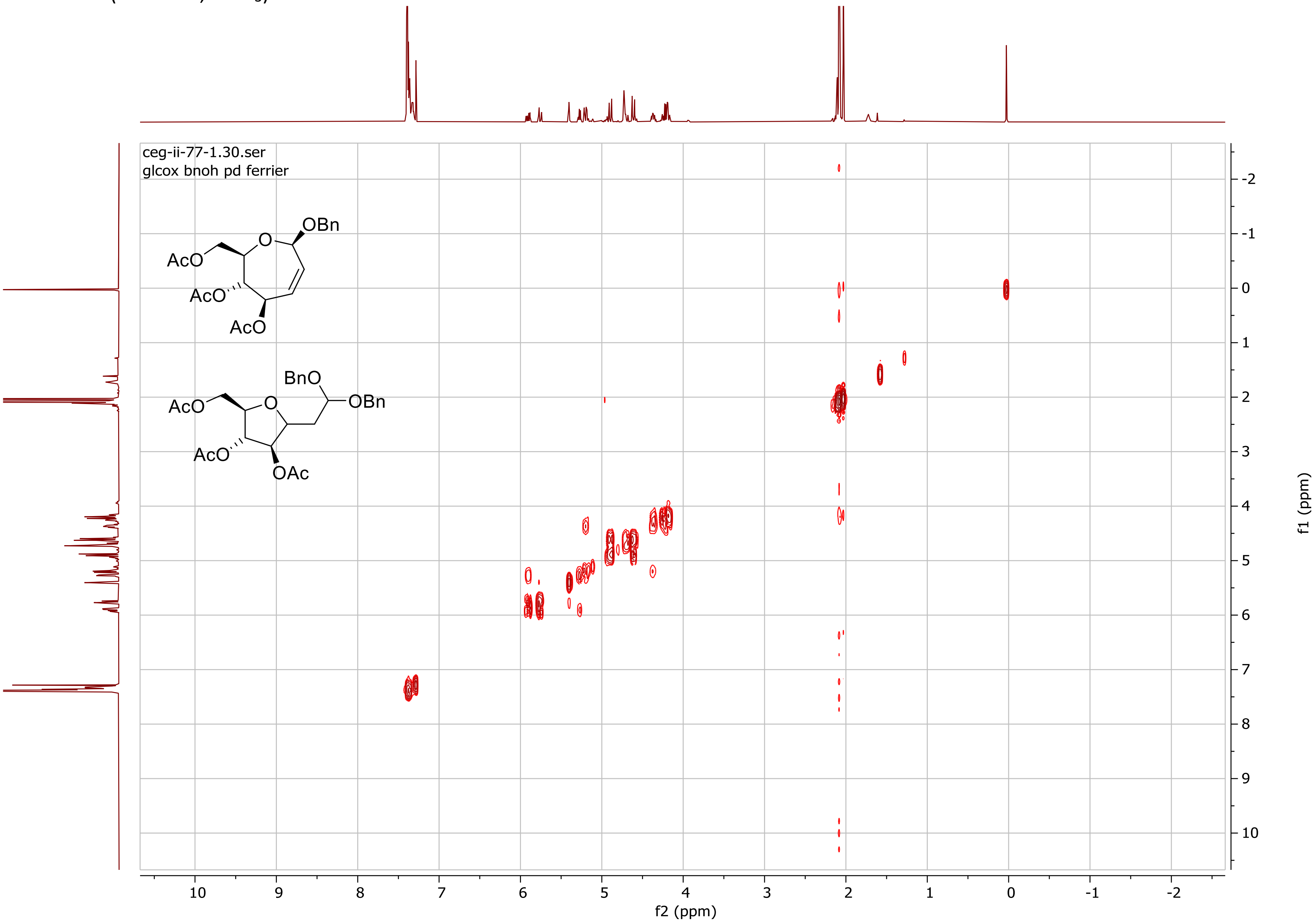

HSQC NMR (400 MHz, d1-chloroform)

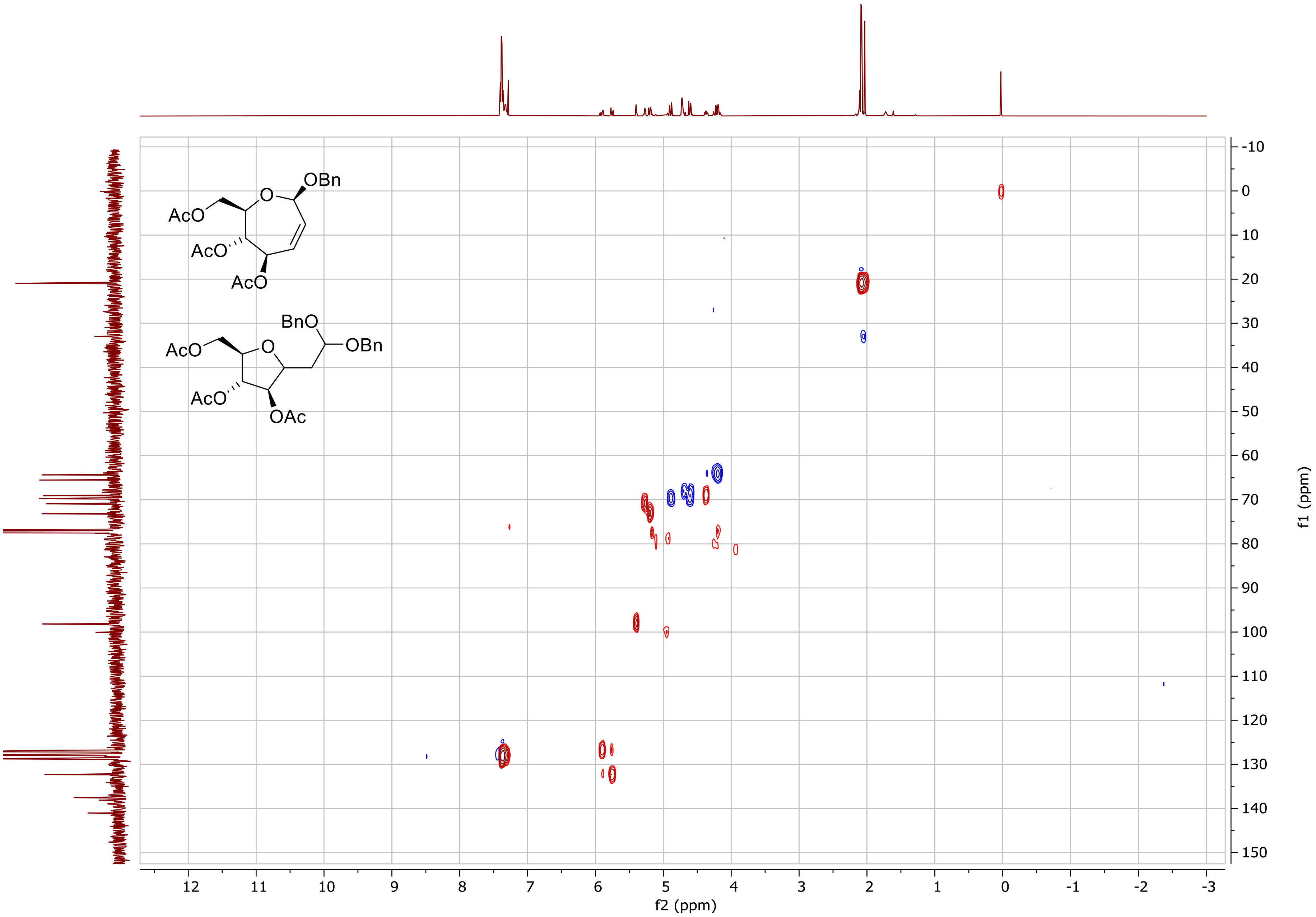

HMBC NMR (400 MHz, d1-chloroform)

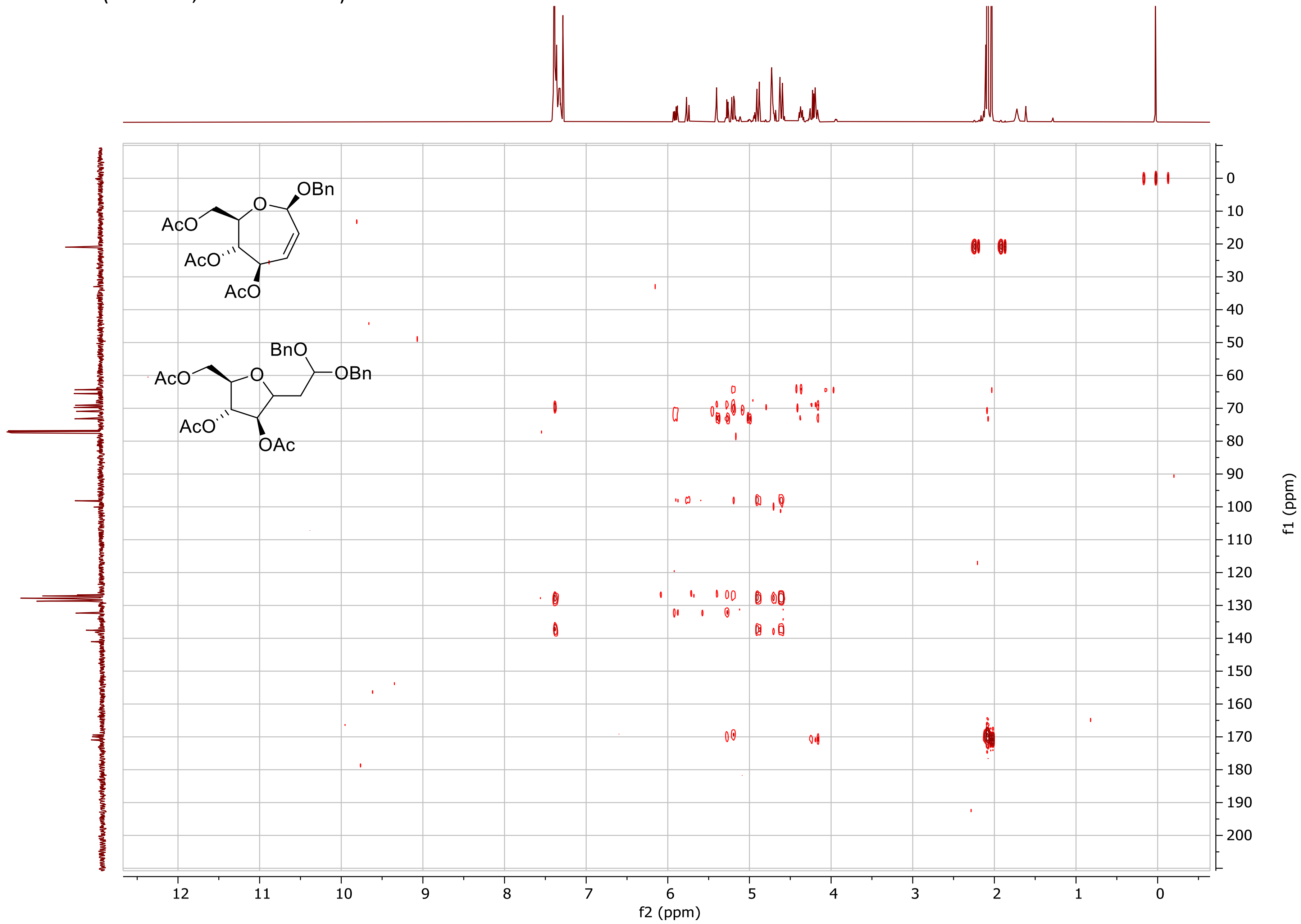

NOESY NMR (400 MHz, d1-chloroform)

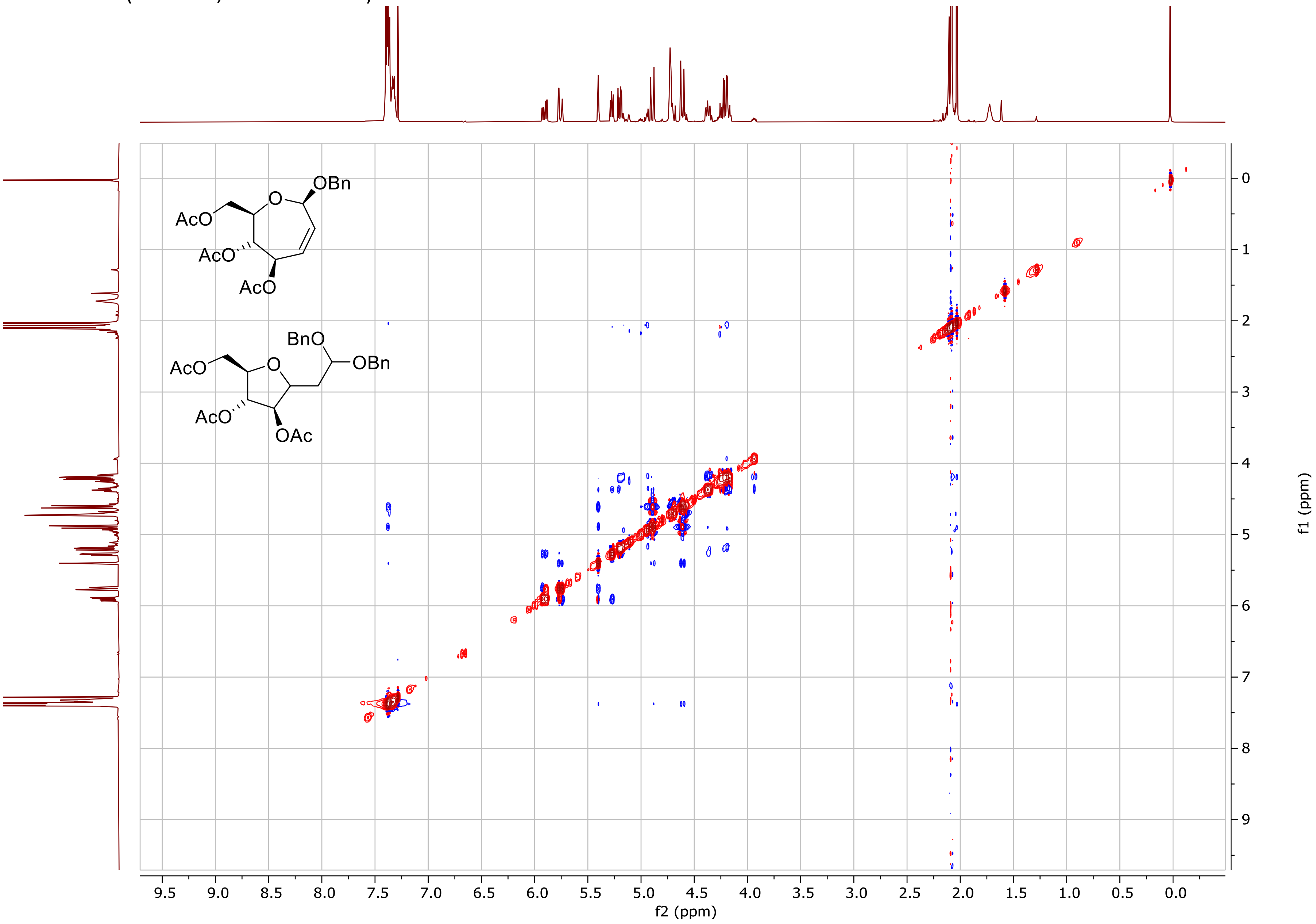

**NMR characterization data of 16 – wet Ferrier product**

$^{1}$H NMR (400 MHz, d1-chloroform)

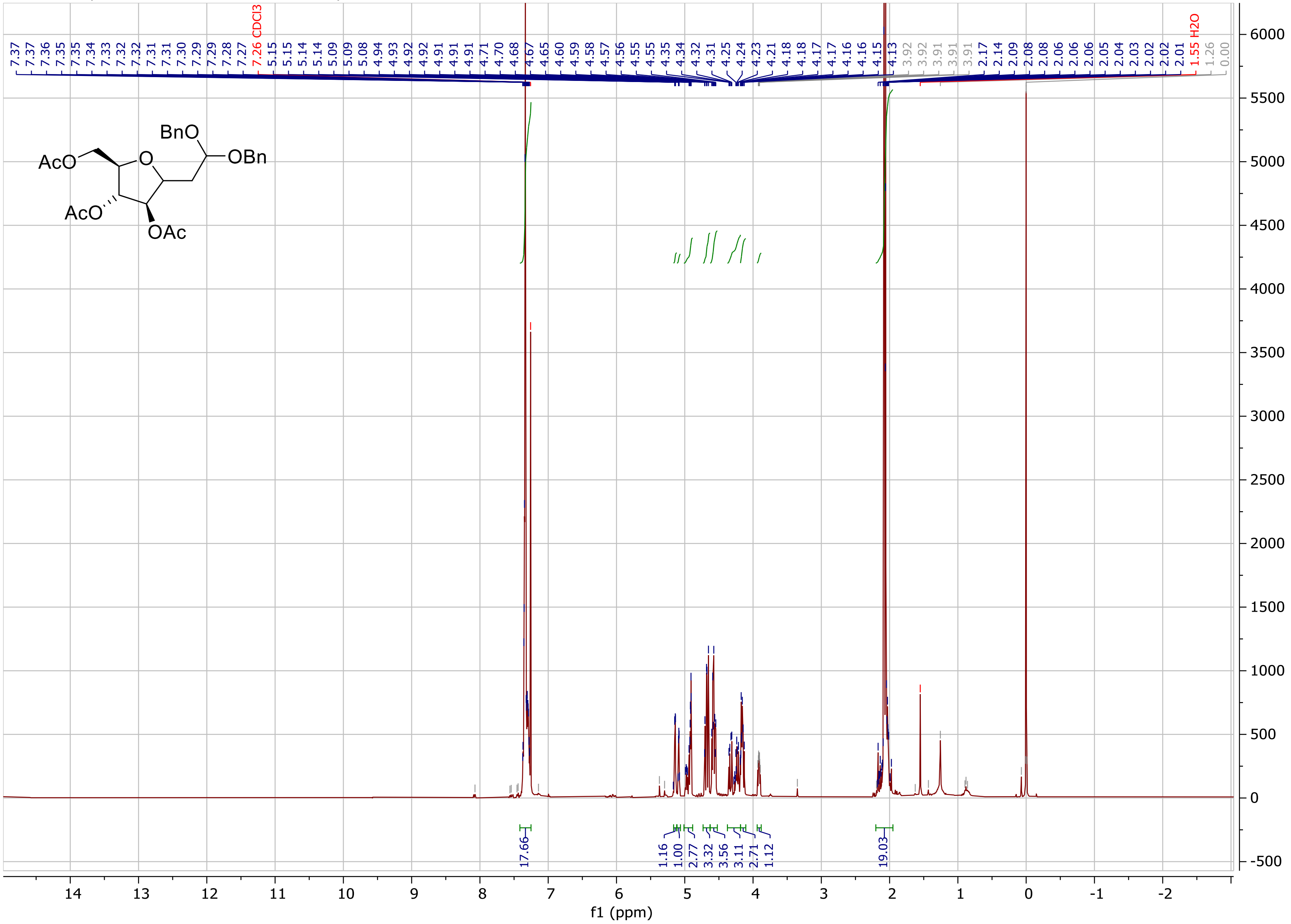

$^{13}C\{^1H\}$ NMR (100 MHz, d1-chloroform)

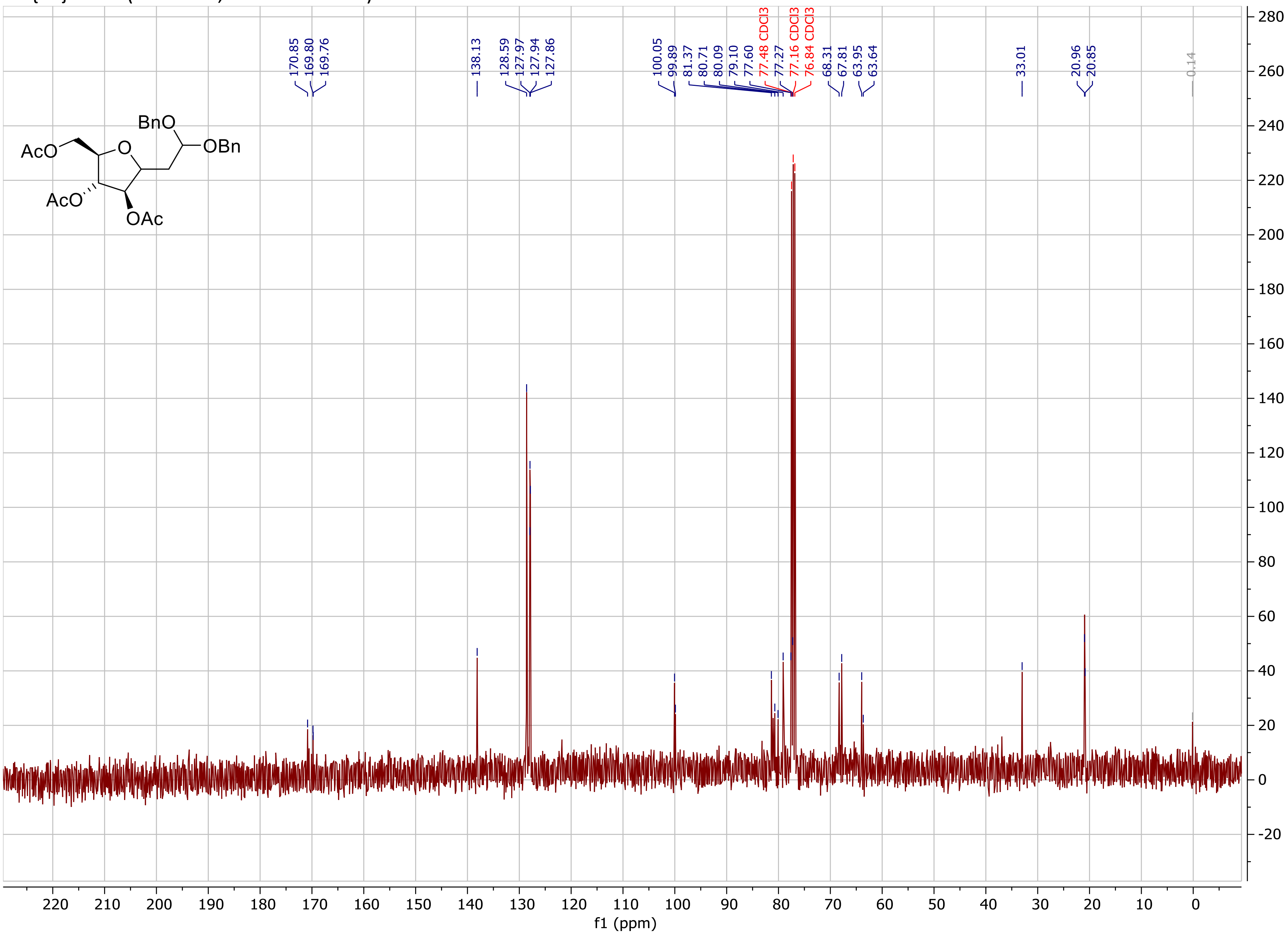

COSY NMR (400 MHz, d1-chloroform)

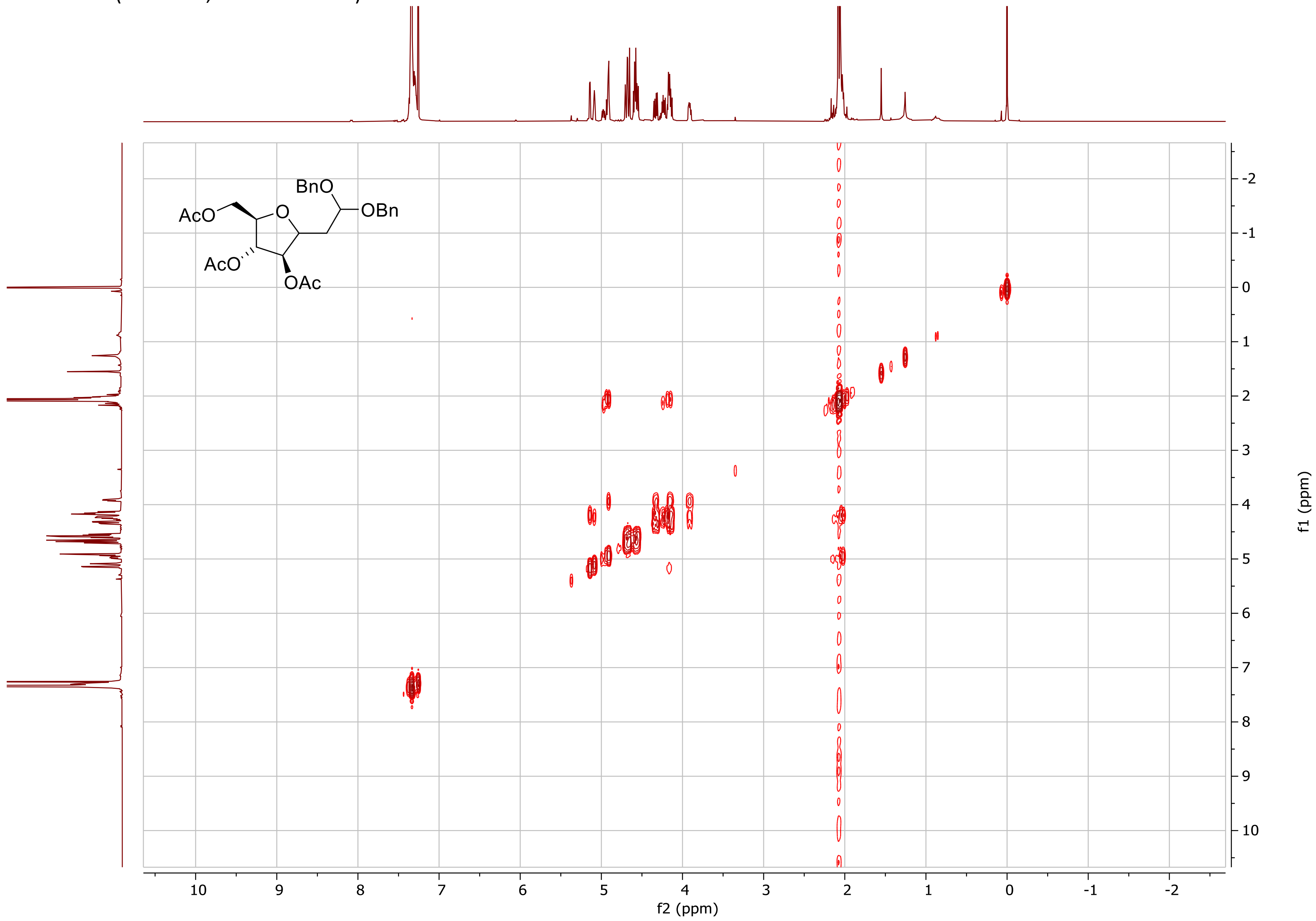

HSQC NMR (400 MHz, d1-chloroform)

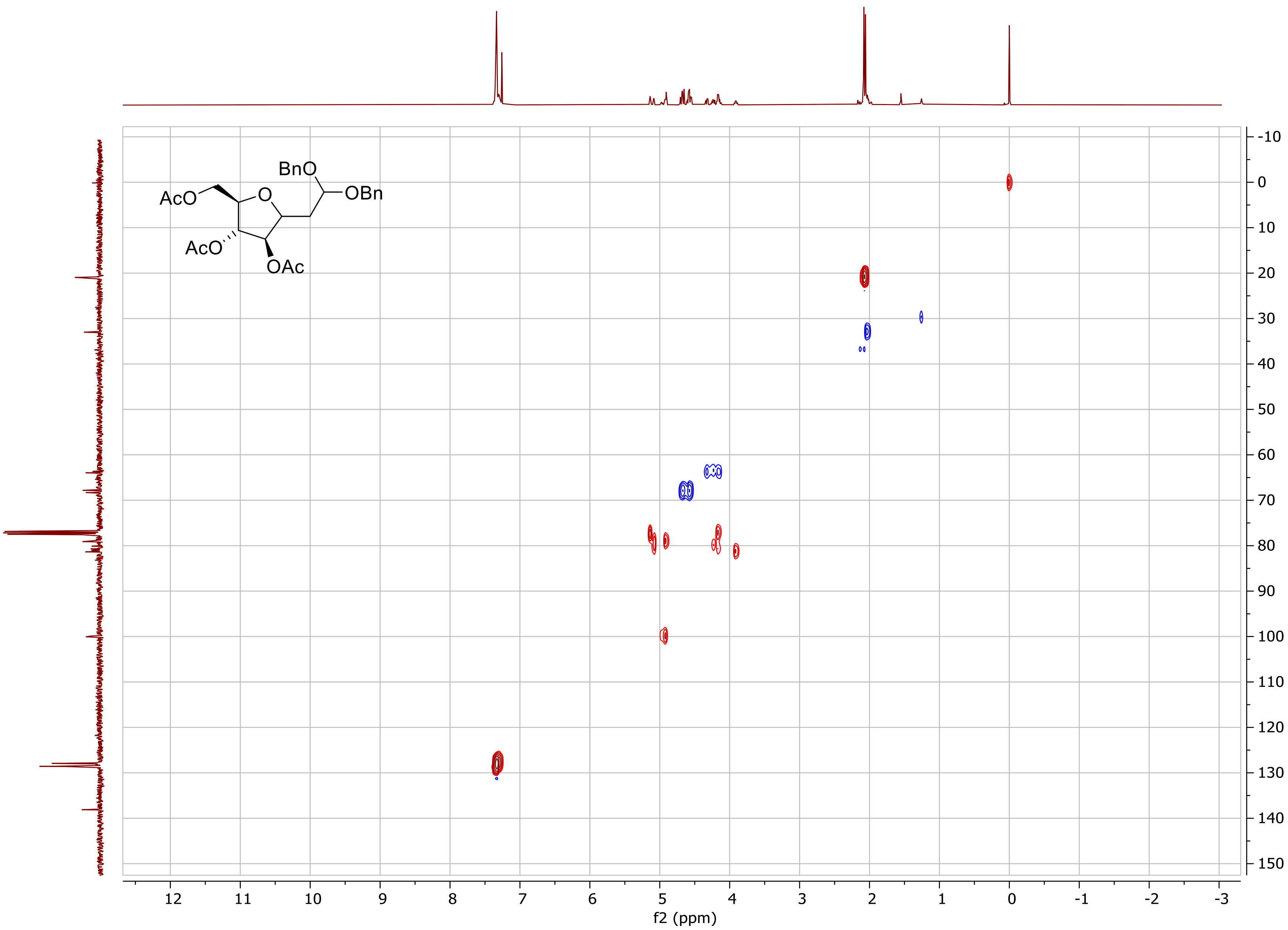

HMBC NMR (400 MHz, d1-chloroform)

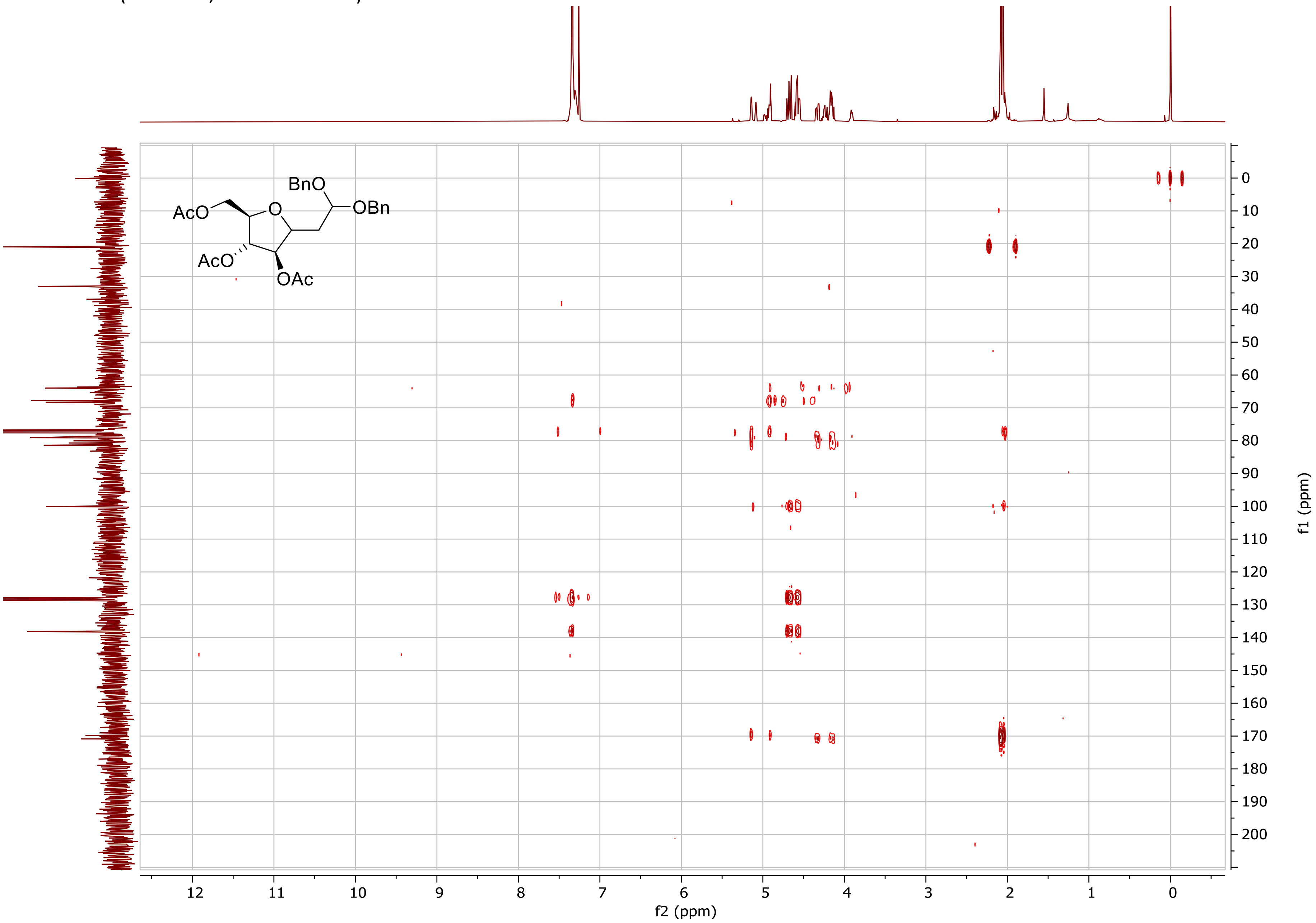

NOESY NMR (400 MHz, d1-chloroform)

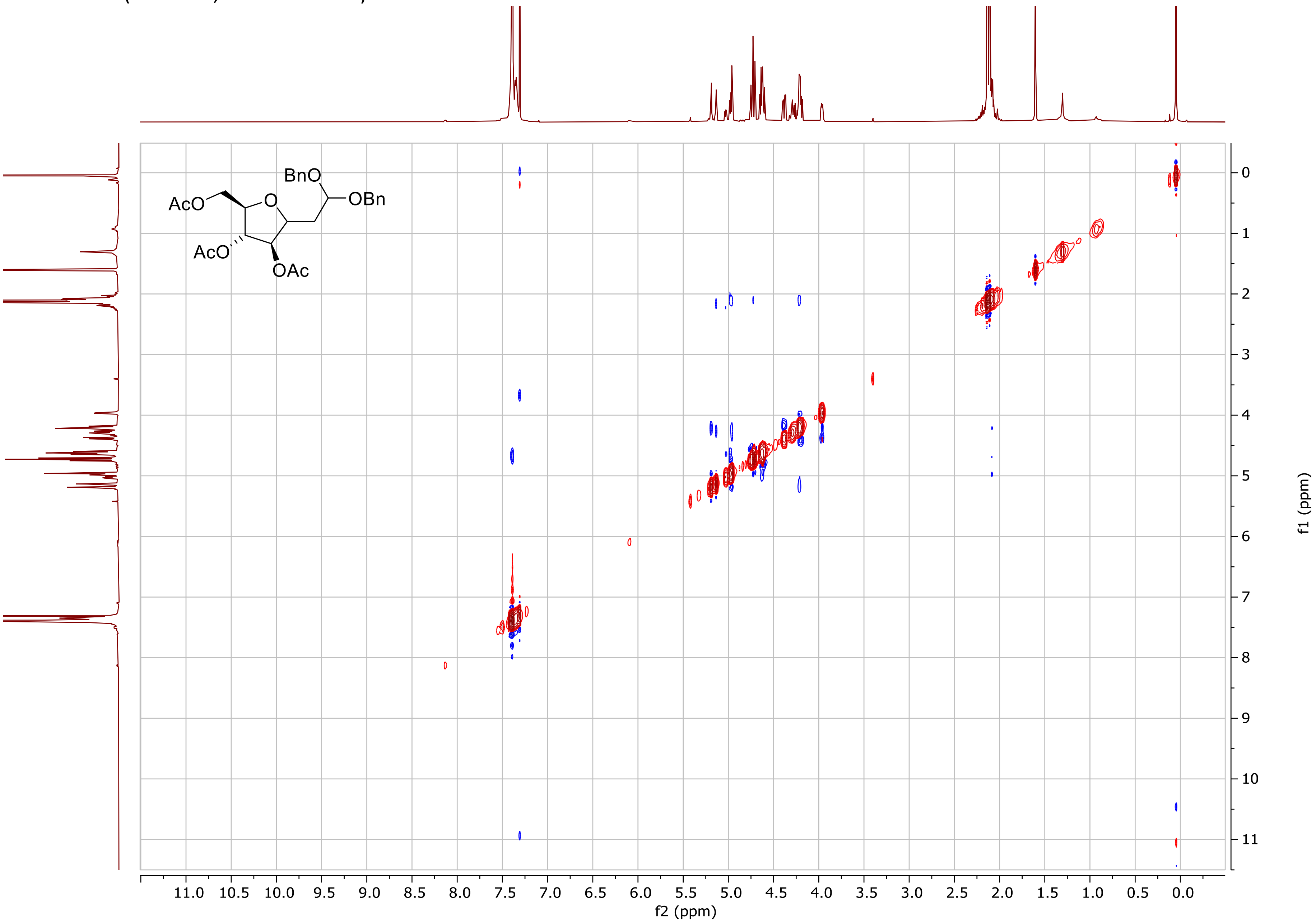

**NMR characterization data of 17**

$^{1}$H NMR (400 MHz, d1-chloroform)

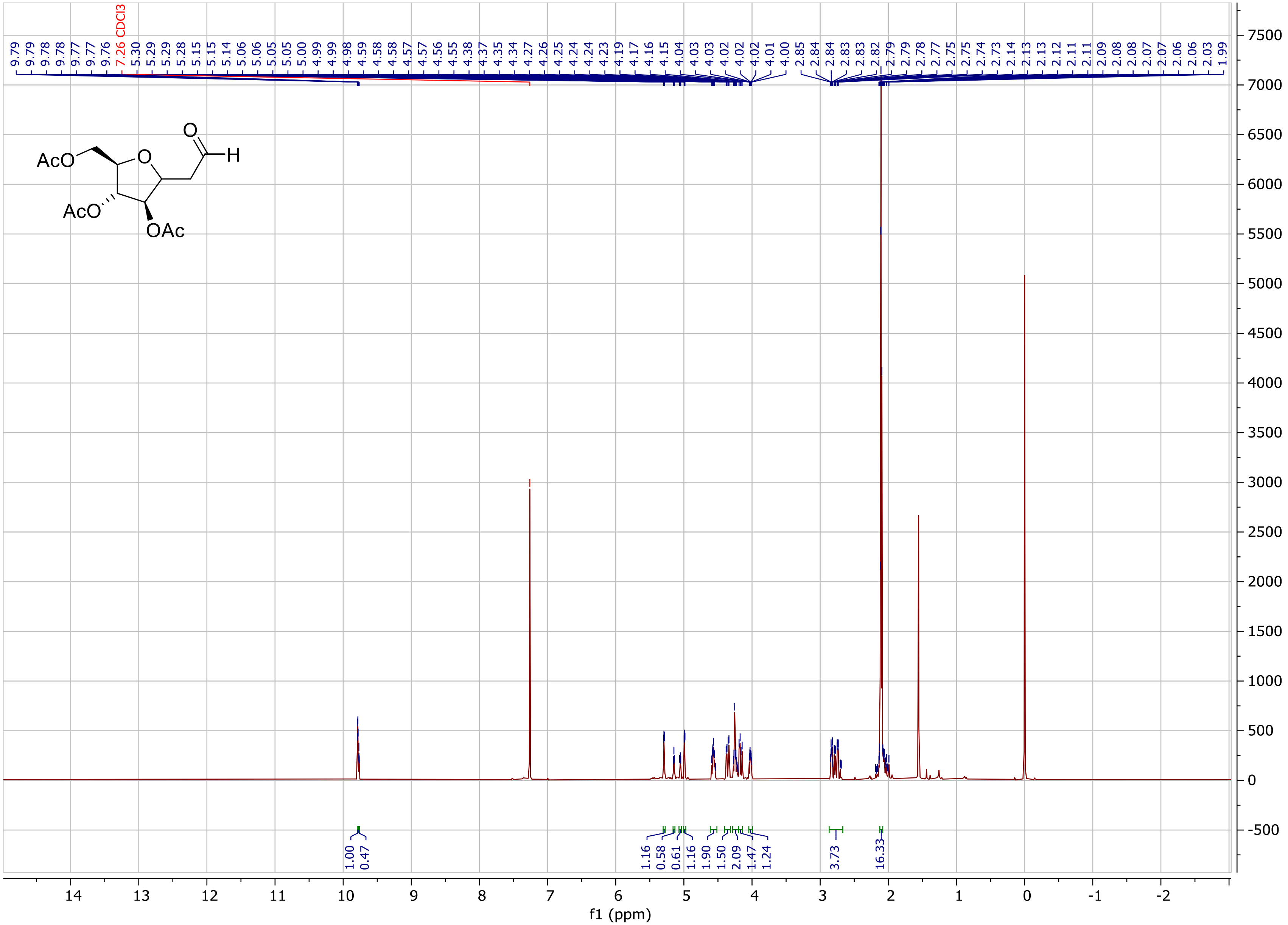

$^{13}C\{^{1}H\}$ NMR (100 MHz, d1-chloroform)

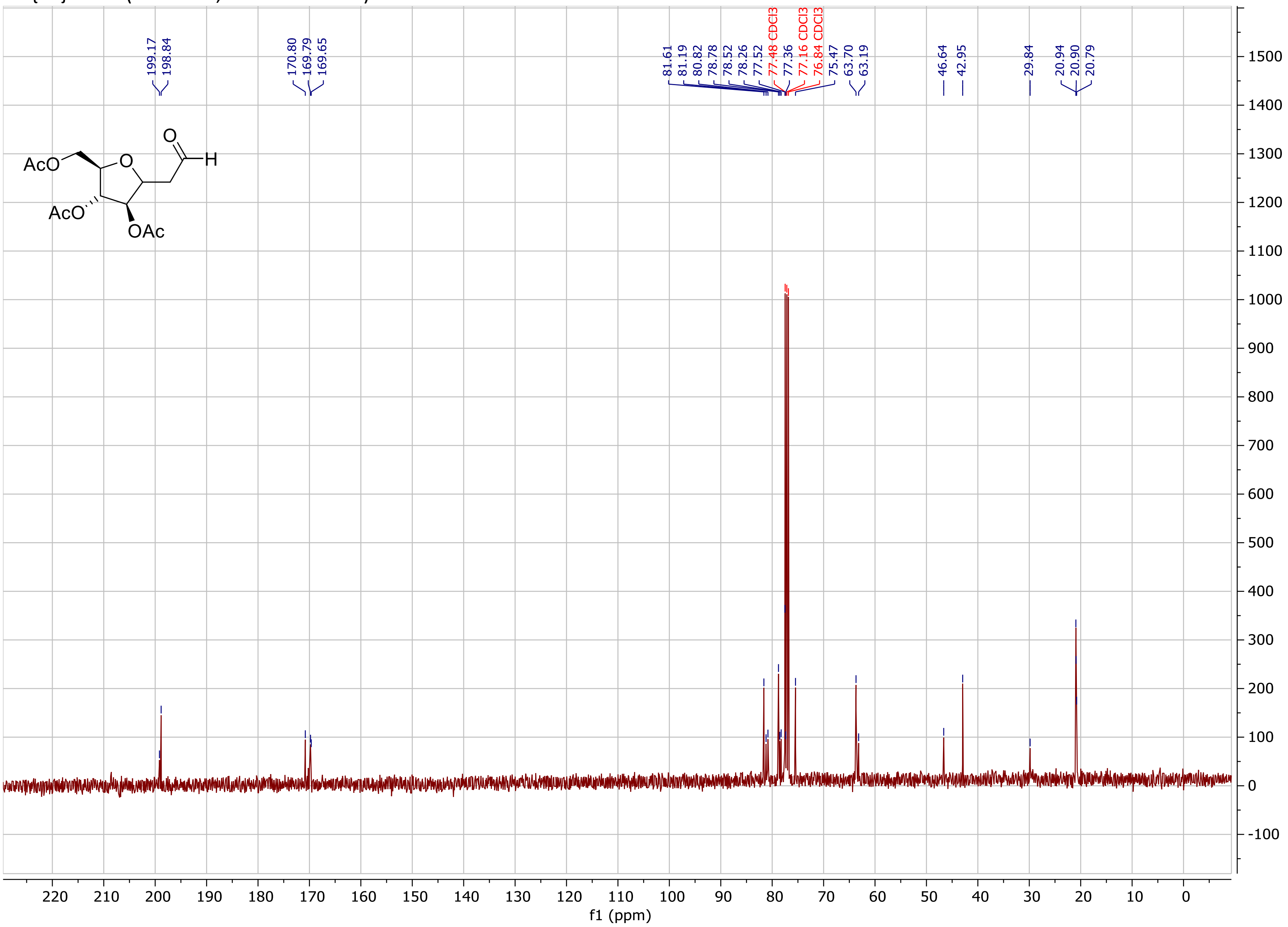

COSY NMR (400 MHz, d1-chloroform)

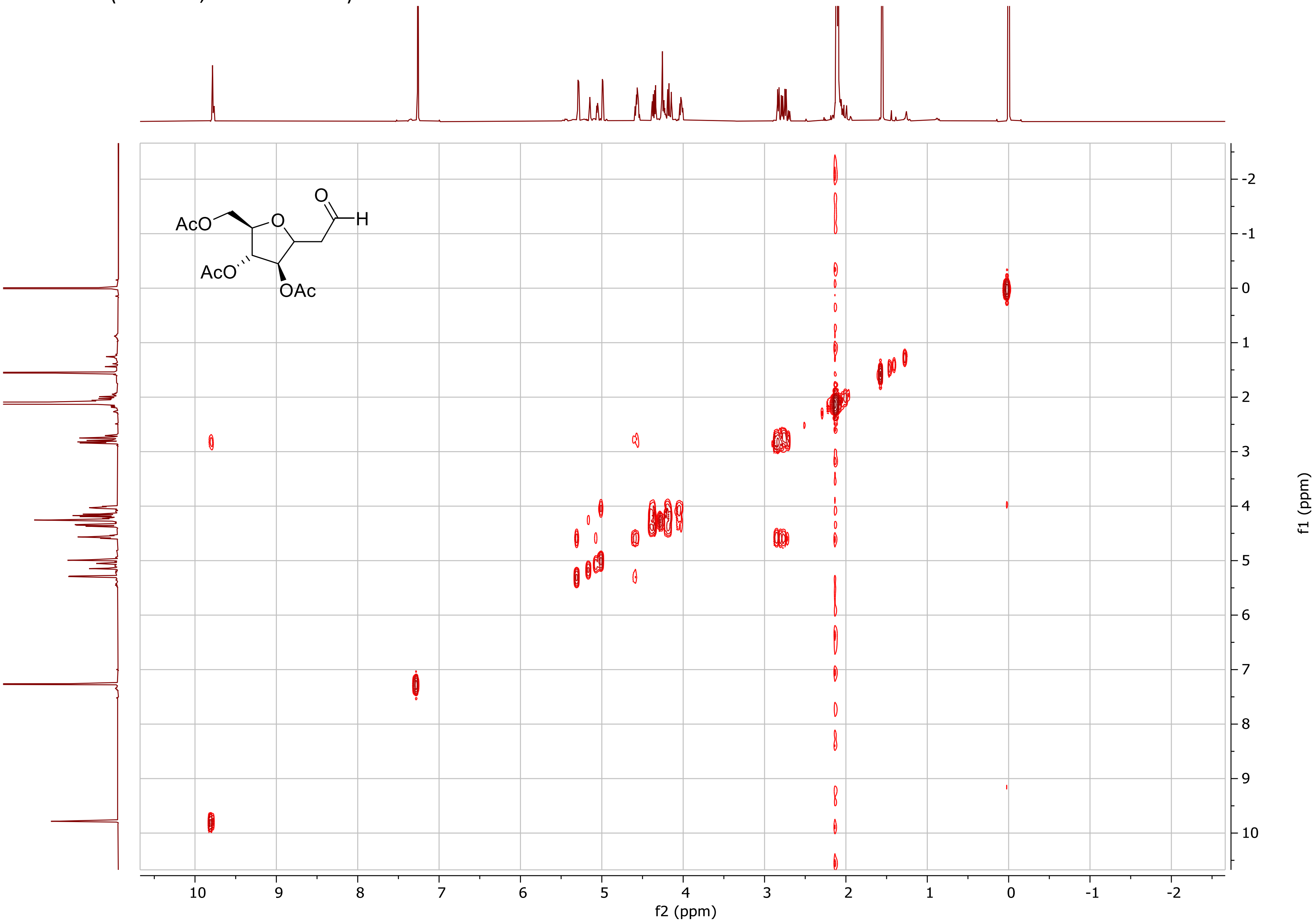

HSQC NMR (400 MHz, d1-chloroform)

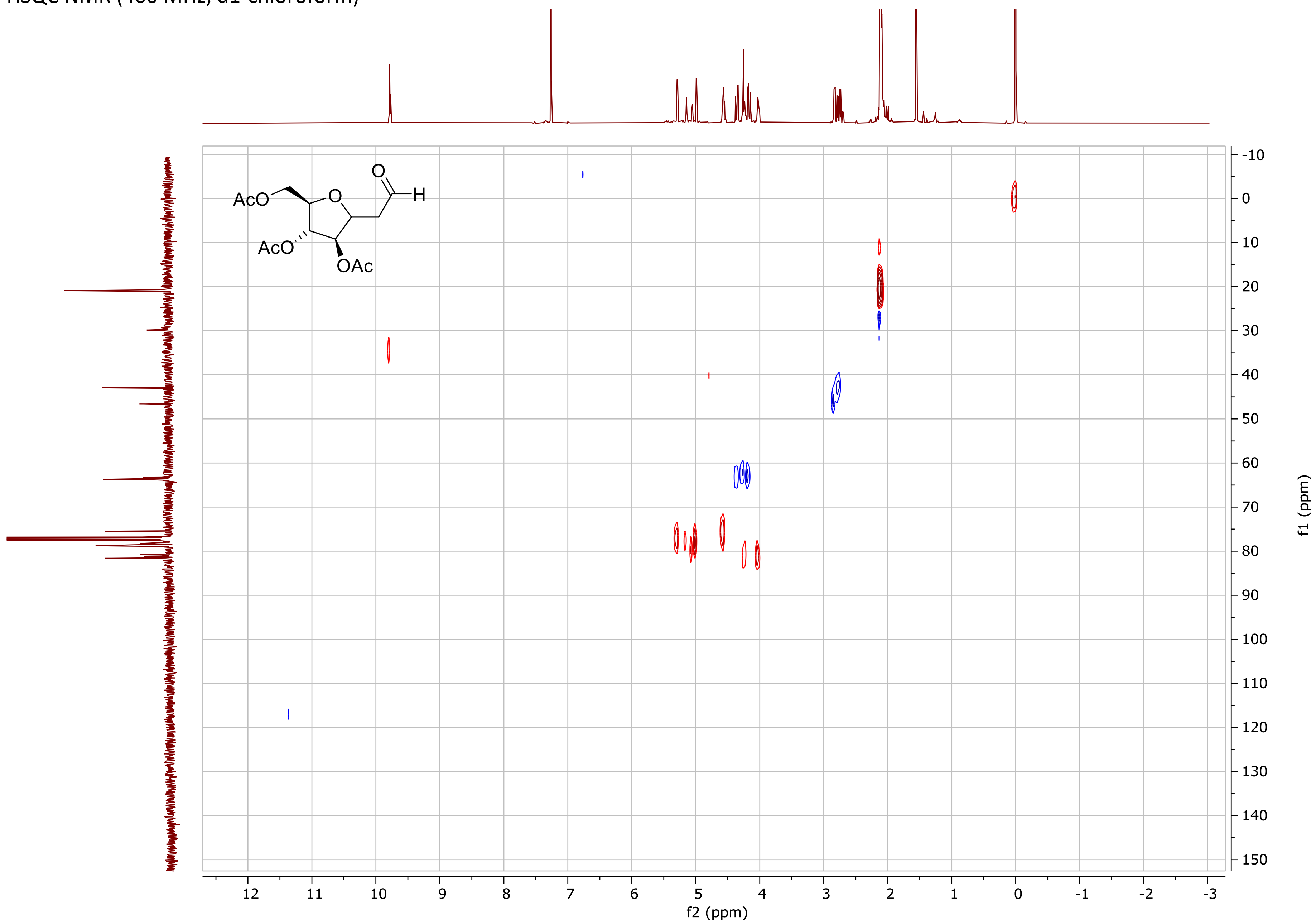

**NMR spectra used to characterize compound 18**

$^{1}$H NMR (400 MHz, d6-acetone)

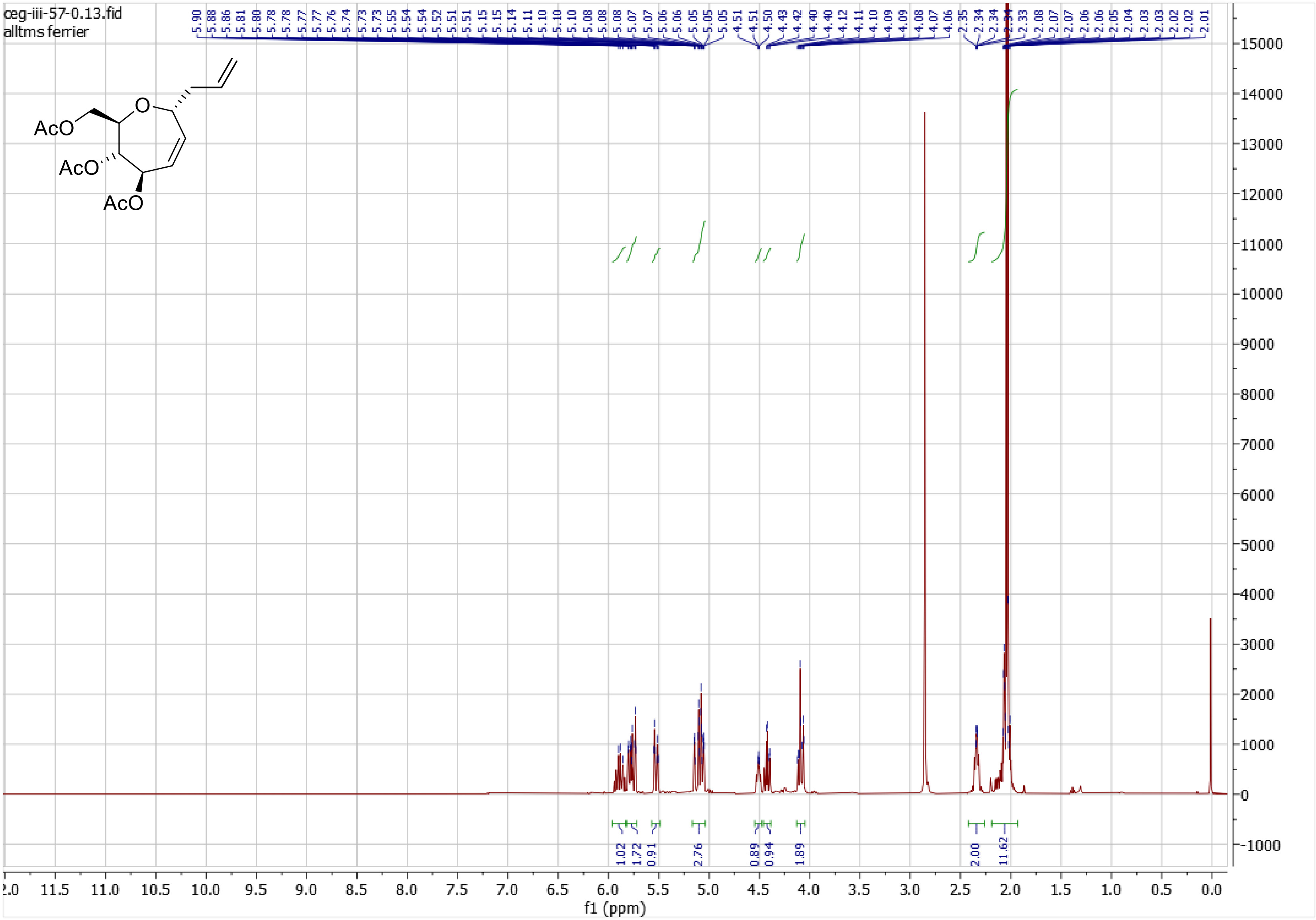

$^{13}C\{^{1}H\}$ NMR (400 MHz, d6-acetone)

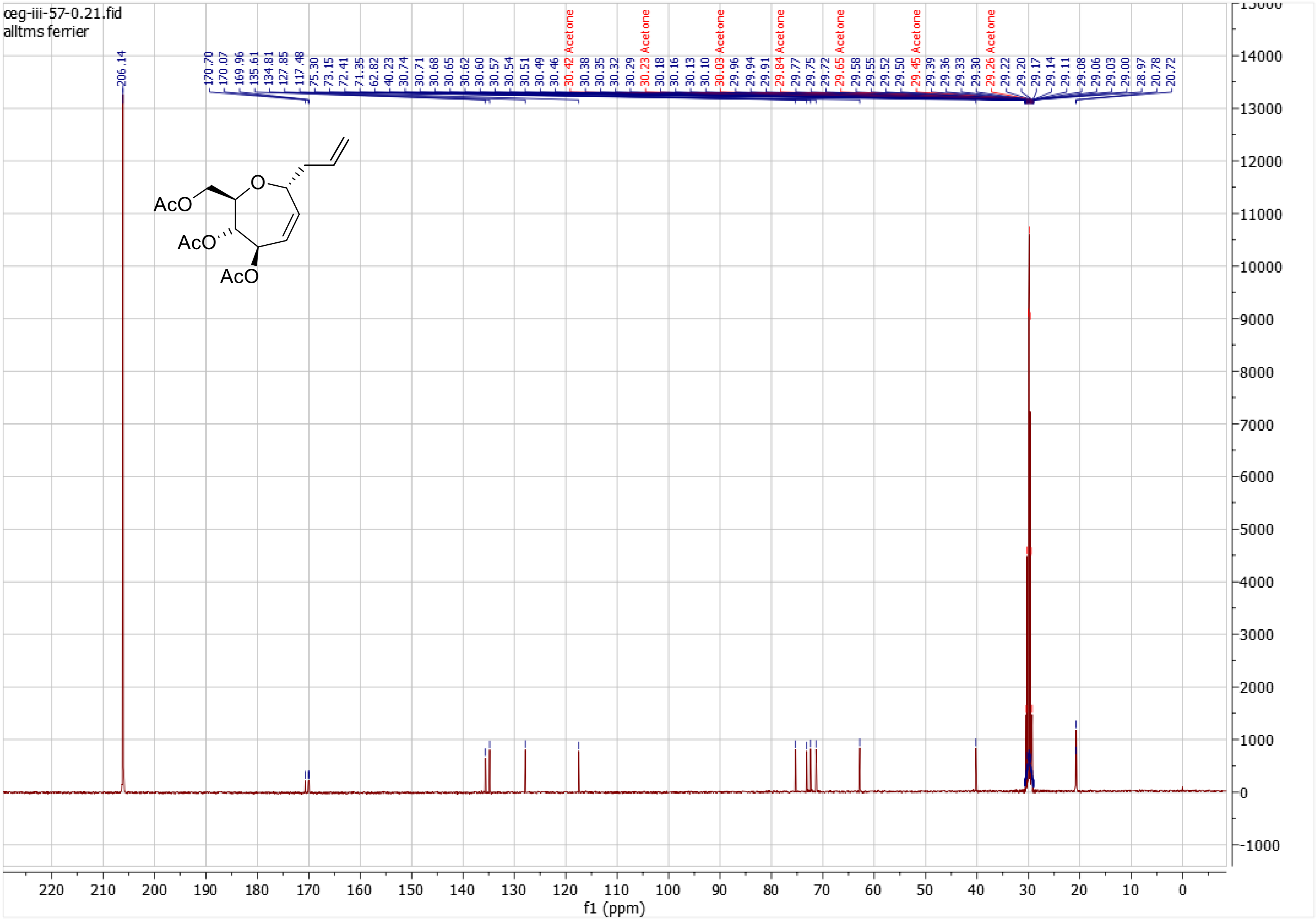

COSY NMR (400 MHz, d6-acetone)

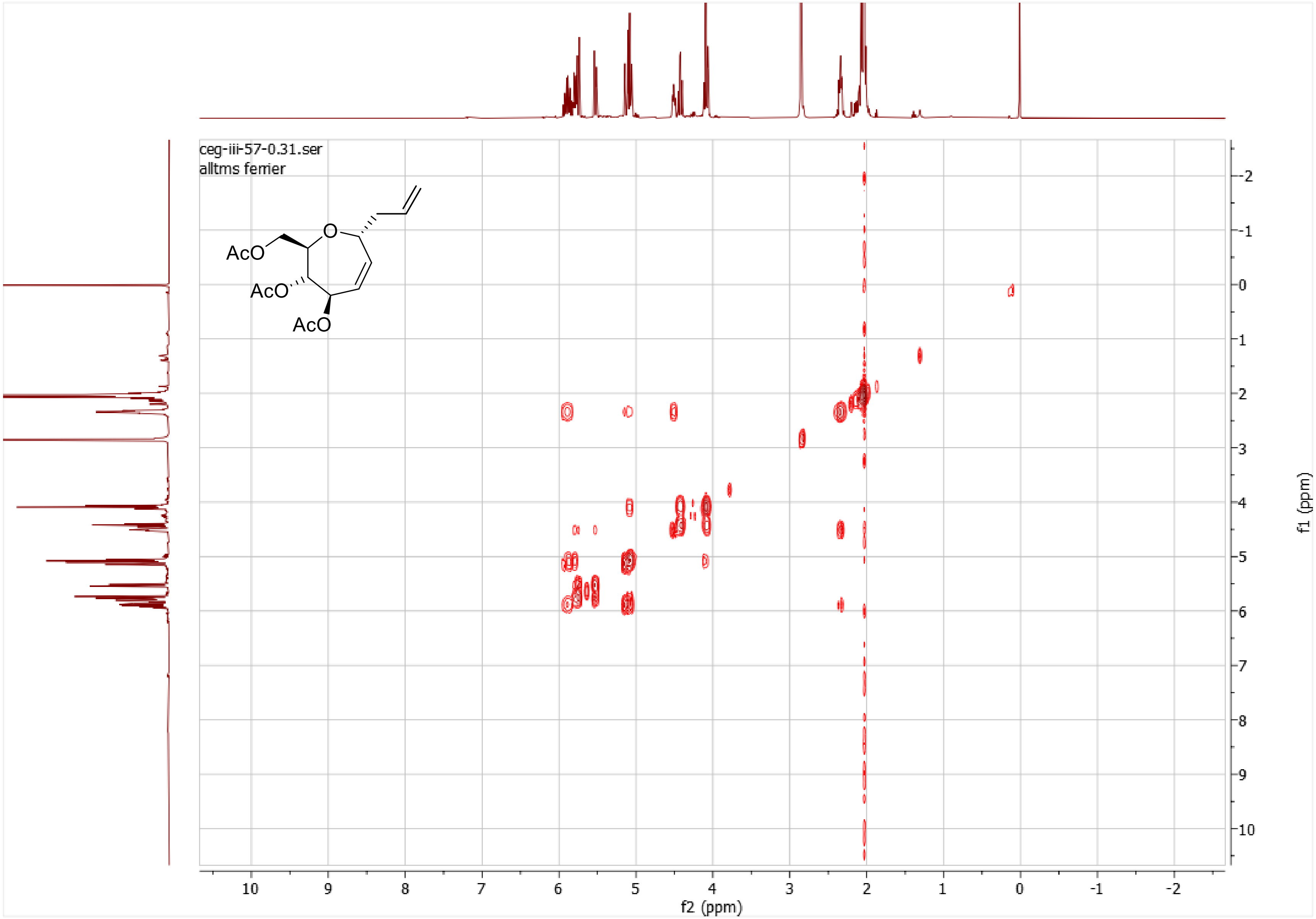

HSQC NMR (400 MHz, d6-acetone)

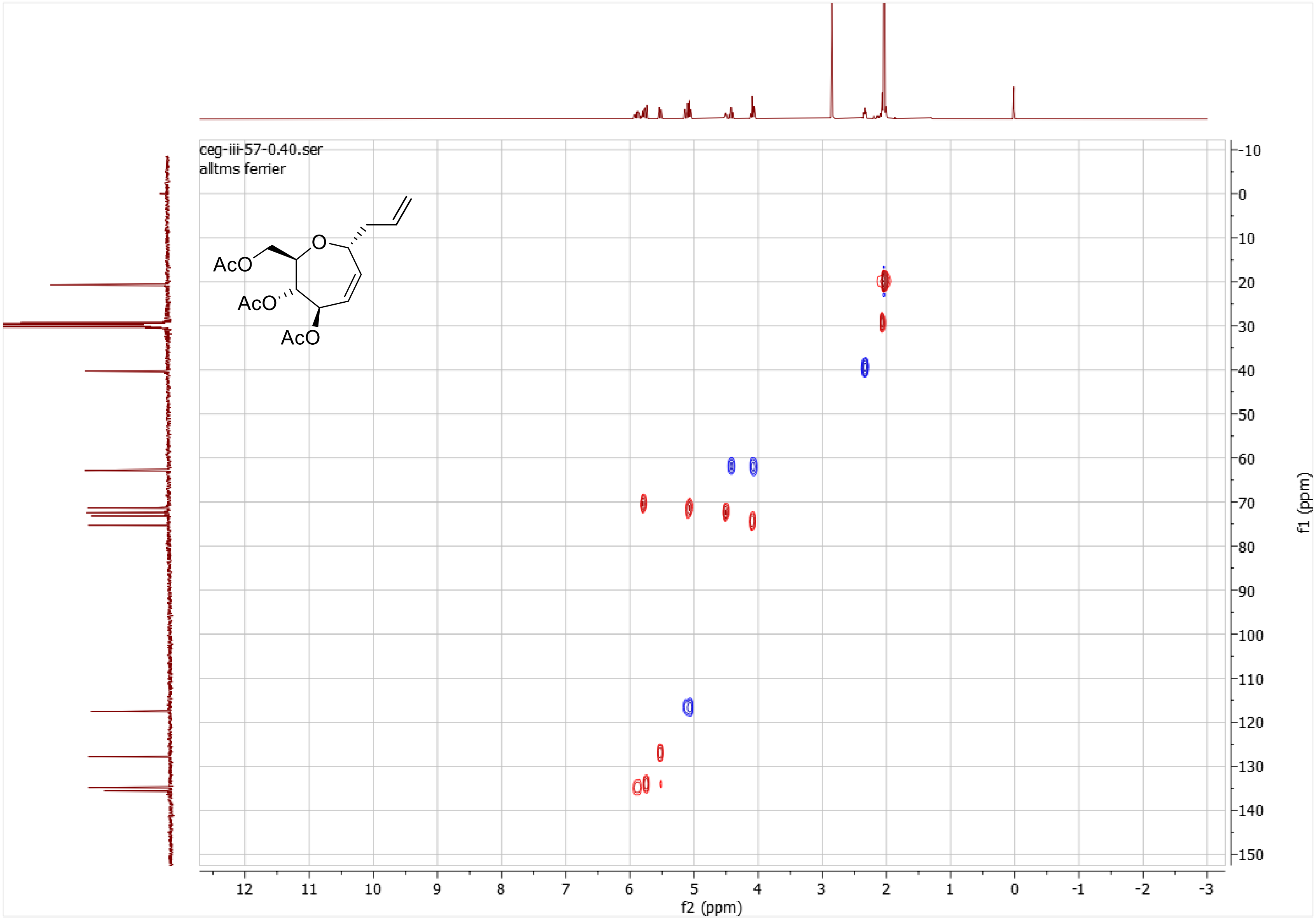

NOESY NMR (400 MHz, d6-acetone)

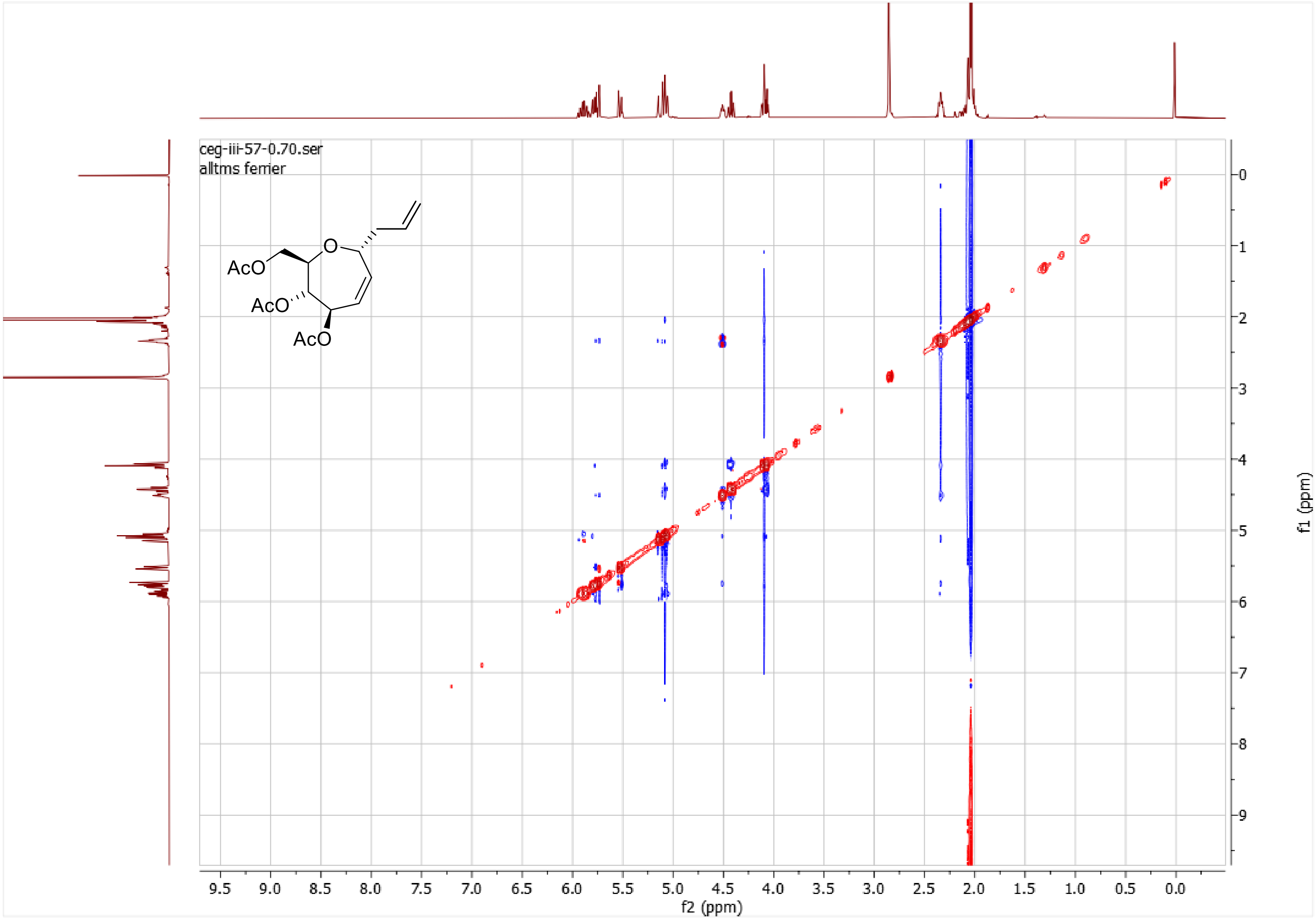

1D NOE NMR (400 MHz, d6-acetone)

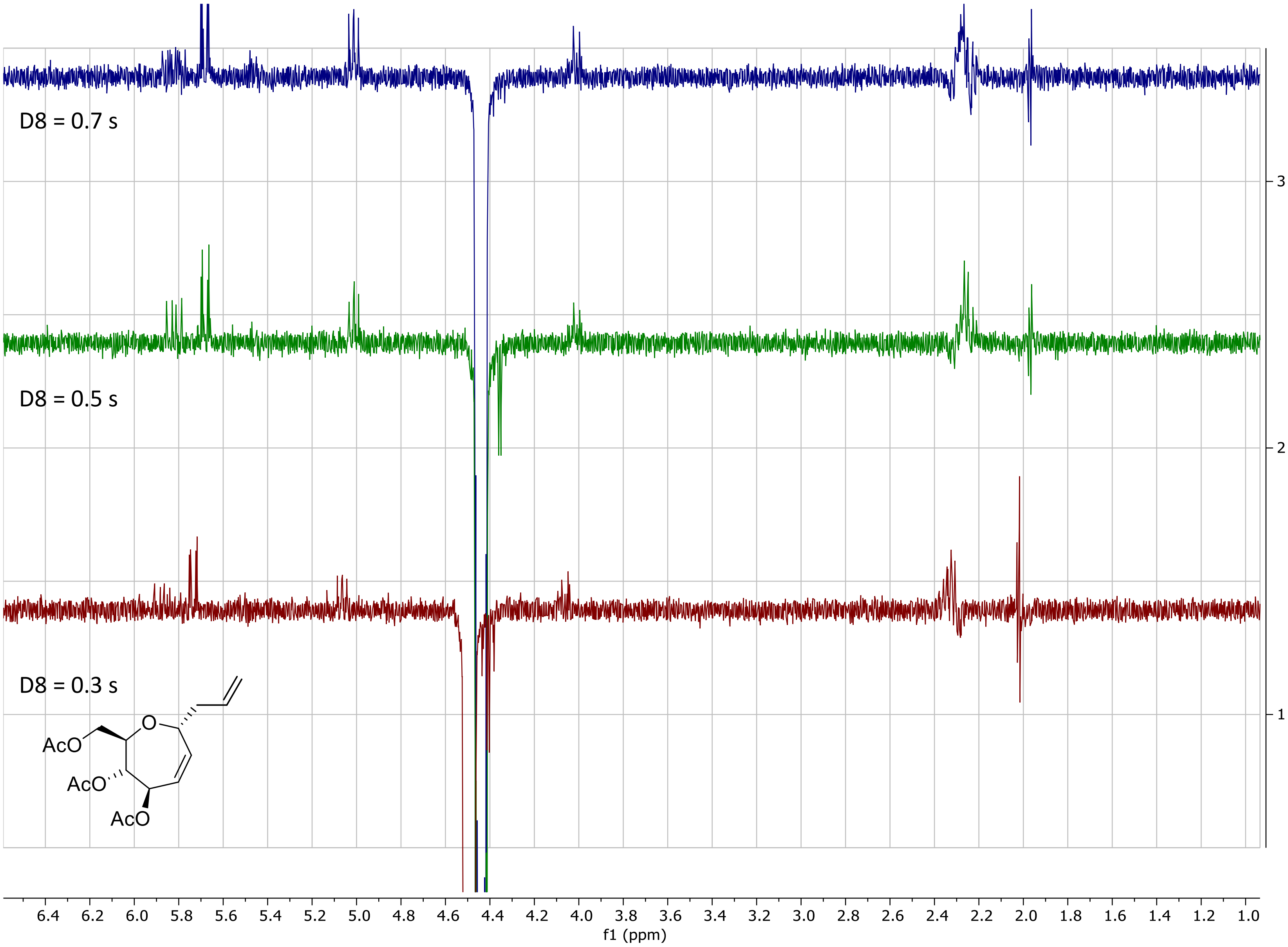

1D TOCSY (400 MHz, d6-acetone)

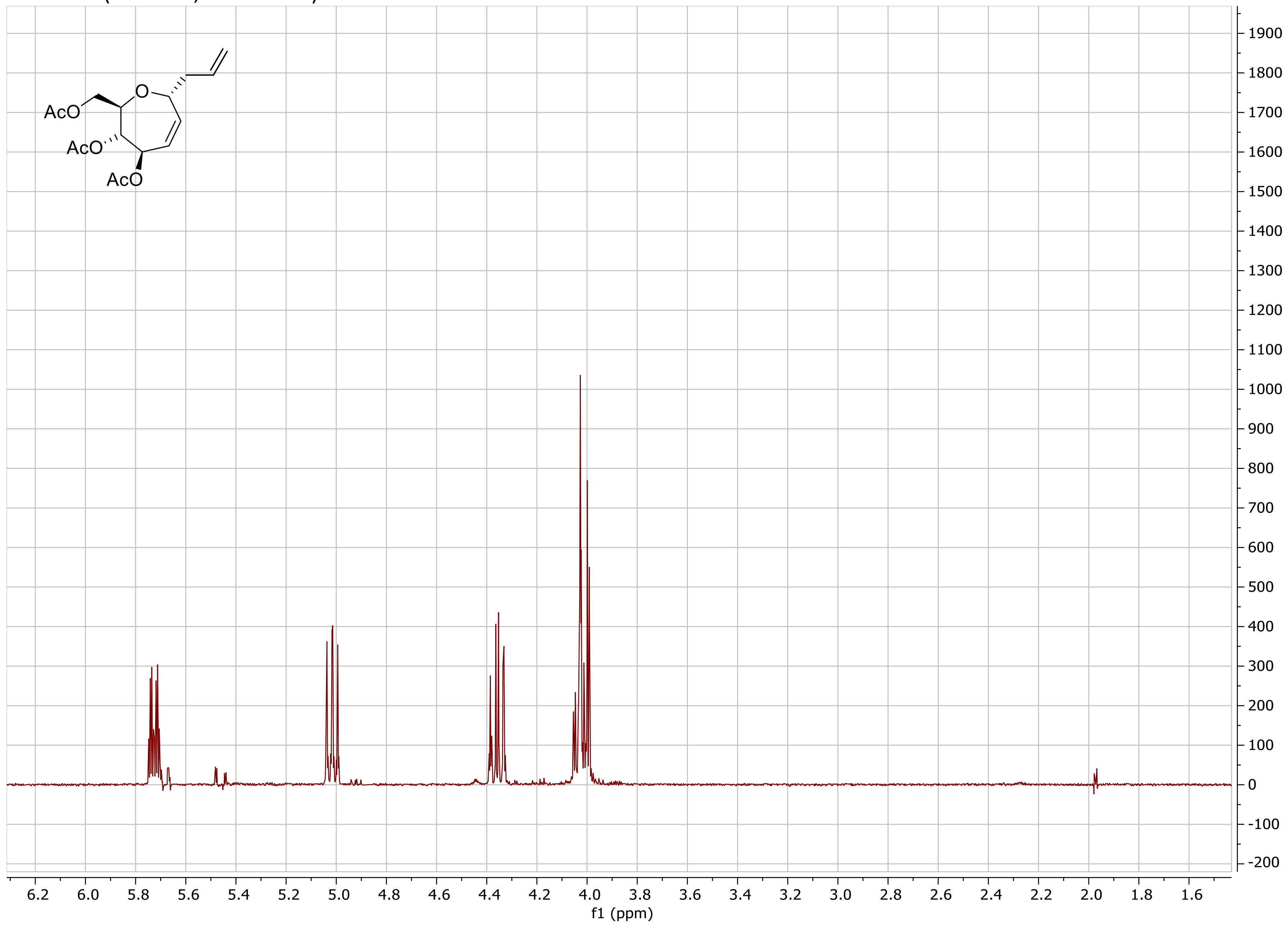

Overlay of 1H, NOESY, and TOCSY

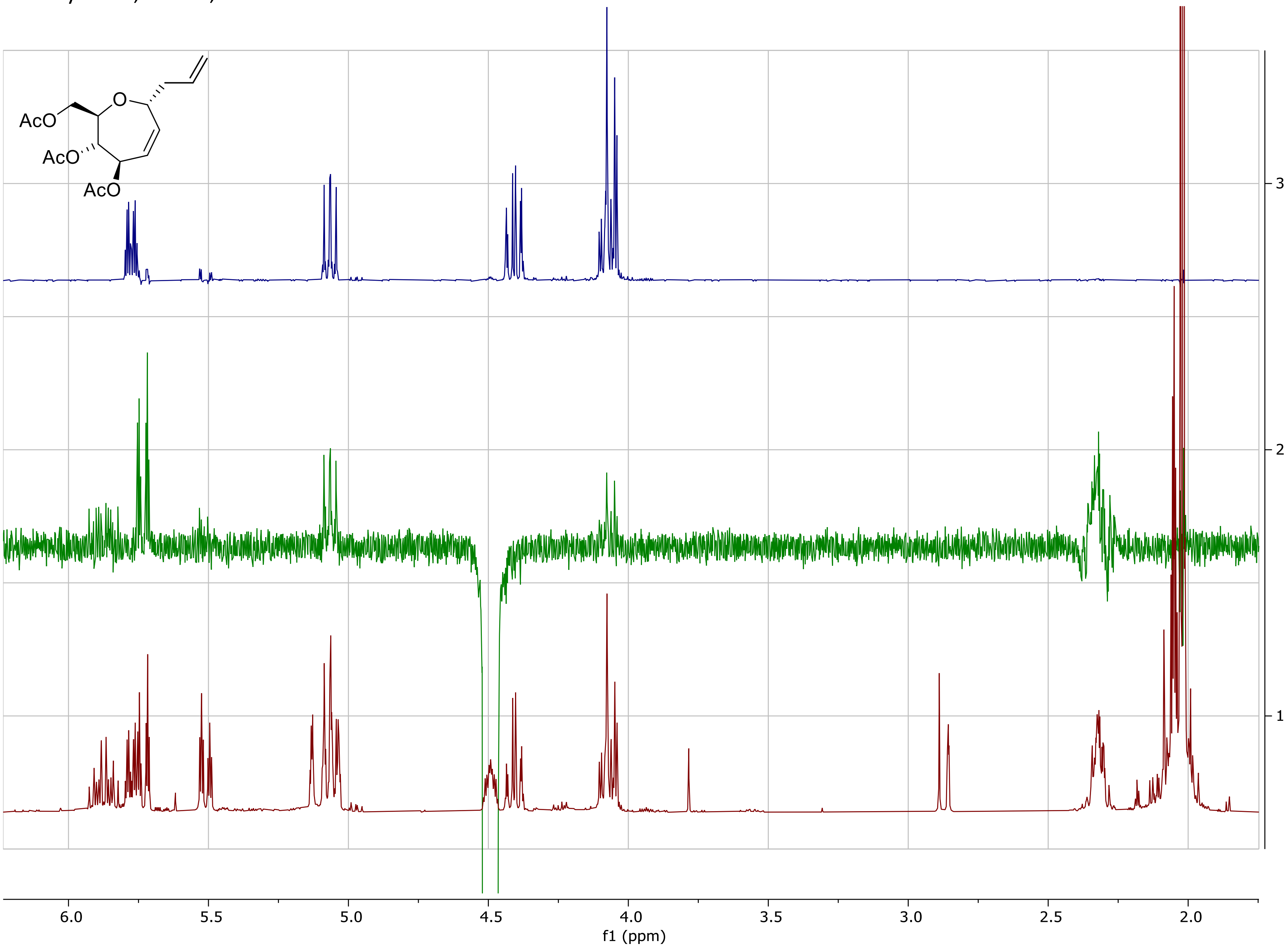

Supplement: Supplementary file 1 — jo3c00079_si_001.pdf [file jo3c00079_si_001.pdf]
